# Key actors in behavioral health services availability and accessibility research: a scoping review bibliometric analysis

**DOI:** 10.1007/s44192-024-00068-3

**Published:** 2024-05-03

**Authors:** Cole Hooley, Danielle R. Adams, Wai Yan Ng, Carrie L. E. Wendt, Cory B. Dennis

**Affiliations:** 1 School of Social Work Brigham Young University, 2190 JFSB, Provo, UT 84602 USA; 2 Center for Mental Health Services Research Brown School of Social Work and Public Health, Washington University in St. Louis, St. Louis, MO USA

**Keywords:** Mental health, Substance use, Bibliometric analysis, Availability, Accessibility

## Abstract

This bibliometric review aims to identify key actors in the behavioral health services availability/accessibility literature. Coalescing information about these actors could support subsequent research efforts to improve the availability and accessibility of behavioral health services. The authors used a scoping review method and a bibliometric approach. The articles came from Medline, Embase, Web of Science, CINAHL, and PsycINFO. Articles were included if they assessed behavioral health service availability or accessibility quantitatively and were written in English. The final sample included 265 articles. Bibliometric data were extracted, coded, and verified. The authors analyzed the data using univariate and social network analyses. Publishing in this area has become more consistent and has grown since 2002. *Psychiatric Services* and Graduate Theses were the most frequently used publication venues. The National Institute on Drug Abuse, National Institute of Mental Health, and the Veterans Administration funded the most research. The most frequently used keyword was “health services accessibility.” The findings suggest that this literature is growing. There are a few clusters of researchers in this area. Government organizations primarily fund this research. The paper and supplementary materials list the top researchers, publication venues, funding sources, and key terms to promote further behavioral health availability/accessibility research.

**Supplementary Information:**

The online version contains supplementary material available at 10.1007/s44192-024-00068-3.

## Introduction

An overarching goal of the behavioral healthcare system is that everyone who needs help receives it in a timely manner and receives the intended health benefits from it. Over four decades ago, Tanahashi asserted that a service passes through four levels before the population receives the benefits of that service [1]. First, the service must be *available* to the target population. Second, the target population should have *access* to the service. Third, the target population should deem the service *acceptable*. Fourth, the target population should *use* the service. The final stage of the framework is that the target population receives the benefits of the service. Other reviews have focused on the last two levels of the framework [2]. This paper will focus on the first two: *availability* and *accessibility*.

### Definitions

This section provides definitions for the key constructs of this paper. Those constructs are behavioral health services, availability, accessibility, and utilization.

*Behavioral health services* refers to mental health and/or substance use disorder services. These services are intended for specific populations who exhibit need. Those populations are considered target populations.

*Availability* refers to the degree to which a service “exists” for potential recipients. Penchansky and Thomas [3] defined availability as, “the relationship of the volume and type of existing services (and resources) to the clients' volume and types of needs. It refers to the adequacy of the supply of … providers; of facilities … and of specialized programs and services …” [3], p. 128.

*Accessibility* refers to the degree to which a service is reachable by the target population. Accessibility is, “…the relationship between the location of supply and the location of clients, taking account of client transportation resources and travel time, distance and cost” [3], p. 128. With the proliferation of telehealth, access also includes having the necessary technology to connect with services [4].

*Utilization* refers to the target population using a service. Whereas availability and accessibility are factors related to the *potential* use of a service, utilization reflects *actual* use [1]. As such, some have referred to utilization as actual access to differentiate from perceived access [4].

### Need for behavioral health services

There is a substantial need for behavioral healthcare services. In 2020, approximately 53 million adults in the United States experienced a mental illness [5]. That same year, there were around 40 million who experienced a substance use disorder [5]. Mental illness is among the top ten causes for disease burden worldwide and mental illness related disability has increased over the past three decades [6]. Substance use disorders have also increased over the past thirty years [7].

### Utilization of behavioral health services

Behavioral health service utilization, or the use of services, has increased over time [8–11] Researchers have found an increase in the number of individuals who receive outpatient mental health services, with more individuals seeking treatment from specialized mental health providers [10]. Service utilization has increased across the continuum of psychological distress, from those with low distress to those with high psychological distress [10]. Substance use disorder treatment has remained somewhat stable from 2010 to 2019 with increases in specific subgroups (e.g., individuals involved in the legal system) [11].

Many still do not receive services despite the increased utilization of behavioral health services overtime [5]. Of those adults with a mental illness in 2020, about 46% received services. Unmet service needs are even higher for those with substance use disorders. Only 7% of individuals with a substance use disorder received treatment in 2020. However, this percentage is buffered by the high percentage (98%) of individuals with a substance use disorder who reported they did not need treatment [5].

### Accessibility of behavioral health services

The literature about the degree to which behavioral health services are accessible is much smaller than the literature on service utilization and unmet need. This is partly because many studies operationalize access as actually receiving care (i.e., utilization) [12]. However, using utilization as a proxy for access may lead to misestimates of the reachability of services by the target population [4]. Accessibility is distinct from utilization. Aligned with Levesque and colleagues' Patient-centered Access to Care Framework, access is “the *opportunity* to have healthcare needs fulfilled” [13]. One may have proper access to behavioral health services (e.g., able to schedule an appointment in a timely manner), yet never *utilize* them (e.g., by not showing up to an appointment). Importantly, access examines the degree to which the service system *accommodates* each person's characteristics and realities of the service recipients to increase the opportunity to access care [14].

Accordingly, literature on access to care is often organized around barriers or facilitators associated with specific populations gaining access to behavioral health services. At times, the populations are segmented by age; for example, with studies focused on older adults [15] or teens [16]. At other times, the study may be organized by groups who share the same behavioral health condition(s) [17]. There are also other studies using geographic groupings [18]. Further, literature on access to care often focuses on understanding the unmet need, the extent to which individuals perceived a need for care but were unable to attain it, and the reasons individuals were unable to attain behavioral health care [14].

### Availability of behavioral health service

The extent to which services exist, the availability of services, is uneven in behavioral health. For example, clinics offering substance use treatment increased to 60% in one national U.S. study, but crisis services declined by 28% [19]. Another estimate showed a decrease in community mental health service offerings by 14% which was associated with a 6% increase in suicide deaths [20]. So, while there were increases in some services, they were decreases in others.

Availability research is often focused on specific populations or conditions. For example, less than half of the behavioral service facilities in the U.S. offer trauma-specific services [21]. Less than 18% of behavioral health service facilities offer LGBT-specific services [22]. Less than 20% of community providers were sufficiently trained to provide evidence-based PTSD treatments [23]. Around half (51%) of schools in the U.S. provided mental health assessments and 38% provided treatment [24].

### Present study

The present study is different from other behavioral health service reviews. Other behavioral health availability or accessibility reviews have focused on specific populations [25], geographic locations [18], or services [26]. This review includes all populations, geographic locations (i.e., is international in scope, though limited to English), and services. Furthermore, this review is unique in that its purpose is to highlight key actors (researchers, funders, publishers) in the behavioral health services availability/accessibility literature. This paper will use a scoping review and bibliometric approach to map this literature. A scoping review method is appropriate given our focus to map a body of literature and to identify gaps within that literature [27]. Specifically, this paper seeks to answer the following questions:

1. What are the publication trends in research on behavioral health availability and accessibility?
2. Who are the leading authors conducting research on behavioral health availability and accessibility?
3. What are the research team networks conducting research on behavioral health availability and accessibility?
4. What are the primary venues publishing research on behavioral health availability and accessibility?
5. Who are the primary organizations funding research on behavioral health availability and accessibility?
6. What terms are most frequently used in research on behavioral health availability and accessibility?

Answering these questions can support the further development of this literature. Specifically, the answers will help researchers identify potential collaborators, identify venues to search for and publish research with, identify potential funding sources, and identify terms for subsequent searches.

## Materials and methods

### Study selection and analysis

We used a scoping review method to guide this unregistered bibliometric analysis [27]. As such, we adhered to the PRISMA extension for Scoping Review checklist (checklist is in the Supplemental file, Table S6) [28]. This approach outlines five required stages. The first is to articulate the research question, the second is to identify pertinent studies, the third is to select studies for analysis, the fourth is to extract the data, and the fifth is to analyze and report the data.

### Search strategy

We assembled search terms from other reviews [2, 29–33] and consulted with literature search experts to develop our search strategy. The search hedge included four broad constructs joined together with “AND”: behavioral health, behavioral health services, availability or accessibility, and measurement. Each broad construct contained various terms; see the full search hedge in the supplement file (Table S1). We added the measurement constructs to focus the search on empirical studies of availability and accessibility.

We executed the search across multiple databases at three different time points. We searched for articles in five different databases: Medline, Embase, Web of Science Core Collections, CINAHL, and PsycINFO [34]. We ran the search without limiters three times: February 2020, May 2021, and February 2022. After each search, we removed duplicates in EndNote v.9 using guidance from Bramer and colleagues [35].

### Selection criteria

We developed a screening tool to assess articles for subsequent data extraction. We included empirical articles (i.e., no reviews or commentaries) written in English, involving behavioral health outcomes, and measuring service availability or accessibility quantitatively. Qualitative-only studies were not included. We also included dissertations [36]. Articles measuring service utilization, even as a proxy for availability or accessibility, were not included. Using the Tanahashi framework, we conceptualize utilization as distinct from accessibility and excluded articles focused solely on utilization [1].

Using those inclusion criteria, we screened title/abstracts. We used Abstrackr [37] to manage the screening process for the first search and Rayyan [38] for the two search updates. Two screeners independently assessed articles for the initial search. The screeners would discuss discrepancies, and a third screener would break ties [39]. One screener assessed all articles, and a second screener assessed excluded articles for the two search updates [40]. Any discrepancies found during the search updates were advanced to full-text screening.

### Data extraction

We extracted bibliometric data from each full-text article. Like other reviews [41], a primary coder extracted data from each article and a second coder reviewed the extraction making notes where there were discrepancies. We extracted: author names, journal names, keywords, year published, and funding sources into a spreadsheet. Extractors resolved discrepancies and used a third extractor as needed. The bibliometric information from the EndNote files and additional extracted variables in the spreadsheet were imported to Stata v.17 and Gephi for analysis. A table with all articles and their extracted variables is available in the supplemental file 1 (Table S7).

### Data analysis

We used a mix of descriptive statistics and other approaches to address the research questions. For publication trends, we used descriptive analysis to plot the number of publications over time. For leading authors, we used descriptive analysis to determine the number of first-author and co-author articles published by each researcher. Aligned with previously used methods, we calculated a combined weighted counting score. We used a fractional analysis to assign a 20% bonus to the first author and divided the remainder among the co-authors [42]. For solo-authored papers the authors were given a score of 1, which is the highest score for a single article. For articles with more than one author the following calculations were used to generate the co-author score: ((1/number of authors)-((1/number of authors)*0.2)) and to generate the first-author score (0.2 + (1/number of authors)-((1/number of authors)*0.2))). The sum of co-author and first author scores for each article is 1. Then we took the sum of all the author’s scores to produce the combined weighted counting score (CWC) as seen in Table 1. This scoring procedure acknowledges that intellectual contribution to co-author papers vary. It offers greater specificity of intellectual contribution beyond article count [42]. We used social network analysis to visualize authorship networks. The visualization was created using Gephi. Like other bibliometric analysis papers, we used a cut-off of three articles to determine which authors would be included in the social network analysis [43, 44]. We included all authors associated with the co-authoring network of authors who had three or more articles. The author’s node size increased with the number of publications, and the connecting line thickness was based on the number of co-authored papers between the authors.

We used descriptive statistics to determine which journals published the most articles, and we also compared that number with the journal’s impact factor per the Journal Citation Report and CiteScore [45]. Funding sources were analyzed descriptively to identify the primary funding sources. We also coded the funding sources to identify the sectors most involved in funding this research (government, foundations/associations, universities, industry, mix, unknown, none, or not reported). Those sectors were then analyzed descriptively. Scholarly databases manage article keywords differently. For instance, some scholarly databases catalog the keywords provided by the author, whereas others add relevant subject terms. EndNote does not distinguish between these types, so we pooled all keywords. We retained the formatting of the keywords as they appeared in the database citations; for example, some journals would use “/,” and others would use “—” to format phrases that were intended to be together. Not all the keywords applied to the availability and accessibility constructs. Three authors identified the keywords which applied to those constructs for frequency reporting.

## Results

### Study characteristics

**Fig. 1:**
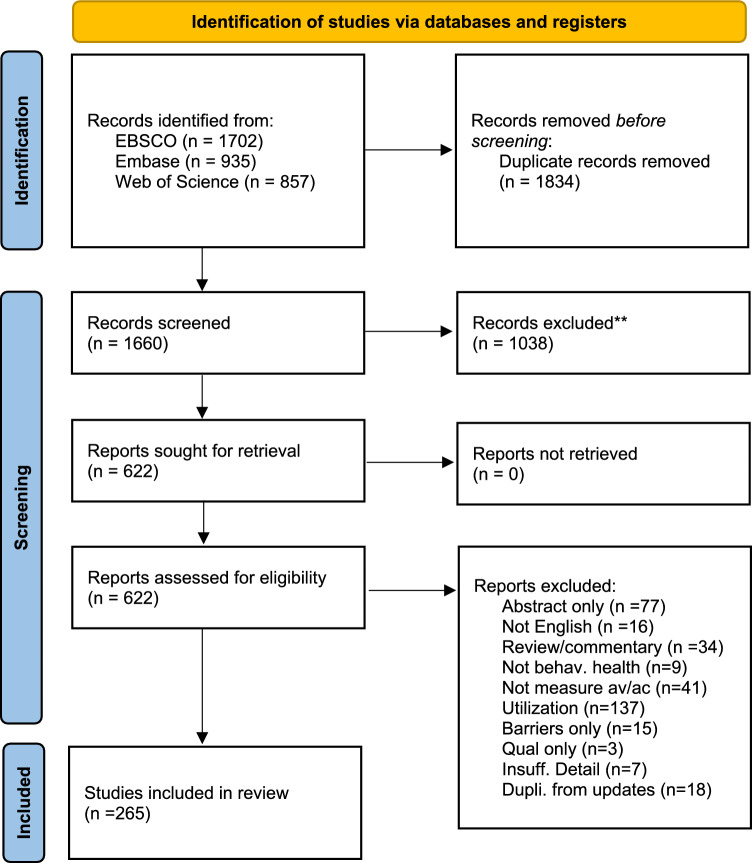
PRISMA flow diagram. From: Page MJ, McKenzie JE, Bossuyt PM, Boutron I, Hoffmann TC, Mulrow CD, et al. The PRISMA 2020 statement: an updated guideline for reporting systematic reviews. BMJ 2021;372:n71. https://doi.org/10.1136/bmj.n71. For more information, visit: http://www.prisma-statement.org/

Figure 1 is the PRISMA flowchart showing the selection of articles for this analysis. There were 1660 unique articles from the combined searches after duplicates were removed. The analytic sample for the paper was 265 articles after the screening process. The largest exclusion step was at the title/abstract review. During the full-text review, the most frequent reason for exclusion was because papers were measuring utilization rather than availability and accessibility. There were articles that would use availability and accessibility terms but would define them or operationalize them in such a way that they were measuring utilization.

### Publication trends

**Fig. 2:**
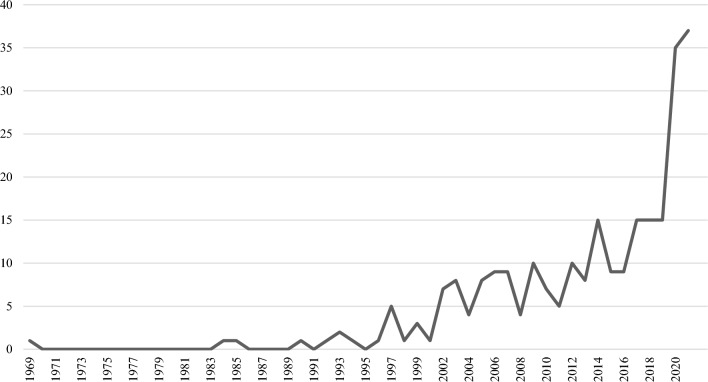
Number of behavioral health service availability or accessibility publications from 1969 to 2021

The number of publications in this area, like many areas of study, is trending upward (see Fig. 2). The earliest publication in our sample is 1969. More consistent publishing began around 2002 with vacillations trending upward. The largest increase was between 2019 and 2020. Articles from 2022 were not included in this visualization because the search was conducted in February 2022. The search date would artificially decrease the number of articles because it only captured articles from the first part of the year.

### Leading authors

Across the 265 articles, there were 1031 unique authors. The average number of articles per author was 1.1 (SD = 0.5), with a range of 1 to 7. The average number of authors per article was 4.4 (SD = 2.6), with a range of 1 to 16. Table 1 provides a list of authors with three or more publications along with the authors’ combined weighted counting score and number of 1st and co-author publications. The full list of authors and their scores is in the supplemental file 1(Table S2). These authors represent researchers who have produced the most articles in the behavioral health service availability or accessibility literature. The table is sorted by the combined weighted counting score. The combined weighted counting score accounts for number of articles, number of co-authors, and paper role (first vs. co-author). Accounting for these factors provides a more precise measure of scholarly contribution. The table also includes the year of the most recent article published by the author. The dataset includes articles from 1969 to February of 2022. There are 24 authors who have published three or more articles. Of those authors, 50% have published an article in the past five years.

**Table 1:** Authors with three or more publications in behavioral health availability or accessibility

Author	Articles	CWC^1^	1st-author	Co-author	Most recent article in dataset^2^
Knudsen, HK	7	3.60	5	2	2015
Abraham, AJ	7	2.56	5	2	2021
Roman, PM	6	1.67	0	6	2015
Andrews, CM	5	1.89	2	3	2021
Cummings, JR	5	1.59	3	2	2021
West, JC	5	1.04	2	3	2016
Fortney, JC	5	1.00	2	3	2021
Druss, BG	4	0.79	0	4	2017
Rae, DS	4	0.54	0	4	2014
Wen, H	3	1.03	2	1	2015
Ducharme, LJ	3	1.00	1	2	2007
McCarthy, JF	3	0.82	2	1	2010
Yarbrough, CR	3	0.76	0	3	2021
Guerrero, EG	3	0.72	1	2	2021
McBain, R	3	0.65	1	2	2021
Pyne, JM	3	0.64	2	1	2021
Wilk, JE	3	0.61	1	2	2014
Kilbourne, AM	3	0.59	0	3	2010
Busch, SH	3	0.56	0	3	2022
Rieckmann, T	3	0.56	0	3	2018
Moscicki, EK	3	0.55	1	2	2016
Regier, DA	3	0.43	0	3	2010
Saloner, B	3	0.39	0	3	2022
Fischer, EP	3	0.26	0	3	2020

### Author social networks

**Fig. 3:**
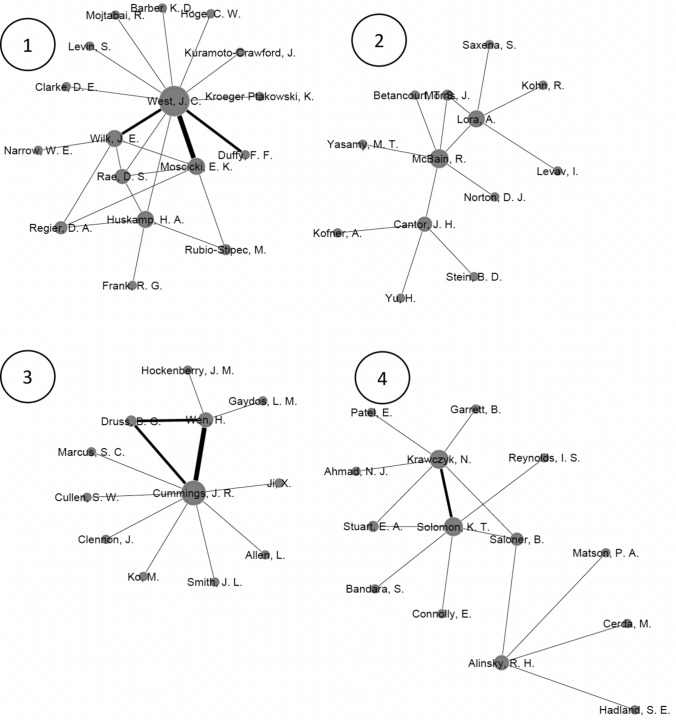

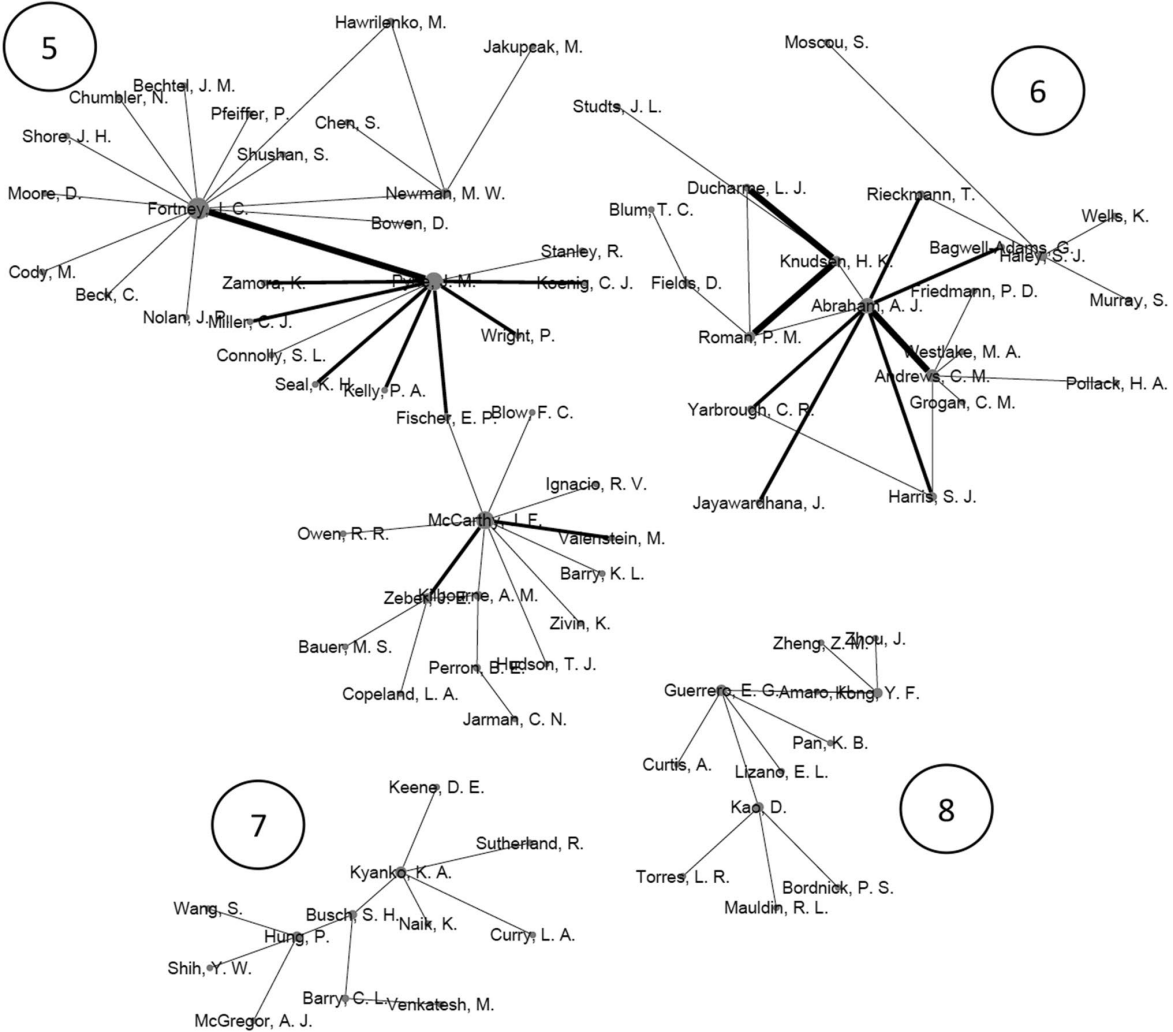
Social networks for authors with three or more publications and their co-authors in the behavioral health availability and accessibility literature

Figure 3 represents the co-authorship networks for the sample of articles. In total, there were eight networks that included all authors with three or more articles. Each network is numbered 1–8 in the figure. The authors within a network are connected because of their collaboration on articles. The connecting lines between authors indicate co-authorship on an article. Thicker lines denote a greater number of co-authored articles, and larger node size indicates a greater number of articles. There are 140 nodes among the eight networks which means there are 140 different authors in these networks. As noted in the method’s section, authors were included in the network analysis if they co-authored a paper with an author who had 3 or more publications in the behavioral health service availability and/or accessibility literature. There were 24 authors (see Table 1) who published 3 or more articles. That group represents 17% of the overall author count across the eight networks. There are 116 authors in the networks who published fewer than 3 articles in this area. There were 149 connecting lines, meaning there are 149 unique connections between authors. The graph density coefficient was 0.015. The average clustering coefficient among the eight networks is 0.247. The modularity coefficient is 0.866.

### Journal information

There were 159 journals that published articles measuring the availability or accessibility of behavioral health services. Approximately 76% of those journals have published only one article in this area. Table 2 outlines the top 20 publication sources along with each journal’s Journal Citation Report (JCR) score and their CiteScore. The full list of publication venues for the sample of articles is in the supplemental file (Table S3). Psychiatric Services published the most articles. Interestingly, the second-highest publication source was graduate student theses/dissertations. The journal with the highest JCR and CiteScore that published articles in this area was JAMA Psychiatry.

**Table 2:** Top publication sources for behavioral health availability accessibility articles, lifetime and in the last five years with indicators of journal quality

Journal	Articles 1969–2022	Articles 2018–2022	jcr 2021	Citescore 2021
Psychiatric Services	22	8	4.157	4.6
Graduate theses	13	3		
Health Services Research	7	3	3.734	4.8
Drug & Alcohol Dependence	6	4	4.852	6.2
Health Affairs	5	2	9.048	8.1
Journal of Substance Abuse Treatment	5	4	3.917	5.3
The Journal of Behavioral Health Services & Research	5	0	1.475	3.3
Administration and Policy in Mental Health and Mental Health Services Research	4	3	2.619	
American Journal of Public Health	4	2	11.576	6.9
Military Medicine	4	1	1.563	1.6
Psychological Services	4	4	3.097	4.3
BMC Health Services Research	3	3	2.908	3.5
BMC Psychiatry	3	2	4.144	4.7
Children and Youth Services Review	3	1	2.519	2.5
JAMA Psychiatry	3	0	25.936	26
Journal of General Internal Medicine	3	3	6.473	4.6
Journal of Rural Health	3	1	5.667	5.1
Medical Care	3	2	3.178	4.9
Plos One	3	3	3.752	5.3
Substance Use & Misuse	3	0	2.362	2.6
BMJ Open	2	2	3.007	
BMJ Open Quality	2	2		1.8
JAMA Network Open	2	2	13.360	11.1

*JCR* Journal Citation Reports, *CiteScore * Elsevier CiteScore metric

Table 2 also lists the number of articles for the period of 2018–2022. Given that the time horizon for the article sample spans from 1969, we assessed publication venue for the past five years of the data (2018–2022) to see which venues have been publishing more recently. While many of the journals remained on the top venues list, the order changed. Using either time horizon, Psychiatric Services remained the most frequent publisher. In addition, there were three journals who were in the top 20 journals lifetime (1969–2022) who have not published an article in the last five years the Journal of Behavioral Health Services & Research, JAMA Psychiatry, and Substance Use & Misuse. There were also three journals who were not in the top 20 lifetime list who have started publishing more frequently from 2018 to 2022 BMJ Open, BMJ Open Quality, & JAMA Network Open.

### Funding sources

Funding for the sample of articles varied. Around 26% of the articles did not report funding, and another 4% reported not being funded. The number of funding sources across articles ranged from zero to 13. Across the dataset there were 177 unique funders in the dataset. Government agencies were the largest funding source followed by foundations/associations. Among articles that were funded, government agencies funded 62% of them. Foundations came in at a distant second with 16%. The National Institute on Drug Abuse and the National Institute of Mental Health were the top research funders. Table 3 lists funders listed as supporting studies in three or more articles. The table also lists the year of the most recent article that was funded by that organization. The list of all funders is in the supplemental file 1: (Table S4).

**Table 3:** Funding sources by category for funders who supported three or more articles

Category	Organization	Articles (N)	Year^1^
Government	National Institute on Drug Abuse	28	2021
	National Institute of Mental Health	16	2022
	Veterans Administration	13	2020
	Agency for Healthcare Research and Quality	8	2021
	National Institute of Child Health and Human Development	5	2022
	National Institute on Alcohol Abuse and Alcoholism	5	2021
	Canadian Institutes of Health Research	3	2020
	Health Resources and Services Administration	3	2021
	National Center for Advancing Translational Sciences	3	2020
	National Institute on Minority Health and Health Disparities	3	2021
	Patient Centered Outcomes Research Institute	3	2021
Foundations	The Robert Wood Johnson Foundation	5	2013
	American Psychiatric Association Foundation	3	2016
	Michael Smith Foundation	3	2020

^1^ = year of most recent article that was funded by this organization

The list of most frequent government funders shows that in the most recent years of the dataset (up to 2022), there were articles still being supported by these organizations. The timing for supported articles from foundations was different. For example, the last article supported by the Robert Wood Johnson Foundation was in 2013. This might suggest an earlier interest in this area, but not recent support. There are many other funders listed in the supplemental file. For the last five years of the dataset (2018–2022), the largest funder of research in this area was Government organizations followed by Industry and then Foundations.

### Keywords

The search covered a wide range of behavioral health studies assessing availability and accessibility. As such, the range of keywords provided by the authors, or the database platforms was broad. There were 3376 terms applied to the 265 articles. The mean number of keywords for the sample of articles was 8.7, ranging from 0 to 41. In all, there were 1555 unique keywords used. Not all the keywords applied to the availability and accessibility constructs. Table 4 lists the keywords applicable to availability and accessibility used five or more times as well as behavioral health/behavioral health services terms used five or more times. The full list of keywords is in the supplimental file 1(Table S5). We have also included the year of the most recent publication that used those keywords as a recency indicator. There are 41 keywords in the table and 88% of the terms have been used in the past five years of the dataset.

**Table 4:** Most frequently used key terms in the behavioral health availability or accessibility literature

Service availability or accessibility terms with five or more articles using them		
N	Terms	Most recent article^1^
63	Health services accessibility	2021
26	Health services accessibility/statistics & numerical data	2018
24	Medicaid	2022
11	Access to care	2021
11	Health services needs and demand	2020
10	Socioeconomic factors	2022
9	Health care access	2022
9	Healthcare disparities	2020
9	Insurance coverage	2021
8	Access	2022
8	Health services accessibility—statistics and numerical data	2018
8	Waiting list	2020
7	Health care delivery	2020
7	Mental health services/supply & distribution	2012
7	Referral and consultation	2021
5	Health care costs	2018
5	Health insurance	2020
5	Medicare	2021

Behavioral health/behavioral health service-related terms with five or more articles using them		
N	Terms	Most recent article^1^
45	Mental health services	2022
35	Mental health	2022
17	Mental health services/statistics & numerical data	2020
11	Depression	2021
11	Mental disorders	2016
11	Substance abuse	2021
8	Drug abuse	2013
7	Mental disorders/therapy	2019
7	Substance abuse treatment centers/statistics & numerical data	2020
6	Anxiety	2021
6	Community mental health services	2018
6	Counseling	2021
6	Substance abuse treatment centers/organization & administration	2020
6	Substance use treatment	2021
6	Substance-related disorders/therapy	2020
5	Drug therapy	2021
5	Mental disorders/epidemiology	2012
5	Mental health care	2020
5	Opioid use disorder	2022
5	Substance use disorders	2020
5	Substance use disorders—therapy	2016
5	Substance use rehabilitation programs	2019
5	Substance-related disorders/rehabilitation	2020

^1^ = database includes articles from 1969 to Feb. 2022

## Discussion

This bibliometric analysis sought to map the behavioral health availability and accessibility literature, focusing on actors (researchers, publishers, funders). The availability and accessibility of behavioral health services is uneven across populations and conditions, with many unable to access needed care [5]. Identifying the leading researchers, publication venues, and funding sources supports efforts to coalesce this literature. We found the key actors across these areas.

### Publication trends

The trends presented in this bibliometric analysis are consistent with publication trends outside the scope of behavioral health availability and accessibility literature [46]. In this analysis, there has been an increase in publications in the last five years. Fontelo and Liu [47] suggest similar trends with increased literature production across various disciplines and publication types [47]. Due to this broader pattern of publication proliferation, it is not possible to determine if the recent increase in behavioral health service availability/accessibility articles is an indication of increased appetite in this scholarly area. The number of publications can be influenced by changes in promotional expectations at universities, proliferation of journals, increases in collaborative projects, increasing ease of data collection, and the open access movement. Subsequent studies are needed to determine whether the increase is part of general publication inflation or an increase in scholarly interest.

### Leading authors and research teams

Correctly linking articles to authors is challenging. Author naming conventions across journals are not uniform. Some journals list authors’ full names, some only list last name and initials. Authors also may choose to list a middle initial in some papers and not others. Some authors may change their name over the course of their career. Efforts to use a unique author identification number that remains constant for the author regardless of changes to name would greatly improve linkage of articles to authors. One example is the growing availability of adding an Open Researcher and Contributor ID (ORCID) number to article submissions.

Determining the influence of a researcher after articles are linked is also challenging. Various metrics have been proposed and each has its unique challenges [42]. For example, authorship order could be used, but order standards can vary by discipline and journal. In some disciplines, first author is considered the lead; while in others, the author order is organized alphabetically. H-index has largely been used, but changing co-authorship patterns and practices have decreased its validity [48]. Using a multifaceted approach that accounts for number of articles, fractional allocation of authorship order, and citations may prove to be a more accurate measure of influence.

Our study provides a fitting case for the complexity of measuring scholarly productivity. Table 1 provides four different data points: total number of articles, combined weighted counting score, number of first author papers, and number of papers as a co-author. The combined weighted counting score factors in the number of articles, number of authors on the paper, number of first-author papers, and number of papers as a co-author. Sorting that table based on any of the four columns would result in some changes to the order of leading authors. We sorted it by the combined weighted counting score because it allows a more nuanced assessment of contribution to article production. There were some authors with the same count of articles, same count of first-author and co-author articles who had different weighted scores because the total number of authors on their respective papers was different. This underscores the importance of using approaches that can account for more complexity.

The social network authorship analysis shows a concentration of scholarly work among eight groups. The low density of the graph indicated that there are more potential connections than actual connections among the community networks. The high modularity coefficient could be inferred as great strength between existing collaboration relationships. The clustering coefficient suggests a low clustering tendency between the authors. Overall, the statistics of the social network authorship analysis show that collaborations between authors should be encouraged, particularly across the clusters.

Additionally, the network analysis and combined weighted counting score show that while there are many researchers involved with the production of research in this area, a relatively small percentage of those authors would be considered leaders in the field. For instance, of the 140 authors visualized in the network analysis, only 24 (17%) had three or more publications. The remaining 116 authors were connected to research projects in this area but did not appear to have prolonged engagement with the topic as measured by the production of publications. This may point to the multi-disciplinary nature of many publications where, for example, a methodologist whose expertise is agnostic to substantive area may contribute to a paper. In addition, of the 24 top producing authors, 50% published an article in the past five years of the dataset. Researchers can change their research focus over time. Selecting potential collaborators involves knowing what expertise they have and if they have a current appetite for the area.

### Publication venues

The findings from this analysis highlight the effect of differing time horizons. The ranked list of publication venues covering the lifetime of the dataset (1969–2022) differed from the ranked list of journals for the last five years of the dataset (2018–2022). *Psychiatric Services* was the top outlet on both lists demonstrating its long-term and consistent commitment to publishing studies about behavioral health service availability and access. But there were three journals who were among the top 20 lifetime who have not published articles in this area in the last five years. There was also the emergence of three journals who would not have been on the top 20 lifetime list who have published more frequently in the past five years compared to other journals. Knowing both the remote and recent publication past of journals can help researchers best target publication venues for their work.

Interestingly, the three journals added to the top publications list due to their recent publication trends are reputable open access journals. This pattern reflects a larger trend of increasing numbers of open access journals. The proliferation of open access journals has grown exponentially [49]. In less than 10 years the number of open access journals listed in the Directory of Open Access Journals grew from 22 to 16,589 [49]. For government regulators, open access is about allowing the public to freely learn from government-funded work by reducing paywall barriers [50]. In the open access model, the submitting authors pay publications fees rather than readers paying journal subscription fees [50]. While the intent of open access has been to remove barriers, others have commented on the inequities and challenges this system has introduced [51–53]. The amount of available funding to cover open-access fees will be a key determining factor for which journals are viable options for researchers.

Graduate theses and dissertations were the second-highest publication source if all of the data were included (from 1969 to 2022) but has decreased in the last five years. In the past five years there were 3 publications, prior to 2018 there were 10. Ideally, all research, published or unpublished, on the topic would be included in a review to make it highly comprehensive. However, doing so is often unfeasible. Whether to include grey material (e.g., dissertations and theses) is unsettled, and it is important to assess the impact of including grey material [54]. Many reviews do not include studies from this source due to issues such as how well they have been peer-reviewed [54]. One study found many review processes included searching for unpublished sources, but included relatively few [55]. It is possible that including unpublished sources might have little to no effect on results and might generate less conservative estimates [55]. However, some institutions, like Cochrane, have signaled the importance of including unpublished work [56]. Including grey material (e.g., dissertations and theses), when fitting to the research question, has several advantages. They typically do not have page/word limits, so their method sections can capture nuances removed for parsimony in published research. They often include appendices with the full measures used in their study. And, at least in principle, these papers have been reviewed and approved by multi-person expert committees. Graduate theses and dissertations could be important sources for behavioral health services availability and accessibility researchers to include depending on the focus of their study.

### Funders

Given their respective agency missions, it is no surprise that government agencies such as the National Institute on Drug Abuse and the National Institute of Mental Health were the top research funders on the availability and accessibility of behavioral health services. Foundations like the Robert Wood Johnson Foundation were the second top research funders in this space. Notably, 26% of articles did not report funding, a concerning proportion considering bias that may result from funder priorities [57]. Aligned with recommendations from Daou and colleagues in the public health sphere [58], we recommend that journals require authors to submit their funding sources (including non-government funding sources) or to report “no funding” to reduce potential bias in future research on the availability and accessibility of behavioral health services.

### Keywords

Across the 265 articles, 3,376 keywords were used to distinguish the publication content. Each article utilized, on average, 8.7 keywords with 1,555 unique keywords. Almost half of the keywords used were unique and separate from other publications addressing similar ideas. The use of different words to define the same constructs can make it difficult to extract data and information representing similar ideas. Indeed, the most frequent reason for exclusion at the full-text phase was that articles were measuring utilization instead of availability or accessibility. The heterogeneity in how the terms availability and accessibility were defined is hampering the accumulation of knowledge. Additionally, the use of certain terms may change over time [59]. Among the most used key words presented in Table 4, 12% of them haven’t been used in the last five years of the dataset. This could indicate a shift in the profession’s language choice for certain constructs. This further complicates the ability to merge research ideas and create a more feasible process of finding analogous concepts. Consolidating and comparing research would be easier if articles used a common taxonomy of terms and definitions.

### Limitations

The findings of our study should be viewed within the context of its limitations. We have a highly focused sample of articles. Our article pool represents studies that measured the availability or accessibility of behavioral health services quantitatively or using mixed methods. Qualitative-only studies were not included. Further, given our conceptual framework, we did not include articles that used utilization as their proxy for availability or accessibility [4]. We were limited to articles written in English, which would have downstream impacts on all our major outcomes. We only visualized authors with three or more articles and their respective co-authors in our social network analysis [44]. Leading authors were operationalized using the production of articles. While we did use a more sophisticated analysis to account for various facets of article leadership, it may be that a combination of data points (e.g., funded grants) would provide a more nuanced ranking. Furthermore, researchers can shift their research interest over time; the extracted data do not provide an indicator beyond article publication year about researchers’ current activity in this area. Our publication trend analysis uses article count which is susceptible to overall publication inflation, subsequent analysis could use additional data (e.g., overall publication trends) to account for inflation [46]. While we inserted recency indicators with the funders, the extracted data do not indicate if there are funding opportunities currently available in this research area. Each of the findings are subject changes in the future; journals can shift publication focus, funders can move to different priorities, authors can shift to different areas, and areas can refine keywords.

## Conclusion

In conclusion, this bibliometric analysis suggests that research on the availability and accessibility of behavioral health services has risen steadily over the past ten years. Aligned with Tanahashi’s seminal framework, we suggest that researchers distinguish the concepts of “availability” and “accessibility” from “utilization.” The primary funders for the research in this area were government agencies and foundations. There are several networks of researchers doing work in this area. It could be beneficial to increase collaborations among these networks. Given the societal costs of behavioral health disorders, further research should be conducted on methods to improve the availability and accessibility of behavioral health services.

### Supplementary Information

Below is the link to the electronic supplementary material.

**Supplementary file 1:** (DOCX 247 KB)

## Data Availability

Data are available upon request from the corresponding author.

## Code availability

Code is available upon request from the corresponding author.
