# Supplementary material for: Key actors in behavioral health services availability and accessibility research: a scoping review bibliometric analysis
Source: Discov Ment Health. 2024 May 3;4(1):15. doi: 10.1007/s44192-024-00068-3 (PMC11068714; doi:10.1007/s44192-024-00068-3)
Supplement: Supplementary file 1 — (DOCX 247 KB) [file 44192_2024_68_MOESM1_ESM.docx]

**Supplemental File**

# Table S1. EBSCO^1^ search strategy that included CINAHL, Medline, & PsycINFO databases.

| **#** | **Terms** | **Field searched** |
| --- | --- | --- |
| 1 | "mental health" OR "mental illness*" OR "mental disorder*" OR "mental disab*" OR "behavioral health" OR "behavioral disorder" OR "behavioral disturbance"OR "behavioural health" OR "behavioural disorder" OR "behavioural disturbance"OR "serious emotional distress" OR "emotional disorder" OR "psychiatric ill*" OR "emotion* health" OR "internalizing" OR "externalizing" OR "common mental disorder*" OR "mood disorder" OR "affective disorder" OR "psychiatric" OR "psychiatry" | TI, abstract, subject terms |
| 2 | "anxiety" OR agoraphobia OR "panic disorder" OR "phobia*" OR "acrophobia" OR "agoraphobia" OR "claustrophobia" OR "ophidiophobia" OR "school phobia" OR "generalized anxiety disorder" OR "obsessive-compulsive disorder" OR "obsessive compulsive disorder" OR "reactive attachment disorder" OR "separation anxiety disorder" OR "worry" | Title, abstract, subject terms |
| 3 | depress* OR "disruptive mood dysregulation disorder" | Title, abstract, subject terms |
| 4 | adhd OR ADD OR "attention deficit" OR "attenttion-deficit" OR "attention-deficit disorder" OR "attention-deficit hyperactivity disorder" OR "oppositional defiant disorder" OR "conduct disorder" OR "disruptive behavior" | Title, abstract, subject terms |
| 5 | trauma* OR "traum* exposure" OR "post-traumatic stress" OR "posttraumatic stress" OR "acute stress" | Title, abstract, subject terms |
| 6 | "eating disorder*" OR "anorexia nervosa" OR "bulimia" OR "avoidant restrictive food intake disorder" OR "binge eating" OR "picky eating" OR "purging" | Title, abstract, subject terms |
| 7 | "suicide" OR "suicidality" OR "self-injurious" | Title, abstract, subject terms |
| 8 | "bipolar disorder" OR "cyclothymic personality" OR "mania" OR "dysthymic disorder" | Title, abstract, subject terms |
| 9 | "schizophrenia" OR "paranoid" OR "schizophreniform" OR "psychoticism" OR "psychosis" OR "psychotic" | Title, abstract, subject terms |
| 10 | "adjustment disorder" | Title, abstract, subject terms |
| 11 | "personality disorder*" OR "antisocial personality disorder" OR "avoidant personality disorder" OR "borderline personality disorder" OR "dependent personality disorder" OR "histrionic personality disorder" OR "narcissistic personality disorder" OR "obsessive compulsive personality disorder" OR "passive aggressive personality disorder" OR "sadomasochistic personality" OR "schizoid personality disorder" OR "schizotypal personality disorder" | Title, abstract, subject terms |
| 12 | "substance-related disorder*" OR "substance related disorder*" OR "substance use" OR "drug use disorder" OR "substance dependence" OR "substance abuse" OR "substance use" OR "substance misuse" OR "alcohol use" OR "alcohol abuse" OR "alcohol misuse" OR "drug abuse" OR "drug abuse" OR "drug misuse" OR "cannabis abuse" OR "marijuana abuse" OR "alcohol" OR "opiod" OR "tobacco" OR "nicotine" OR "marijuana" OR "cannabis" | Title, abstract, subject terms |
| 13 | "alcoholic hallucinosis" OR "delirium tremens" OR "korsakoffs psychosis" OR "wernickes syndrome" OR "alcoholic psychosis" OR "alcoholic hallucinosis" OR "alcoholism" OR "alcoholic psychosis" OR "alcohol use disorder" OR "alcohol dependence" OR "drug dependence" OR "cannabis dependence" OR "marijuana dependence" OR "tobacco use disorder" OR "nicotine dependence" OR "substance dependence" | Title, abstract, subject terms |
| 14 | S1 OR S2 OR S3 OR S4 OR S5 OR S6 OR S7 OR S8 OR S9 OR S10 OR S11 OR S12 OR S13 | Title, abstract, subject terms |
| 15 | psychotherap* OR counsel* OR "group therapy" OR "group counsel*" OR "one-on-one counseling" OR "family therapy" OR "family counsel*" OR "couple therapy" OR "couple counsel*" | Title, abstract, subject terms |
| 16 | "psycho* service*" OR "psycho* care" OR "psycho* provision" OR "psycho* intervention*" OR "psycho* program*" OR "psycho* project*" OR "psycho* therap*" OR "psycho* treatment*" OR "psycho* prevention*" OR "psycho* promotion*" | Title, abstract, subject terms |
| 17 | "mental health service*" OR "mental health care" OR "mental health provision" OR "mental health intervention*" OR "mental health program*" OR "mental health project*" OR "mental health therap*" OR "mental health treatment*" OR "mental health prevention*" OR "mental health promotion*" | Title, abstract, subject terms |
| 18 | "community mental health service*" OR "community mental health care" OR "community mental health provision" OR "community mental health intervention*" OR "community mental health program*" OR "community mental health project*" OR "community mental health therap*" OR "community mental health treatment*" OR "community mental health prevention*" OR "community mental health promotion*" | Title, abstract, subject terms |
| 19 | "psychiatr* service*" OR "psychiatr* care" OR "psychiatr* provision" OR "psychiatr* intervention*" OR "psychiatr* program*" OR "psychiatr* project*" OR "psychiatr* therap*" OR "psychiatr* treatment*" OR "psychiatr* prevention*" OR "psychiatr* promotion*" | Title, abstract, subject terms |
| 20 | "behavior* health service*" OR "behavior* health care" OR "behavior* health provision" OR "behavior* health intervention*" OR "behavior* health program*" OR "behavior* health project*" OR "behavior* health therap*" OR "behavior* health treatment*" OR "behavior* health prevention*" OR "behavior* health promotion*" | Title, abstract, subject terms |
| 21 | "behaviour* health service*" OR "behaviour* health care" OR "behaviour* health provision" OR "behaviour* health intervention*" OR "behaviour* health program*" OR "behaviour* health project*" OR "behaviour* health therap*" OR "behaviour* health treatment*" OR "behaviour* health prevention*" OR "behaviour* health promotion*" | Title, abstract, subject terms |
| 22 | "substance abuse service*" OR "substance abuse care" OR "substance abuse provision" OR "substance abuse intervention*" OR "substance abuse program*" OR "substance abuse project*" OR "substance abuse therap*" OR "substance abuse treatment*" OR "substance abuse prevention*" OR "substance abuse promotion*" | Title, abstract, subject terms |
| 23 | "addiction* service*" OR "addiction* care" OR "addiction* intervention*" OR "addiction* program*" OR "addiction* project*" OR "addiction* therap*" OR "addiction* treatment*" OR "addiction* prevention*" | Title, abstract, subject terms |
| 24 | "drug abuse service*" OR "drug abuse care" OR "drug abuse intervention*" OR "drug abuse program*" OR "drug abuse project*" OR "drug abuse therap*" OR "drug abuse treatment*" OR "drug abuse prevention*" | Title, abstract, subject terms |
| 25 | "alcohol abuse service*" OR "alcohol abuse care" OR "alcohol abuse intervention*" OR "alcohol abuse program*" OR "alcohol abuse project*" OR "alcohol abuse therap*" OR "alcohol abuse treatment*" OR "alcohol abuse prevention*" | Title, abstract, subject terms |
| 26 | S15 OR S16 OR S17 OR S18 OR S19 OR S20 OR S21 OR S22 OR S23 OR S24 OR S25 | Title, abstract, subject terms |
| 51 | availab* OR access* | Title |
| 57 | "measure" OR "measures" OR "measurement" OR "measurements" OR "instrument" OR "instruments" OR "survey" OR "surveys" OR "questionnaire" OR "questionnaires" OR "scale" OR "scales" OR "self-report" OR "self-reports" OR "self-reported" OR "client-report*" OR "patient-report*" OR "archived data" OR "quantitative" OR "quantitatively" OR "inventory" OR "inventories" OR "rating" OR "ratings" OR "assessment form" OR "assessment forms" OR "evaluation form" OR "evaluation forms" OR "tool" OR "tools" OR "index" OR "indexes" OR "indices" OR "spatial" OR "service map*" | Title, abstract, subject terms |
| 58 | S14 AND S26 AND S51 AND S57 |  |

^1^= This search strategy was translated and ran in Embase and Web of Science Core Collection

# Table S2. Authors, article counts, and combined weighted counting in behavioral health availability accessibility article sample for authors with 2 or more articles.

| Author | Articles | CWC | 1st author | Co author |
| --- | --- | --- | --- | --- |
| Knudsen, H. K. | 7 | 3.60 | 5 | 2 |
| Abraham, A. J. | 7 | 2.56 | 5 | 2 |
| Roman, P. M. | 6 | 1.67 | 0 | 6 |
| Andrews, C. M. | 5 | 1.89 | 2 | 3 |
| Cummings, J. R. | 5 | 1.59 | 3 | 2 |
| West, J. C. | 5 | 1.04 | 2 | 3 |
| Fortney, J. C. | 5 | 1.00 | 2 | 3 |
| Druss, B. G. | 4 | 0.79 | 0 | 4 |
| Rae, D. S. | 4 | 0.54 | 0 | 4 |
| Wen, H. | 3 | 1.03 | 2 | 1 |
| Ducharme, L. J. | 3 | 1.00 | 1 | 2 |
| McCarthy, J. F. | 3 | 0.82 | 2 | 1 |
| Yarbrough, C. R. | 3 | 0.76 | 0 | 3 |
| Guerrero, E. G. | 3 | 0.72 | 1 | 2 |
| McBain, R. | 3 | 0.65 | 1 | 2 |
| Pyne, J. M. | 3 | 0.64 | 2 | 1 |
| Wilk, J. E. | 3 | 0.61 | 1 | 2 |
| Kilbourne, A. M. | 3 | 0.59 | 0 | 3 |
| Busch, S. H. | 3 | 0.56 | 0 | 3 |
| Rieckmann, T. | 3 | 0.56 | 0 | 3 |
| Regier, D. A. | 3 | 0.43 | 0 | 3 |
| Saloner, B. | 3 | 0.39 | 0 | 3 |
| Fischer, E. P. | 3 | 0.26 | 0 | 3 |
| Kugelmass, H. | 2 | 2.00 | 2 | 0 |
| Myers, B. | 2 | 1.60 | 2 | 0 |
| Shover, C. L. | 2 | 1.60 | 2 | 0 |
| Rosenberg, H. | 2 | 1.07 | 2 | 0 |
| Williams, N. D. | 2 | 0.96 | 2 | 0 |
| West, S. L. | 2 | 0.93 | 2 | 0 |
| Rocks, S. | 2 | 0.83 | 2 | 0 |
| Brooks, R. G. | 2 | 0.80 | 2 | 0 |
| Cronin, T. J. | 2 | 0.80 | 2 | 0 |
| Gonzales, G. | 2 | 0.80 | 0 | 2 |
| Lora, A. | 2 | 0.80 | 2 | 0 |
| Schlesinger, M. | 2 | 0.80 | 1 | 1 |
| Cantor, J. H. | 2 | 0.76 | 2 | 0 |
| Creedon, T. B. | 2 | 0.71 | 1 | 1 |
| Huhn, A. S. | 2 | 0.67 | 2 | 0 |
| Alexander, J. A. | 2 | 0.67 | 0 | 2 |
| Snowden, L. R. | 2 | 0.67 | 0 | 2 |
| Shah, A. | 2 | 0.63 | 1 | 1 |
| Weinzimmer, L. G. | 2 | 0.56 | 1 | 1 |
| Dalstrom, M. D. | 2 | 0.56 | 1 | 1 |
| Fish, J. N. | 2 | 0.56 | 0 | 2 |
| Harris, S. J. | 2 | 0.56 | 1 | 1 |
| Cifu, D. X. | 2 | 0.53 | 0 | 2 |
| Graham, C. W. | 2 | 0.53 | 0 | 2 |
| Friedmann, P. D. | 2 | 0.53 | 1 | 1 |
| Zeber, J. E. | 2 | 0.52 | 1 | 1 |
| Jayawardhana, J. | 2 | 0.47 | 0 | 2 |
| Mościcki, E. K. | 2 | 0.43 | 1 | 1 |
| Krawczyk, N. | 2 | 0.43 | 1 | 1 |
| Solomon, K. T. | 2 | 0.43 | 1 | 1 |
| Bagwell-Adams, G. | 2 | 0.43 | 0 | 2 |
| Fazel, M. | 2 | 0.43 | 0 | 2 |
| Tsiachristas, A. | 2 | 0.43 | 0 | 2 |
| Olin, S. S. | 2 | 0.40 | 1 | 1 |
| Clawson, A. | 2 | 0.40 | 0 | 2 |
| Halford, W. K. | 2 | 0.40 | 0 | 2 |
| Lyons, A. | 2 | 0.40 | 0 | 2 |
| Marcus, S. C. | 2 | 0.40 | 0 | 2 |
| Menachemi, N. | 2 | 0.40 | 0 | 2 |
| Pepping, C. A. | 2 | 0.40 | 0 | 2 |
| Salvador-Carulla, L. | 2 | 0.39 | 1 | 1 |
| Kuramoto-Crawford, J. | 2 | 0.38 | 0 | 2 |
| Wallace, N. T. | 2 | 0.38 | 0 | 2 |
| Foulger, R. | 2 | 0.36 | 0 | 2 |
| Klein, C. J. | 2 | 0.36 | 0 | 2 |
| Kofner, A. | 2 | 0.36 | 0 | 2 |
| Ruud, T. | 2 | 0.36 | 0 | 2 |
| Stein, B. D. | 2 | 0.36 | 0 | 2 |
| Strain, E. C. | 2 | 0.32 | 0 | 2 |
| Morris, J. | 2 | 0.29 | 0 | 2 |
| Pincus, H. A. | 2 | 0.29 | 0 | 2 |
| Garnick, D. W. | 2 | 0.27 | 0 | 2 |
| Hobelmann, J. G. | 2 | 0.27 | 0 | 2 |
| Horgan, C. M. | 2 | 0.27 | 0 | 2 |
| Oyler, G. A. | 2 | 0.27 | 0 | 2 |
| Reif, S. | 2 | 0.27 | 0 | 2 |
| Stewart, M. T. | 2 | 0.27 | 0 | 2 |
| Rubio-Stipec, M. | 2 | 0.27 | 0 | 2 |
| Valenstein, M. | 2 | 0.26 | 0 | 2 |
| Hawrilenko, M. | 2 | 0.24 | 0 | 2 |
| Mojtabai, R. | 2 | 0.23 | 0 | 2 |
| Baggett, T. P. | 2 | 0.23 | 0 | 2 |
| Kerr, T. | 2 | 0.23 | 0 | 2 |
| Stuart, E. A. | 2 | 0.23 | 0 | 2 |
| Wood, E. | 2 | 0.23 | 0 | 2 |
| Duffy, F. F. | 2 | 0.21 | 0 | 2 |
| Priebe, S. | 2 | 0.21 | 0 | 2 |
| Hoagwood, K. E. | 2 | 0.20 | 0 | 2 |
| Horwitz, S. M. | 2 | 0.20 | 0 | 2 |
| O'Connor, B. C. | 2 | 0.20 | 0 | 2 |
| Storfer-Isser, A. | 2 | 0.20 | 0 | 2 |
| Whitmyre, E. D. | 2 | 0.20 | 0 | 2 |
| Kelly, P. A. | 2 | 0.16 | 0 | 2 |
| Koenig, C. J. | 2 | 0.16 | 0 | 2 |
| Miller, C. J. | 2 | 0.16 | 0 | 2 |
| Wright, P. | 2 | 0.16 | 0 | 2 |
| Zamora, K. | 2 | 0.16 | 0 | 2 |

CWC = combined weighted counting

# Table S3. Publication sources and number of articles per source for behavioral health service availability and accessibility articles.

| **Journal** | **Freq.** |
| --- | --- |
| Psychiatric Services | 22 |
| Graduate Theses | 13 |
| Health Services Research | 7 |
| Drug & Alcohol Dependence | 6 |
| Health Affairs | 5 |
| Journal of Substance Abuse Treatment | 5 |
| The Journal of Behavioral Health Services & Research | 5 |
| Administration and Policy in Mental Health and Mental Health Services Research | 4 |
| American Journal of Public Health | 4 |
| Military Medicine | 4 |
| Psychological Services | 4 |
| BMC Health Services Research | 3 |
| BMC Psychiatry | 3 |
| Children and Youth Services Review | 3 |
| JAMA Psychiatry | 3 |
| Journal of General Internal Medicine | 3 |
| Journal of Rural Health | 3 |
| Medical Care | 3 |
| Plos One | 3 |
| Substance Use & Misuse | 3 |
| Alcoholism Treatment Quarterly | 2 |
| American Annals of the Deaf | 2 |
| Archives of Internal Medicine | 2 |
| Archives of Pediatrics & Adolescent Medicine | 2 |
| Australasian Psychiatry | 2 |
| Australian Psychologist | 2 |
| Behaviour Research and Therapy | 2 |
| BMJ Open | 2 |
| BMJ Open Quality | 2 |
| Bulletin of the World Health Organization | 2 |
| Community Mental Health Journal | 2 |
| Drugs: Education, Prevention & Policy | 2 |
| General Hospital Psychiatry | 2 |
| Health & Place | 2 |
| JAMA Network Open | 2 |
| Journal of Adolescent Health | 2 |
| Journal of Health Care for the Poor & Underserved | 2 |
| Pediatrics | 2 |
| The Australian and New Zealand Journal of Psychiatry | 2 |
| Academic Pediatrics | 1 |
| Academic Psychiatry | 1 |
| Addiction | 1 |
| Addictive Behaviors | 1 |
| Addictive Disorders & Their Treatment | 1 |
| Aging & Mental Health | 1 |
| Alkoholizm I Narkomania-Alchoholism and Drug Addition | 1 |
| American Journal of Emergency Medicine | 1 |
| American Journal of Physical Medicine & Rehabilitation | 1 |
| Annals of Emergency Medicine | 1 |
| Annals of Family Medicine | 1 |
| Archives of Gerontology and Geriatrics | 1 |
| Australian Journal of Rural Health | 1 |
| BMC Medical Informatics And Decision Making | 1 |
| BMC Palliative Care | 1 |
| Brazilian Journal of Physical Therapy | 1 |
| Canadian Journal of Psychiatry | 1 |
| Canadian Journal Of Public Health | 1 |
| Canadian Psychology | 1 |
| Children's Health Care | 1 |
| Digestive Diseases & Sciences | 1 |
| Environment and Planning B-Planning & Design | 1 |
| Epidemiology and Psychiatric Sciences | 1 |
| European Journal of Health Economics | 1 |
| European Journal of Public Health | 1 |
| Family Practice | 1 |
| Health & Social Care in the Community | 1 |
| Health Equity | 1 |
| Health Informatics Journal | 1 |
| Higher Education | 1 |
| Hospital Topics | 1 |
| International Journal of Environmental Research and Public Health | 1 |
| International Journal of Geriatric Psychiatry | 1 |
| International Journal of Law and Psychiatry | 1 |
| International Journal of Lean Six Sigma | 1 |
| International Journal of Mental Health Systems | 1 |
| International Journal of Nursing Practice | 1 |
| International Journal of Social Psychiatry | 1 |
| International Journal of Transgender Health | 1 |
| Japanese Journal Of Clinical Oncology | 1 |
| JGIM: Journal of General Internal Medicine | 1 |
| JMIR Mental Health | 1 |
| Journal for Healthcare Quality | 1 |
| Journal of Addiction Medicine | 1 |
| Journal of Affective Disorders | 1 |
| Journal of Aging and Health | 1 |
| Journal of Alcohol and Drug Education | 1 |
| Journal of Ambulatory Care Management | 1 |
| Journal of Applied Gerontology | 1 |
| Journal of Cancer Survivorship | 1 |
| Journal of Child & Adolescent Substance Abuse | 1 |
| Journal of Child Health Care | 1 |
| Journal of Clinical Psychology | 1 |
| Journal of Clinical Sport Psychology | 1 |
| Journal of Correctional Health Care | 1 |
| Journal of Drug Issues | 1 |
| Journal of Forensic Psychiatry & Psychology | 1 |
| Journal of Gay & Lesbian Social Services | 1 |
| Journal Of General Internal Medicine | 1 |
| Journal of Health and Social Behavior | 1 |
| Journal of Homosexuality | 1 |
| Journal of Medical Education | 1 |
| Journal of Nervous and Mental Disease | 1 |
| Journal of Paediatrics and Child Health | 1 |
| Journal of Pain Research | 1 |
| Journal of Pediatric Surgery | 1 |
| Journal of Prison & Jail Health | 1 |
| Journal of Psychiatric Practice | 1 |
| Journal of Rural Mental Health | 1 |
| Journal of School Health | 1 |
| Journal of Social Work in Disability & Rehabilitation | 1 |
| Journal of Social Work Practice in the Addictions | 1 |
| Journal of Studies on Alcohol and Drugs | 1 |
| Journal of studies on alcohol and drugs | 1 |
| Journal of Surgical Education | 1 |
| Journal of the American Academy of Child & Adolescent Psychiatry | 1 |
| Journal of the American Geriatrics Society | 1 |
| Journal of the Canadian Academy of Child and Adolescent Psychiatry | 1 |
| Journal of Urban Health | 1 |
| Learning Disability Practice | 1 |
| LGBT Health | 1 |
| Maternal & Child Health Journal | 1 |
| Mayo Clinic Proceedings | 1 |
| Mental Health and Social Inclusion | 1 |
| Mental Health Aspects of Developmental Disabilities | 1 |
| Mental Health Practice | 1 |
| Mental Health Review Journal | 1 |
| MMWR: Morbidity & Mortality Weekly Report | 1 |
| Nonprofit and Voluntary Sector Quarterly | 1 |
| Oncologist | 1 |
| Plos Medicine | 1 |
| Population Research and Policy Review | 1 |
| Psychiatric Bulletin | 1 |
| Psychiatric Rehabilitation Journal | 1 |
| Psychology of Addictive Behaviors | 1 |
| Public Health Nursing | 1 |
| Public Health Reports | 1 |
| Revista Panamericana de Salud Publica | 1 |
| Rural & Remote Health | 1 |
| Scandinavian Journal of Urology and Nephrology | 1 |
| Social Psychiatry and Psychiatric Epidemiology | 1 |
| Social Science & Medicine | 1 |
| Social Work | 1 |
| South African Psychiatry Review | 1 |
| Spatial and Spatio-Temporal Epidemiology | 1 |
| Substance Abuse Treatment, Prevention, And Policy | 1 |
| Substance Abuse: Research and Treatment | 1 |
| Suicide & Life-Threatening Behavior | 1 |
| Supportive Care in Cancer | 1 |
| The American Journal of Drug and Alcohol Abuse | 1 |
| The American Journal on Addictions | 1 |
| The CBHSQ Report | 1 |
| The International Journal on Drug Policy | 1 |
| The Journal of Clinical Psychiatry | 1 |
| The Journal of Mental Health Policy and Economics | 1 |
| Topics in Spinal Cord Injury Rehabilitation | 1 |
| Transportation Research Part D-Transport and Environment | 1 |
| Urban Affairs Review | 1 |
| Wisconsin Medical Journal | 1 |
| Women and Birth | 1 |
| Women's Health Issues | 1 |

# Table S4. Funding sources and number of behavioral health service availability or accessibility articles supported by that funding source.

| **Funding source** | **Freq.** |
| --- | --- |
| not reported | 100 |
| National Institute on Drug Abuse | 28 |
| National Institute of Mental Health | 16 |
| none | 15 |
| Veterans Administration | 13 |
| Agency for Healthcare Research and Quality | 8 |
| National Institute of Child Health and Human Development | 5 |
| National Institute on Alcohol Abuse and Alcoholism | 5 |
| The Robert Wood Johnson Foundation | 5 |
| American Psychiatric Association Foundation | 3 |
| Canadian Institutes of Health Research | 3 |
| Health Resources and Services Administration | 3 |
| Michael Smith Foundation | 3 |
| National Center for Advancing Translational Sciences | 3 |
| National Institute on Minority Health and Disparities | 3 |
| Patient Centered Outcomes Research Institute | 3 |
| Center for Substance Abuse Treatment | 2 |
| Centers for Medicare and Medicaid Services | 2 |
| Clinical Commissioning Groups in Oxfordshire and Buckinghamshire | 2 |
| Janssen | 2 |
| National Heart, Lung, and Blood Institute | 2 |
| National Institute for Health Research | 2 |
| National Institute on Aging | 2 |
| National Institute on Disability and Rehabilitation Research | 2 |
| National Institutes of Allergy and Infectious Diseases | 2 |
| NIHR Collaboration for Leadership in Applied Health Research | 2 |
| Princeton Center for Health and Wellbeing | 2 |
| Swiss State Secretariat for Education, Research, and Innovation | 2 |
| Wu Tsai Neurosciences Institute | 2 |
| Aaron Diamon Foundation | 1 |
| Agence Nationale de la Recheche | 1 |
| Alzheimer's Association | 1 |
| Alzheimer's Society | 1 |
| American Society for Radiation Oncology | 1 |
| Andalusian Research Plan | 1 |
| Ashley Addiction Treatment | 1 |
| Association for Behavioral Health and Wellness | 1 |
| Astellas | 1 |
| Augmenix | 1 |
| Australian Government Department of Health and Aging | 1 |
| Australian Government Research Training Program Scholarship | 1 |
| Australian National Health and Medical Research Council Career Development Fellowship | 1 |
| Australian Research Council Future Fellowship | 1 |
| Autistica | 1 |
| Baum Charitable Foundation | 1 |
| Bayer | 1 |
| beyondblue: The National Depression Initiative | 1 |
| Bloomberg Philanthropies | 1 |
| Blue Earth | 1 |
| BlueCross BlueShield Association | 1 |
| Boston Scientific | 1 |
| Brain and Mind Centre University of Sydney | 1 |
| Brazil and Fundacao de Pesquisa de Minas Gerais Brazil | 1 |
| California Community Foundation | 1 |
| Canada Research Chair in Global Sexual Health and HIV/AIDS | 1 |
| Cancer Foundation | 1 |
| Care East Midlands | 1 |
| Care Oxford at Oxford Health NHS Foundation Trust | 1 |
| Center for Mental Health Services | 1 |
| Center for Rural Health Research and Policy of the Florida State University College of Medicine | 1 |
| Center for Studying Health System Change | 1 |
| Centers for Disease Control and Prevention | 1 |
| Centre for Health Economics University of York | 1 |
| Children's Health Insurance Program Reauthorization Act | 1 |
| City of University of New York | 1 |
| Colorado Child Health Foundation | 1 |
| Commonwealth Fund | 1 |
| Commonwealth of Australia | 1 |
| Connecticut Office of the Healthare Advocate | 1 |
| ConNetica Consultying | 1 |
| Conselho Nacional de Pesquisa Brazil | 1 |
| Coordenacao de Aperfeicoamento de Pessoal de Nivel Superior | 1 |
| Cota Healthcare | 1 |
| Dendreon | 1 |
| Department of Health and Human Services | 1 |
| Department of Health’s National Research Programme on Forensic Mental Health R&D | 1 |
| DG-Sanco | 1 |
| Economic and Social Research Council | 1 |
| European Commission through the Horizon 2020 Framework | 1 |
| Faculty of Health Sciences University of Sydney | 1 |
| Fahs-Beck Fund | 1 |
| FEMA | 1 |
| Ferring | 1 |
| Florida Department of Health | 1 |
| Fogarty International Center | 1 |
| Food and Drug Administration | 1 |
| Ford Foundation | 1 |
| Genome Dx | 1 |
| German Federal Ministry of Health | 1 |
| Gilead Sciences Foundation | 1 |
| Graham Boeckh Foundation | 1 |
| Harmonist Project | 1 |
| Health Education East Midlands | 1 |
| Health Equity Research Center Washington University | 1 |
| HHS Office of the Assistant Secretary for Planning and Evaluation | 1 |
| Inge Wakehurst Trust | 1 |
| Institut Nationald’Excellence en Santé et en Services Sociaux du Québec | 1 |
| Irving Institute for Clinical and Translational Research at Columbia University | 1 |
| Japanese Ministry of Health, Labour and Welfare | 1 |
| John D. and Catherine T. MacArthur Foundation | 1 |
| Johnson & Johnson | 1 |
| Liberty Community Services | 1 |
| Lundbeck | 1 |
| MacAIDS | 1 |
| Marie Curie | 1 |
| Maryland Department of Health, Behavioral Health Administration | 1 |
| McNerney Grant | 1 |
| Medical Research Council | 1 |
| Medical University of South Carolina (MUSC) | 1 |
| Medtronic Inc. | 1 |
| Mental Health Therapeutics CERT at Rutgers | 1 |
| MSD | 1 |
| Murdoch Children’s Research Institute | 1 |
| National Cancer Institute | 1 |
| National Center for PTSD | 1 |
| National Center for Research Resources | 1 |
| National Health and Medical Research Council | 1 |
| National Health and Medical Research Council Early Career Fellowship | 1 |
| National Institute of Diabetes and Digestive Kidney Diseases | 1 |
| National Institutes of Health | 1 |
| National Library of Medicine | 1 |
| National Research Service Award | 1 |
| Network of Primary Care Research in Spain | 1 |
| North Carolina Governor’s Advocacy Council for Persons with Disabilities | 1 |
| Northern Norway Regional Health Authority | 1 |
| Northwest Arkansas Rape Crisis. Inc | 1 |
| Norwegian Knowledge Centre for the Health Services | 1 |
| NSW Family and Community Services, Ageing, Disability, and Home Care Western Region | 1 |
| Office of Rural Health Policy | 1 |
| Partnership for Women's and Children's Health | 1 |
| Portuguese Foundation for Science and Technology | 1 |
| Princeton Center for the Study of Social Organization | 1 |
| Princeton Department of Sociology | 1 |
| Prostate Cancer Foundation | 1 |
| Proyecto FONDECYT | 1 |
| Proyecto Universidad del Desar- rollo in Chile | 1 |
| Reckitt Benckiser Pharmaceutical, Inc | 1 |
| Regional Socio-Economic Development Institute of Canada Fund | 1 |
| Research and Development Office of Oxleas NHS Trust | 1 |
| Rose Community Foundation | 1 |
| Sarepta Therapeutics | 1 |
| SmartState South Carolina Centers of Economic Excellence | 1 |
| South African Department of Social Development | 1 |
| Spanish International Cooperation Agency | 1 |
| St Paul’s Foundation | 1 |
| State Government of Victoria | 1 |
| State Government of Victoria Mental Illness Research Fund | 1 |
| State Government of Victoria’s Operational Infra-structure Support Program | 1 |
| Stockholm Cancer Foundation | 1 |
| Substance Abuse and Mental Health Services Administration | 1 |
| Substance Abuse and Mental Health Services Agency | 1 |
| Swedish Cancer Society | 1 |
| The Carnegie Corporation | 1 |
| The Curtis Center of the University of Michigan | 1 |
| The Duke Endowment | 1 |
| The Hellen Keller National Center for Deaf-Blind Youths and Adults | 1 |
| The Helmsley Charitable Trust | 1 |
| The Hunger and Homelessness Project at Yale School of Medicine | 1 |
| The Leukemia & Lymphoma Society | 1 |
| The Pierre Elliott Trudeau Foundation | 1 |
| The Thrasher Research Fund | 1 |
| UCB Biogen | 1 |
| United Hospital Fund | 1 |
| University College London Hospitals NIHR Biomedical Research Centre | 1 |
| University of Alabama at Birmingham School of Medicine | 1 |
| University of British Columbia Okanagan’s Eminence Program | 1 |
| University of California Berkeley | 1 |
| University of Glasgow | 1 |
| University of Melbourne Faculty of Medicine, Dentistry and Health Sciences Research Fellowship | 1 |
| Walter Reed Army Institute of Research | 1 |
| Weill Cornell Medical College | 1 |
| Western Australia Mental Health Commission | 1 |
| Western Australia Primary Health Alliance | 1 |
| Western NSW Primary Health Network | 1 |
| Yale University | 1 |
| Yale-New Haven Hospital Medical Staff Fund | 1 |
| York University Canada | 1 |

# Table S5. Key words from behavioral health services availability and accessibility articles that were used two or more times.

| **Article keywords** | **Freq.** |
| --- | --- |
| human | 86 |
| humans | 73 |
| female | 67 |
| health services accessibility | 63 |
| male | 63 |
| united states | 61 |
| adult | 55 |
| mental health services | 45 |
| mental health | 35 |
| surveys | 27 |
| health services accessibility/statistics & numerical data | 26 |
| descriptive statistics | 25 |
| funding source | 25 |
| medicaid | 24 |
| adolescence | 22 |
| questionnaires | 22 |
| aged | 21 |
| child | 21 |
| adolescent | 18 |
| mental health services/statistics & numerical data | 17 |
| middle aged | 17 |
| chi square test | 16 |
| surveys and questionnaires | 15 |
| data analysis software | 14 |
| interviews | 14 |
| young adult | 14 |
| cross sectional studies | 13 |
| confidence intervals | 12 |
| health care surveys | 12 |
| middle age | 12 |
| access to care | 11 |
| article | 11 |
| depression | 11 |
| health services needs and demand | 11 |
| health services research | 11 |
| logistic regression | 11 |
| mental disorders | 11 |
| odds ratio | 11 |
| substance abuse | 11 |
| cross-sectional studies | 10 |
| p-value | 10 |
| socioeconomic factors | 10 |
| buprenorphine | 9 |
| health care access | 9 |
| healthcare disparities | 9 |
| insurance coverage | 9 |
| outpatients | 9 |
| quality of health care | 9 |
| rural areas | 9 |
| access | 8 |
| drug abuse | 8 |
| health services accessibility -- statistics and numerical data | 8 |
| urban areas | 8 |
| veterans | 8 |
| waiting lists | 8 |
| attitude of health personnel | 7 |
| child, preschool | 7 |
| health care delivery | 7 |
| mental disorders/therapy | 7 |
| mental health services -- utilization | 7 |
| mental health services/supply & distribution | 7 |
| multivariate analysis | 7 |
| none listed | 7 |
| primary health care | 7 |
| prospective studies | 7 |
| referral and consultation | 7 |
| scales | 7 |
| substance abuse treatment centers/statistics & numerical data | 7 |
| treatment | 7 |
| anxiety | 6 |
| blacks | 6 |
| community mental health services | 6 |
| counseling | 6 |
| health status | 6 |
| infant | 6 |
| program evaluation | 6 |
| regression | 6 |
| substance abuse treatment centers/organization & administration | 6 |
| substance use treatment | 6 |
| substance-related disorders/therapy | 6 |
| survey research | 6 |
| united states/epidemiology | 6 |
| aged, 80 and over | 5 |
| australia | 5 |
| california | 5 |
| canada | 5 |
| comorbidity | 5 |
| comparative studies | 5 |
| drug therapy | 5 |
| health care costs | 5 |
| health care utilization | 5 |
| health insurance | 5 |
| health survey | 5 |
| medicare | 5 |
| mental disorders/epidemiology | 5 |
| mental health care | 5 |
| opioid use disorder | 5 |
| pearson's correlation coefficient | 5 |
| random sample | 5 |
| self report | 5 |
| sex factors | 5 |
| stigma | 5 |
| substance use disorders | 5 |
| substance use disorders -- therapy | 5 |
| substance use rehabilitation programs | 5 |
| substance-related disorders/rehabilitation | 5 |
| accessibility | 4 |
| age factors | 4 |
| caregivers | 4 |
| community mental health | 4 |
| crisis intervention | 4 |
| cross-sectional study | 4 |
| data collection | 4 |
| ethnic groups | 4 |
| exploratory research | 4 |
| geographic information systems | 4 |
| health care sciences & services | 4 |
| health service | 4 |
| health services | 4 |
| health services accessibility/economics | 4 |
| health services accessibility/organization & administration | 4 |
| insurance, health | 4 |
| logistic models | 4 |
| major clinical study | 4 |
| mental disorders -- therapy | 4 |
| mental health services -- statistics and numerical data | 4 |
| mental-health-services | 4 |
| methadone | 4 |
| multicenter studies | 4 |
| outcomes (health care) | 4 |
| patient satisfaction | 4 |
| post hoc analysis | 4 |
| pregnancy | 4 |
| prevalence | 4 |
| psychiatry | 4 |
| psychology | 4 |
| psychotherapy | 4 |
| quality improvement | 4 |
| race factors | 4 |
| regression analysis | 4 |
| specialization | 4 |
| substance abuse -- therapy | 4 |
| substance abuse treatment centers/economics | 4 |
| telehealth | 4 |
| telephone | 4 |
| united kingdom | 4 |
| united states department of veterans affairs | 4 |
| validation studies | 4 |
| alcohol abuse | 3 |
| appointments and schedules | 3 |
| architectural accessibility | 3 |
| attitudes | 3 |
| barriers | 3 |
| community mental health services -- utilization | 3 |
| continuity of patient care | 3 |
| controlled study | 3 |
| correlation coefficient | 3 |
| covid-19 | 3 |
| disabled | 3 |
| disparities | 3 |
| england | 3 |
| europe | 3 |
| evaluation research | 3 |
| florida | 3 |
| follow up | 3 |
| geographic factors | 3 |
| geography | 3 |
| health care | 3 |
| health facilities | 3 |
| health policy | 3 |
| health services accessibility -- evaluation | 3 |
| health services accessibility/legislation & jurisprudence | 3 |
| health services accessibility/trends | 3 |
| health surveys | 3 |
| hispanics | 3 |
| infant, newborn | 3 |
| insurance coverage/statistics & numerical data | 3 |
| insurance, health -- statistics and numerical data | 3 |
| medically uninsured | 3 |
| medications for opioid use disorder | 3 |
| mental disorders/diagnosis | 3 |
| mental health access | 3 |
| mental health services -- administration | 3 |
| mental health services/economics | 3 |
| mental health services/standards | 3 |
| minority groups | 3 |
| naltrexone | 3 |
| needs assessment | 3 |
| neoplasms/psychology | 3 |
| opioid-related disorders/drug therapy | 3 |
| outpatient | 3 |
| patient acceptance of health care/statistics & numerical data | 3 |
| patient protection and affordable care act | 3 |
| pilot studies | 3 |
| quality of care | 3 |
| racial disparities | 3 |
| rural environments | 3 |
| rural population | 3 |
| screening | 3 |
| severity of illness index | 3 |
| smoking cessation | 3 |
| social stigma | 3 |
| social support | 3 |
| spatial analysis | 3 |
| special populations | 3 |
| substance abuse treatment centers | 3 |
| substance use | 3 |
| substance use disorders -- drug therapy | 3 |
| substance-related disorders/economics | 3 |
| suicide | 3 |
| summated rating scaling | 3 |
| support, psychosocial | 3 |
| telemedicine | 3 |
| thematic analysis | 3 |
| time factors | 3 |
| treatment facilities | 3 |
| t-tests | 3 |
| urban population | 3 |
| washington | 3 |
| world health organization | 3 |
| addiction | 2 |
| adolescent health services | 2 |
| affordable care act | 2 |
| african americans | 2 |
| alcohol use disorder | 2 |
| alcoholism -- therapy | 2 |
| ambulatory care | 2 |
| ambulatory care facilities | 2 |
| analysis of variance | 2 |
| attitude | 2 |
| attitude of health personnel -- evaluation | 2 |
| attitude to health | 2 |
| availability | 2 |
| british columbia | 2 |
| buprenorphine -- therapeutic use | 2 |
| business & economics | 2 |
| care | 2 |
| case management | 2 |
| catchment area (health) | 2 |
| child health services | 2 |
| chronic disease | 2 |
| clinical assessment tools | 2 |
| coding | 2 |
| coefficient alpha | 2 |
| colorado | 2 |
| community health centers | 2 |
| conceptual framework | 2 |
| continuous quality improvement | 2 |
| coronavirus disease 2019 | 2 |
| correctional institutions | 2 |
| countries | 2 |
| cultural competence | 2 |
| dementia | 2 |
| demography | 2 |
| developing countries/economics | 2 |
| diagnosis, dual (psychiatry) | 2 |
| disorders | 2 |
| drug dependence treatment | 2 |
| dsm | 2 |
| educational status | 2 |
| emergency care | 2 |
| emergency service | 2 |
| epidemic | 2 |
| european union | 2 |
| facilities | 2 |
| factor analysis, statistical | 2 |
| financial support | 2 |
| financing, government | 2 |
| fisher's exact test | 2 |
| focus groups | 2 |
| foster home care | 2 |
| gay | 2 |
| gender-differences | 2 |
| general practice | 2 |
| health | 2 |
| health care delivery, integrated | 2 |
| health care psychology | 2 |
| health care quality | 2 |
| health care seeking behavior | 2 |
| health care services | 2 |
| health center | 2 |
| health economics | 2 |
| health literacy | 2 |
| health services accessibility -- administration | 2 |
| health services needs and demand/statistics & numerical data | 2 |
| health services/statistics & numerical data | 2 |
| health status disparities | 2 |
| help-seeking | 2 |
| homeless persons | 2 |
| hospitalization | 2 |
| impact | 2 |
| income | 2 |
| insurance | 2 |
| insurance carriers | 2 |
| insurance coverage -- statistics and numerical data | 2 |
| insurance, health/statistics & numerical data | 2 |
| interdisciplinary treatment approach | 2 |
| internal consistency | 2 |
| internet access | 2 |
| japan | 2 |
| kruskal-wallis test | 2 |
| lgbtq | 2 |
| longitudinal studies | 2 |
| major depression | 2 |
| managed care programs/trends | 2 |
| management | 2 |
| medicaid -- statistics and numerical data | 2 |
| medicaid/statistics & numerical data | 2 |
| medical information | 2 |
| medicine | 2 |
| mental disease | 2 |
| mental disorders -- epidemiology | 2 |
| mental disorders -- rehabilitation | 2 |
| mental disorders, chronic | 2 |
| mental health disparities | 2 |
| mental health service | 2 |
| mental health services -- standards | 2 |
| mental illness | 2 |
| michigan | 2 |
| models, theoretical | 2 |
| multiple logistic regression | 2 |
| multiple regression | 2 |
| native americans | 2 |
| nursing | 2 |
| ohio | 2 |
| older adults | 2 |
| outcome and process assessment (health care) | 2 |
| outcome assessment | 2 |
| outpatient care | 2 |
| outpatient service | 2 |
| pandemic | 2 |
| pdsa | 2 |
| pediatric care | 2 |
| pharmacotherapy | 2 |
| physician-patient relations | 2 |
| physicians | 2 |
| physicians/supply & distribution | 2 |
| population | 2 |
| poverty | 2 |
| primary care | 2 |
| primary care providers | 2 |
| primary medical care | 2 |
| priority journal | 2 |
| privacy | 2 |
| private health insurance | 2 |
| private sector | 2 |
| probability | 2 |
| professional-patient relations | 2 |
| psychiatric care | 2 |
| psychiatric hospitals | 2 |
| psychiatrists | 2 |
| psychological tests | 2 |
| psychological treatment | 2 |
| psychosis | 2 |
| psychotherapists | 2 |
| public health | 2 |
| public, environmental & occupational health | 2 |
| qualitative studies | 2 |
| quality | 2 |
| quality of life | 2 |
| questionnaire | 2 |
| racial and ethnic differences | 2 |
| record review | 2 |
| referral and consultation -- statistics and numerical data | 2 |
| referral and consultation/organization & administration | 2 |
| referral and consultation/statistics & numerical data | 2 |
| rehabilitation | 2 |
| retrospective design | 2 |
| risk | 2 |
| risk factors | 2 |
| rural | 2 |
| rural health services/supply & distribution | 2 |
| rural mental health | 2 |
| schizophrenia | 2 |
| schizophrenia/therapy | 2 |
| schools, elementary | 2 |
| sequelae | 2 |
| service delivery | 2 |
| sign language | 2 |
| social determinants of health | 2 |
| social work | 2 |
| social work/statistics & numerical data | 2 |
| spatial accessibility | 2 |
| stratified random sample | 2 |
| stress, psychological | 2 |
| substance abuse treatment | 2 |
| substance abuse treatment centers/supply & distribution | 2 |
| substance use rehabilitation programs -- statistics and numerical data | 2 |
| substance-related disorders | 2 |
| substance-related disorders/epidemiology | 2 |
| substance-related disorders/psychology | 2 |
| suicidal ideation | 2 |
| suicide/prevention & control | 2 |
| switzerland | 2 |
| transgender | 2 |
| treatment access | 2 |
| treatment outcomes | 2 |
| treatment services | 2 |
| trends | 2 |
| urban environments | 2 |
| validation | 2 |
| variable | 2 |
| vulnerable populations | 2 |
| whites | 2 |
| women | 2 |
| workforce | 2 |

# **Table S6. PRISMA-ScR checklist**

**Preferred Reporting Items for Systematic reviews and Meta-Analyses extension for Scoping Reviews (PRISMA-ScR) Checklist**

| **SECTION** | **ITEM** | **PRISMA-ScR CHECKLIST ITEM** | **REPORTED ON PAGE #** |
| --- | --- | --- | --- |
| **TITLE** | | | |
| Title | 1 | Identify the report as a scoping review. | Title pg |
| **ABSTRACT** | | | |
| Structured summary | 2 | Provide a structured summary that includes (as applicable): background, objectives, eligibility criteria, sources of evidence, charting methods, results, and conclusions that relate to the review questions and objectives. | Abstract |
| **INTRODUCTION** | | | |
| Rationale | 3 | Describe the rationale for the review in the context of what is already known. Explain why the review questions/objectives lend themselves to a scoping review approach. | 3-6 |
| Objectives | 4 | Provide an explicit statement of the questions and objectives being addressed with reference to their key elements (e.g., population or participants, concepts, and context) or other relevant key elements used to conceptualize the review questions and/or objectives. | 6 |
| **METHODS** | | | |
| Protocol and registration | 5 | Indicate whether a review protocol exists; state if and where it can be accessed (e.g., a Web address); and if available, provide registration information, including the registration number. | 7 |
| Eligibility criteria | 6 | Specify characteristics of the sources of evidence used as eligibility criteria (e.g., years considered, language, and publication status), and provide a rationale. | 8 |
| Information sources* | 7 | Describe all information sources in the search (e.g., databases with dates of coverage and contact with authors to identify additional sources), as well as the date the most recent search was executed. | 7 |
| Search | 8 | Present the full electronic search strategy for at least 1 database, including any limits used, such that it could be repeated. | S1 |
| Selection of sources of evidence† | 9 | State the process for selecting sources of evidence (i.e., screening and eligibility) included in the scoping review. | 8 |
| Data charting process‡ | 10 | Describe the methods of charting data from the included sources of evidence (e.g., calibrated forms or forms that have been tested by the team before their use, and whether data charting was done independently or in duplicate) and any processes for obtaining and confirming data from investigators. | 9 |
| Data items | 11 | List and define all variables for which data were sought and any assumptions and simplifications made. | 9 |
| Critical appraisal of individual sources of evidence§ | 12 | If done, provide a rationale for conducting a critical appraisal of included sources of evidence; describe the methods used and how this information was used in any data synthesis (if appropriate). | N/A |
| Synthesis of results | 13 | Describe the methods of handling and summarizing the data that were charted. | 9-10 |
| **RESULTS** | | | |
| Selection of sources of evidence | 14 | Give numbers of sources of evidence screened, assessed for eligibility, and included in the review, with reasons for exclusions at each stage, ideally using a flow diagram. | 12 |
| Characteristics of sources of evidence | 15 | For each source of evidence, present characteristics for which data were charted and provide the citations. | 10-22 and Suppl table S7 |
| Critical appraisal within sources of evidence | 16 | If done, present data on critical appraisal of included sources of evidence (see item 12). | N/A |
| Results of individual sources of evidence | 17 | For each included source of evidence, present the relevant data that were charted that relate to the review questions and objectives. | Suppl table S7 |
| Synthesis of results | 18 | Summarize and/or present the charting results as they relate to the review questions and objectives. | 10-22 |
| **DISCUSSION** | | | |
| Summary of evidence | 19 | Summarize the main results (including an overview of concepts, themes, and types of evidence available), link to the review questions and objectives, and consider the relevance to key groups. | 22-29- |
| Limitations | 20 | Discuss the limitations of the scoping review process. | 28-29 |
| Conclusions | 21 | Provide a general interpretation of the results with respect to the review questions and objectives, as well as potential implications and/or next steps. | 22-29 |
| **FUNDING** | | | |
| Funding | 22 | Describe sources of funding for the included sources of evidence, as well as sources of funding for the scoping review. Describe the role of the funders of the scoping review. | 29 |

JBI = Joanna Briggs Institute; PRISMA-ScR = Preferred Reporting Items for Systematic reviews and Meta-Analyses extension for Scoping Reviews.

* Where *sources of evidence* (see second footnote) are compiled from, such as bibliographic databases, social media platforms, and Web sites.

† A more inclusive/heterogeneous term used to account for the different types of evidence or data sources (e.g., quantitative and/or qualitative research, expert opinion, and policy documents) that may be eligible in a scoping review as opposed to only studies. This is not to be confused with *information sources* (see first footnote).

‡ The frameworks by Arksey and O’Malley (6) and Levac and colleagues (7) and the JBI guidance (4, 5) refer to the process of data extraction in a scoping review as data charting*.*

§ The process of systematically examining research evidence to assess its validity, results, and relevance before using it to inform a decision. This term is used for items 12 and 19 instead of "risk of bias" (which is more applicable to systematic reviews of interventions) to include and acknowledge the various sources of evidence that may be used in a scoping review (e.g., quantitative and/or qualitative research, expert opinion, and policy document).

*From:* Tricco AC, Lillie E, Zarin W, O'Brien KK, Colquhoun H, Levac D, et al. PRISMA Extension for Scoping Reviews (PRISMAScR): Checklist and Explanation. Ann Intern Med. 2018;169:467–473. [doi: 10.7326/M18-0850](http://annals.org/aim/fullarticle/2700389/prisma-extension-scoping-reviews-prisma-scr-checklist-explanation).

# **Table S7. Extraction data for each article in descending order by year**

| id | yr | Journal | Authors | Funding | Funding category | Keywords |
| --- | --- | --- | --- | --- | --- | --- |
| 613 | 2022 | American Journal of Emergency Medicine | Dora-Laskey, A., King, A., Sadler, R. | not reported | Not reported | architectural accessibility/statistics & numerical data, buprenorphine/therapeutic use, emergency service, hospital/statistics & numerical data, health services accessibility/statistics & numerical data, hospital bed capacity/statistics & numerical data, hospitalization/statistics & numerical data, humans, medicaid, michigan, narcotic antagonists/therapeutic use, opiate overdose/epidemiology, opioid-related disorders/drug therapy, socioeconomic factors, spatial analysis, united states, buprenorphine, emergency medicine, opioid use disorder, no financial relationships with commercial entities. |
| 614 | 2022 | Journal of General Internal Medicine | Hohman, J. A., Martinez, K. A., Anand, A., Rood, M., Martyn, T., Rose, S., Rothberg, M. B. | not reported | Not reported | none listed |
| 615 | 2022 | Psychiatric Services | Kalb, L. G., Holingue, C., Stapp, E. K., Van Eck, K., Thrul, J. | not reported | Not reported | crisis intervention, emergency care, epidemiology, serious mental illness |
| 616 | 2022 | Journal of General Internal Medicine | Kyanko, K. A., Curry, L. A., Keene, D. E., Sutherland, R., Naik, K., Busch, S. H. | National Institute of Mental Health | Government | mental health access, primary care, private health insurance |
| 618 | 2022 | Journal of Pediatric Surgery | Ridings, L. E., Espeleta, H. C., Streck, C. J., Davidson, T. M., Litvitskiy, N., Bravoco, O., Kassam-Adams, N., Ruggiero, K. J. | Medical University of South Carolina (MUSC) Technology Applications Center for Healthful Lifestyles pilot initiative, National Institute of Child Health and Human Development, National Institute of Mental Health, The Duke Endowment, SmartState South Carolina Centers of Economic Excellence | University, Government, Government, University, Government | behavioral health, mental health, pediatric injury, screening, trauma center, traumatic stress |
| 619 | 2022 | Journal of Clinical Psychology | Silverman, A. L., Teachman, B. A. | not reported | Not reported | implicit and explicit attitudes, mental health disparities, treatment access, treatment attitudes, treatment preferences |
| 620 | 2022 | Journal of Substance Abuse Treatment | Solomon, K. T., Bandara, S., Reynolds, I. S., Krawczyk, N., Saloner, B., Stuart, E. A., Connolly, E. | not reported | Not reported | medication availability, medication treatment, medication use, opioid use disorder, outpatient treatment facilities, residential treatment facilities |
| 622 | 2022 | Graduate Thesis | Taylor, K. J. J. | not reported | Not reported | access, african americans, case management, healthcare, blacks, case management, medicaid, mental health, outpatients, mental health services, health care access |
| 548 | 2021 | Alkoholizm I Narkomania-Alchoholism and Drug Addition | Abikoye, G., Okonkwo, E., Obot, I. | none | No funding | drug addiction, bunk patrons, treat-ment needs, facilities, uyo, substance use, disorders, abuse, risk, substance abuse |
| 465 | 2021 | Psychiatric Services | Abraham, A. J., Yarbrough, C. R., Harris, S. J., Bagwell-Adams, G., Andrews, C. M. | not reported | Not reported | none listed |
| 549 | 2021 | Journal of studies on alcohol and drugs | Abraham, A. J., Yarbrough, C. R., Harris, S. J., Bagwell-Adams, G., Andrews, C. M. | not reported | Not reported | alcoholism/epidemiology, alcoholism/therapy, opioid-related disorders, pharmaceutical preparations, alcohol drinking, humans, rural population, united states/epidemiology |
| 551 | 2021 | Mental Health and Social Inclusion | Alenezi, A. F., Aljowder, A., Almarzooqi, M. J., Alsayed, M., Aldoseri, R., Alhaj, O., Souraya, S., Thornicroft, G., Jahrami, H. | none | No funding | translation, validation, mental health problems, social stigma, psychometric study, mental-health-services, cultural-adaptation, systematic analysis, global, burden, stigma, discrimination, disorders, illness, prevalence, countries, social work |
| 552 | 2021 | Plos One | Alleaume, C., Verger, P., Peretti-Watel, P., Coconel Group. | Agence Nationale de la Recheche | Government | mental-health-services, help-seeking, gender-differences, anxiety, disorder, depression, impact, association, epidemic, quality, people, science & technology - other topics |
| 472 | 2021 | International Journal of Environmental Research and Public Health | Benjamen, J., Girard, V., Jamani, S., Magwood, O., Holland, T., Sharfuddin, N., Pottie, K. | none | No funding | adult, article, canada, care behavior, community mental health service, coronavirus disease 2019, health equity, health survey, human, mental disease, pandemic, primary medical care, privacy, psychotherapy, refugee, semi structured interview, social determinants of health |
| 473 | 2021 | Journal of Substance Abuse Treatment | Bensley, K. M. K., Karriker-Jaffe, K. J., Cherpitel, C., Li, L. B., Wallisch, L. S., Zemore, S. E. | National Institute on Alcohol Abuse and Alcoholism | Government | alcohol use disorder |
| 474 | 2021 | Academic Psychiatry | Bettencourt, A. F., Ferro, R. A., Williams, J. L. L., Khan, K. N., Platt, R. E., Sweeney, S., Coble, K. | Maryland Department of Health, Behavioral Health Administration, Health Resources and Services Administration | Government, Government | child psychiatry, integrated care, pediatrics, primary care providers, provider comfort |
| 525 | 2021 | Social Psychiatry and Psychiatric Epidemiology | Bhavsar, V., Jannesari, S., McGuire, P., MacCabe, J. H., Das-Munshi, J., Bhugra, D., Dorrington, S., Brown, J. S. L., Hotopf, M. H., Hatch, S. L. | Janssen, MSD, UCB Biogen, Lundbeck | Industry, Industry, Industry, Industry | common mental disorders, ethnicity, health inequalities, migration, psychological treatment |
| 559 | 2021 | Women and Birth | Bilardi, J. E., Sharp, G., Payne, S., Temple-Smith, M. J. | not reported | Not reported | miscarriage, spontaneous abortion, psychosocial support systems, health, personnel, qualitative research, emotions, psychological morbidity, pregnancy, experience, grief, depression, partners, sequelae, anxiety, impact, nursing, obstetrics & gynecology |
| 560 | 2021 | International Journal of Lean Six Sigma | Boukherroub, T., Ouellet, L., Lemay, G., Bibeau, N., Thiffault, D., McNeil, N. | not reported | Not reported | action research, kaizen, lean six sigma, accessibility, youths in, difficulty, frontline psychosocial services, lean health-care, management, paradigm, barriers, thinking, engineering, business & economics |
| 476 | 2021 | BMJ Open | Brice, S., Rodgers, J., Ingham, B., Mason, D., Wilson, C., Freeston, M., Le Couteur, A., Parr, J. R. | Autistica, Inge Wakehurst Trust | Foundation/Association, Foundation/Association | autistic disorder, adult, cross-sectional studies, delivery of health care, humans, surveys and questionnaires, united kingdom, mental health, organisational development, quality in health care |
| 478 | 2021 | Medical Care | Cantor, J. H., McBain, R., Kofner, A., Stein, B. D., Yu, H. | National Institute of Mental Health | Government | adult, article, child, controlled study, coronavirus disease 2019, cross-sectional study, female, human, major clinical study, male, medicaid, mental health, outcome assessment, outpatient, pandemic, public sector, shelter-in-place, telehealth, united states |
| 562 | 2021 | Drug & Alcohol Dependence | Cantor, J. H., Powell, D., Kofner, A., Stein, B. D. | not reported | Not reported | buprenorphine, opioid-related disorders/drug therapy, opioid-related disorders/epidemiology, health facilities, humans, access to care, medications for opioid use disorder |
| 479 | 2021 | Higher Education | Chang, W. P., Chen, T., Stuart, H., Chen, S. P. | not reported | Not reported | mental health, post-secondary institution, mental wellness toolkit, university-students, college-students, health-services, depression, self, education & educational research |
| 528 | 2021 | Australian Psychologist | Cronin, T. J., Pepping, C. A., Halford, W. K., Lyons, A., , , , , , , , , , , , , | not reported | Not reported | lgbtq+ persons -- psychosocial factors -- australia, mental health -- evaluation, help seeking behavior, health services accessibility, mental health services -- utilization, human, australia, adult, urban areas, rural areas, questionnaires, stress, psychological, descriptive statistics, psychological distress |
| 484 | 2021 | Psychiatric Services | Cummings, J. R., Smith, J. L., Cullen, S. W., Marcus, S. C. | not reported | Not reported | adult, article, community mental health, controlled study, crisis intervention, epidemic, human, mental health service, outpatient, risk factor, substance use, suicide |
| 485 | 2021 | Public Health Nursing | Dalstrom, M. D., Weinzimmer, L. G., Foulger, R., Klein, C. J. | none | No funding | affordable care act, health care access, illinois, medicaid, rural, population, public, environmental & occupational health, nursing |
| 569 | 2021 | Population Research and Policy Review | Elton, E., Gonzales, G. | not reported | Not reported | lgbt health, access to care, same-sex marriage, marital-status, women, disparities, attitudes, marriage, adults, gender, risk, gay, demography |
| 489 | 2021 | Journal of Ambulatory Care Management | Fortney, J. C., Pyne, J. M., Hawrilenko, M., Bechtel, J. M., Moore, D., Nolan, J. P., Pfeiffer, P., Shushan, S., Shore, J. H., Bowen, D. | Patient Centered Outcomes Research Institute | Government | psychiatric patients -- psychosocial factors, mental health services -- utilization, health services accessibility -- evaluation, patient attitudes -- evaluation, psychometrics -- evaluation, instrument validation, human, male, female, adult, middle age, validation studies, questionnaires, internal consistency, criterion-related validity, t-tests, pearson's correlation coefficient, regression, interviews, test-retest reliability, item analysis, telepsychiatry, social determinants of health, quality of life, depression -- symptoms, mania -- symptoms, stress disorders, post-traumatic, anxiety, attitude to health, socioeconomic factors, insurance coverage, employment status, health literacy, physician-patient relations, trust, privacy and confidentiality, internet access, attitude measures, checklists, scales, funding source |
| 574 | 2021 | Health Services Research | Kong, Y. F., Zhou, J., Zheng, Z. M., Amaro, H., Guerrero, E. G. | National Institute on Drug Abuse, National Institute on Minority Health and Disparities | Government, Government | racial disparities, regression tree, subgroup analysis, virtual twins, wait time for opioid treatment, substance-abuse treatment, use disorder, ethnic disparities, health, identification, completion, selection, trials, health care sciences & services |
| 502 | 2021 | Drug & Alcohol Dependence | Krawczyk, N., Garrett, B., Ahmad, N. J., Patel, E., Solomon, K. T., Stuart, E. A., Saloner, B. | Bloomberg Philanthropies | Foundation/Association | american indians, medications for opioid use disorder, native americans, opioids, overdose, treatment |
| 576 | 2021 | Health Equity | Lee, H., Singh, G. K. | Health Resources and Services Administration | Government | covid-19, access to care, delayed medical care due to pandemic, income inequalities, mental health services, trend |
| 577 | 2021 | JGIM: Journal of General Internal Medicine | Lewis, V. A., Spivack, S., Murray, G. F., Rodriguez, H. P. | National Institute on Aging, Agency for Healthcare Research and Quality | Government, Government | medically uninsured, safety-net providers, human, primary health care, adult, united states, medicaid, special populations, comparative studies, multicenter studies, evaluation research, validation studies, funding source |
| 506 | 2021 | BMC Palliative Care | McInnerney, D., Candy, B., Stone, P., Atkin, N., Johnson, J., Hiskey, S., Kupeli, N. | Marie Curie, Economic and Social Research Council, Alzheimer's Society, Univeristy College London Hospitals NIHR Biomedical Research Centre | Foundation/Association, Government, Foundation/Association, University | hospice patients -- psychosocial factors -- united kingdom, support, psychosocial -- evaluation -- in adulthood, attitude of health personnel -- evaluation, quality of health care, health services accessibility, psychiatric care, hospices, human, united kingdom, adult, cross sectional studies, descriptive statistics, referral and consultation, financial support, audit, questionnaires, national institute for health and care excellence, mental health |
| 507 | 2021 | Journal of Paediatrics and Child Health | McLean, K., Hiscock, H., Scott, D., Goldfeld, S. | State Government of Victoria’s Operational Infra-structure Support Program, Australian Government Research Training Program Scholarship, Murdoch Children’s Research Institutescholarship, Australian National Health and Medical Research Council Career Development Fellowship | Government, Government, Foundation/Association, Government | home care services, mental health services, adolescent, aged, caregivers, child, foster home care, health services accessibility, humans, medicare, united states, foster home care, health services accessibility, health services need and demand, survey and questionnaire |
| 582 | 2021 | Journal of Rural Health | Newman, M. W., Hawrilenko, M., Jakupcak, M., Chen, S., Fortney, J. C. | Patient Centered Outcomes Research Institute | Government | access to care, engagement, mental health, rural, stigma |
| 585 | 2021 | Journal of Gay & Lesbian Social Services | Qeadan, F., Akofua Mensah, N., Gu, L. Y., Barbeau, W. A., Madden, E. F., Porucznik, C. A., English, K. | not reported | Not reported | lgbtq, sud, tailored programs, treatment facilities |
| 586 | 2021 | International Journal of Transgender Health | Radusky, P. D., Cardozo, N., Duarte, M., Fabian, S., Frontini, E., Sued, O., Aristegui, I. | none | No funding | argentina, covid-19, health care, lockdown, mental health, non-binary, transgender, gender identity, stigma, psychology, public, environmental & occupational health, social sciences, other topics, biomedical social sciences |
| 588 | 2021 | Health Informatics Journal | Rush, K. L., Seaton, C., Li, E., Oelke, N. D., Pesut, B. | University of British Columbia Okanagan’s Eminence Program, Regional Socio-Economic Development Institute of Canada Fund | University, Government | covid-19, health literacy, telemedicine, canada, cross-sectional studies, female, health services, humans, male, pandemics, sars-cov-2, surveys and questionnaires, ehealth literacy, mental health, telehealth, telemedicine |
| 590 | 2021 | Psychiatric Services | Shover, C. L. | Wu Tsai Neurosciences Institute, National Institute on Drug Abuse | University, Government | buprenorphine, opioid use disorders, medicaid, buprenorphine, substance use disorder, opioid use disorder, drug therapy, methadone, substance use treatment |
| 592 | 2021 | Psychological Services | Smith, C. A., Boden, M. T., Trafton, J. A. | not reported | Not reported | staffing model, staffing ratio, supply-and-demand |
| 519 | 2021 | Archives of Gerontology and Geriatrics | Teles, S., Ferreira, A., Paúl, C. | Portuguese Foundation for Science and Technology | Government | caregivers, dementia/therapy, adaptation, psychological, cross-sectional studies, humans, respite care, access, dementia, dropout, informal caregivers, psychosocial interventions |
| 522 | 2021 | Journal of Rural Mental Health | Weinzimmer, L. G., Dalstrom, M. D., Klein, C. J., Foulger, R., de Ramirez, S. S. | none | No funding | telehealth, access, counseling, mental health, rural-urban locations, rural environments, telemedicine, health care access, interests, internet, urban environments |
| 608 | 2021 | Psychiatric Services | Williams, N. D., Turpin, R. E., Akre, E. R. L., Boekeloo, B. O., Fish, J. N. | not reported | Not reported | lgbtq, mental health access, mental health disparities, mental health treatment utilization |
| 609 | 2021 | Journal of Surgical Education | Wothe, J., Bosacker, L., Nalluri, H., Cullen, M. J., Brunsvold, M. E. | not reported | Not reported | mental health, mentors, surveys and questionnaires, work-life balance |
| 467 | 2020 | Journal of Adolescent Health | Alinsky, R. H., Hadland, S. E., Matson, P. A., Cerda, M., Saloner, B. | not reported | Not reported | substance abuse -- in adolescence, health facilities -- united states, drugs, substance use disorders, human, adult, adolescence, united states, cross sectional studies, descriptive statistics, logistic regression, confidence intervals, odds ratio, chi square test, data analysis software, funding source |
| 468 | 2020 | Journal of General Internal Medicine | Arega, M. A., Dee, E. C., Muralidhar, V., Nguyen, P. L., Franco, I., Mahal, B. A., Sanford, N. N. | Prostate Cancer Foundation, American Society for Radiation Oncology, Bayer, Astellas, Ferring, Dendreon, Blue Earth, Genome Dx, Augmenix, Boston Scientific, Janssen, Cota Healthcare, Sarepta Therapeutics | Foundation/Association, Foundation/Association, Industry, Industry, Industry, Industry, Industry, Industry, Industry, Industry, Industry, Industry, Industry | health care sciences & services, general & internal medicine |
| 470 | 2020 | Plos One | Argento, E., Goldenberg, S., Braschel, M., Machat, S., Strathdee, S. A., Shannon, K. | National Institute on Drug Abuse, Canadian Institutes of Health Research, MacAIDS, Canada Research Chair in Global Sexual Health and HIV/AIDS, Michael Smith Foundation | Government, Government, Foundation/Association, Government, Foundation/Association | health services accessibility/legislation & jurisprudence, sex work/legislation & jurisprudence, sex workers/legislation & jurisprudence, adult, canada, female, humans, male, prospective studies, transgender persons |
| 475 | 2020 | Health Services Research | Blunt, E. O., Maclean, J. C., Popovici, I., Marcus, S. C. | not reported | Not reported | availability, health care, mental illness, public insurance |
| 477 | 2020 | Brazilian Journal of Physical Therapy | Britto, R. R., Supervia, M., Turk-Adawi, K., Chaves, G. S. D., Pesah, E., Lopez-Jimenez, F., Pereira, D. A. G., Herdy, A. H., Grace, S. L. | York University Canada, Conselho Nacional de Pesquisa Brazil, Coordenacao de Aperfeicoamento de Pessoal de Nivel Superior, Brazil and Fundacao de Pesquisa de Minas Gerais Brazil | University, Government, Government, Government | rehabilitation, availability, health services, upper-middle income, country, association task-force, cardiovascular prevention, accf/aha guideline, management, components, trends, model, orthopedics, rehabilitation |
| 527 | 2020 | Journal of Homosexuality | Cronin, T. J., Pepping, C. A., Halford, W. K., Lyons, A., , , , , , , , , , , , , | not reported | Not reported | sexual minority, lesbian, gay, bisexual, mental health, minority stress, theory, barriers to service access, mental-health-services, orientation differences, stigma consciousness, bisexual men, gay men, depression, care, anxiety, lesbians, scales, psychology, social sciences - other topics |
| 486 | 2020 | MMWR: Morbidity & Mortality Weekly Report | DiGiulio, A., Jump, Z., Babb, S., Schecter, A., Williams, K. A. S., Yembra, D., Armour, B. S. | not reported | Not reported | medicaid -- economics, smoking cessation, insurance coverage -- statistics and numerical data, health services accessibility, united states, adult, smoking -- epidemiology, clinical assessment tools |
| 487 | 2020 | Journal of Substance Abuse Treatment | Drake, C., Donohue, J. M., Nagy, D., Mair, C., Kraemer, K. L., Wallace, D. J. | National Center for Advancing Translational Sciences, National Heart, Lung, and Blood Institute | Government, Government | buprenorphine, geographic access, medicaid, medications for opioid use disorder, opioid use disorder, public transit |
| 490 | 2020 | Transportation Research Part D-Transport and Environment | Ghorbanzadeh, M., Kim, K., Ozguven, E. E., Horner, M. W. | not reported | Not reported | spatial accessibility, mental health services, vulnerable populations, geographic information systems, ecological inference, care, environmental sciences & ecology, transportation |
| 492 | 2020 | Graduate Thesis | Gonzalez, R. L. | not reported | Not reported | mental health care, social stigma, help-seeking, risk factors, attitudes, health care seeking behavior, mental health, stigma, treatment, rural environments, well being |
| 493 | 2020 | Administration and Policy in Mental Health and Mental Health Services Research | Graaf, G., Snowden, L. R. | none | No funding | health services accessibility, insurance coverage, insurance, health, mental health services, public health, adolescent, child, child, preschool, female, health services needs and demand, health surveys, humans, infant, male, neurodevelopmental disorders/therapy, problem behavior, united states, children’s mental health, health insurance, medicaid, mental health access, serious emotional disturbance |
| 494 | 2020 | JAMA Network Open | Graves, J. M., Abshire, D. A., Mackelprang, J. L., Amiri, S., Beck, A. | Health Equity Research Center Washington University, National Institute on Minority Health and Disparities | University, Government | suicide -- prevention and control -- in adolescence, hospitals, psychiatric -- statistics and numerical data -- united states, mental health services, rural health services, human, adolescence, united states, cross sectional studies, data analysis software, nonparametric statistics, geographic factors, descriptive statistics, health services accessibility |
| 496 | 2020 | Health Affairs | Harris, S. J., Abraham, A. J., Andrews, C. M., Yarbrough, C. R. | not reported | Not reported | substance use disorders -- drug therapy, narcotics, medicare, health services accessibility, human, united states food and drug administration, buprenorphine, naltrexone, methadone, t-tests, logistic regression, insurance coverage, medicaid, united states, odds ratio |
| 497 | 2020 | JAMA Network Open | Huhn, A. S., Hobelmann, J. G., Strickland, J. C., Oyler, G. A., Bergeria, C. L., Umbricht, A., Dunn, K. E. | National Institute on Drug Abuse, Ashley Addiction Treatment | Government, Industry | analgesics, opioid/supply & distribution, health services accessibility/statistics & numerical data, opiate substitution treatment/statistics & numerical data, opioid-related disorders/drug therapy, residential treatment/statistics & numerical data, substance abuse treatment centers/statistics & numerical data, buprenorphine/supply & distribution, cross-sectional studies, humans, medicaid, methadone/supply & distribution, naltrexone/supply & distribution, united states |
| 498 | 2020 | BMC Psychiatry | Hung, P., Busch, S. H., Shih, Y. W., McGregor, A. J., Wang, S. | National Institute of Mental Health | Government | community mental health services/methods, health services accessibility/statistics & numerical data, suicide/statistics & numerical data, adolescent, adult, aged, aged, 80 and over, community mental health centers, community mental health services/trends, humans, middle aged, mortality/trends, retrospective studies, suicide/psychology, united states/epidemiology, young adult, access to mental health care, community mental health, deinstitutionalization, suicide |
| 533 | 2020 | Journal of Aging and Health | Johnson, Pa. J., Jou, J., Upchurch, D. M. | National Institute of Child Health and Human Development | Government | access to care, mental health, midlife, mental-health-services, unmet need, gender-differences, behavioral-model, screening scales, insurance status, united-states, medical-care, disparities, depression, geriatrics & gerontology, health care sciences & services |
| 500 | 2020 | BMC Psychiatry | Kiselev, N., Morina, N., Schick, M., Watzke, B., Schnyder, U., Pfaltz, M. C. | Swiss State Secretariat for Education, Research, and Innovation | Government | refugees, health services needs and demand, humans, mental health, outpatients, switzerland, asylum seekers, barriers, interpreters, mental health care services, refugee mental health, switzerland |
| 501 | 2020 | Aging & Mental Health | Knight, B. G., Winterbotham, S. | not reported | Not reported | rural areas, urban areas, mental health services, health services accessibility -- evaluation -- in old age, patient attitudes -- evaluation -- in old age, human, aged, australia, surveys, focus groups, interviews, help seeking behavior -- evaluation, health resource utilization -- evaluation, health knowledge -- evaluation, depression, substance abuse, stigma |
| 503 | 2020 | European Journal of Health Economics | Kreutzberg, A., Jacobs, R. | Centre for Health Economics University of York | University | waiting time targets, mental health, early intervention in psychosis, difference-in-difference analysis, early intervention services, outcome scales honos, health-care, performance, schizophrenia, policy, score, business & economics, health care sciences & services |
| 135 | 2020 | Epidemiology and Psychiatric Sciences | Lora, A., Hanna, F., Chisholm, D. | none | No funding | community mental health, inpatient psychiatry, outpatient psychiatry, psychiatric hospital, psychiatric services |
| 508 | 2020 | European Journal of Public Health | Michaud, P. A., Visser, A., Vervoort, J. P. M., Kocken, P., Reijneveld, S. A., Jansen, D. E. M. C. | European Commission through the Horizon 2020 Framework under the grant agreement number 634201 (Models of Child Health Appraised), Swiss State Secretariat for Education, Research, and Innovation | Government, Government | health services accessibility, mental health services, adolescent health services, primary health care, human, adolescence, european union, questionnaires, health screening, ambulatory care facilities, vulnerability, health care delivery |
| 537 | 2020 | Mental Health Review Journal | Oates, L. L., Firth, N. | Health Education East Midlands | Government | mental health services -- united kingdom, health services accessibility -- united kingdom, treatment outcomes -- evaluation, healthcare disparities, human, retrospective design, correlation coefficient, linear regression, fisher's exact test, record review, appointments and schedules, prevalence, comorbidity, functional status, scales, united kingdom |
| 60 | 2020 | International Journal of Geriatric Psychiatry | Ogliari, G., Turner, Z., Khalique, J., Gordon, A. L., Gladman, J. R. F., Chadborn, N. H. | NIHR Collaboration for Leadership in Applied Health Research, Care East Midlands | Government, Government | south asian ethnicity, aged, barriers to mental healthcare, cohort study, dementia, gender differences, healthcare disparities, memory assessment services, outpatients |
| 509 | 2020 | Administration and Policy in Mental Health and Mental Health Services Research | Ojeda, V. D., Munson, M. R., Jones, N., Berliant, E., Gilmer, T. P. | National Institute on Minority Health and Disparities | Government | minority youth, mental health service use, disparities |
| 510 | 2020 | Journal of the American Geriatrics Society | Orth, J., Li, Y., Simning, A., Temkin-Greener, H. | not reported | Not reported | aged, aggression, article, cross-sectional study, depression, female, health care availability, health service, human, major clinical study, male, nursing home, nursing home patient, sample size, self concept, staff training, suicidal ideation |
| 512 | 2020 | Plos One | Parcesepe, A. M., Lancaster, K., Edelman, E. J., DeBoni, R., Ross, J., Atwoli, L., Tlali, M., Althoff, K., Tine, J., Duda, S. N., Wester, C. W., Nash, D. | National Institutes of Allergy and Infectious Diseases, National Institute of Child Health and Human Development, National Cancer Institute, National Institute on Drug Abuse, National Heart, Lung, and Blood Institute, National Institute on Alcohol Abuse and Alcoholism, National Institute of Diabetes and Digestive Kidney Diseases, Fogarty International Center, National Library of Medicine, National Center for Advancing Translational Sciences, Harmonist Project | Government, Government, Government, Government, Government, Government, Government, Government, Government, Government, Government | delivery of health care, integrated/organization & administration, hiv infections/therapy, health services accessibility/organization & administration, substance abuse treatment centers/organization & administration, substance-related disorders/rehabilitation, adolescent, adult, age factors, child, delivery of health care, integrated/statistics & numerical data, global health, hiv infections/complications, health services accessibility/statistics & numerical data, humans, mass screening/organization & administration, mass screening/statistics & numerical data, patient education as topic, professional practice gaps/statistics & numerical data, program evaluation, referral and consultation/organization & administration, referral and consultation/statistics & numerical data, substance abuse treatment centers/statistics & numerical data, substance-related disorders/complications, substance-related disorders/diagnosis, surveys and questionnaires/statistics & numerical data, young adult |
| 514 | 2020 | Psychological Services | Pyne, J. M., Kelly, P. A., Fischer, E. P., Miller, C. J., Connolly, S. L., Wright, P., Zamora, K., Koenig, C. J., Seal, K.H., Fortney, J. C. | Veterans Administration | Government | access, mental health, veterans, validation |
| 156 | 2020 | Journal of Cancer Survivorship | Reinhart, C. A., Sae-Hau, M., Lee, C. A., Weiss, E. S. | The Leukemia & Lymphoma Society | Foundation/Association | health care delivery -- methods, hematologic neoplasms -- mortality, female, united states, national cancer institute (u.s.), human, validation studies, comparative studies, evaluation research, multicenter studies |
| 77 | 2020 | BMJ Open | Rocks, S., Fazel, M., Tsiachristas, A. | NIHR Collaboration for Leadership in Applied Health Research, Care Oxford at Oxford Health NHS Foundation Trust, Clinical Commissioning Groups in Oxfordshire and Buckinghamshire | Government, Government, Industry | child and adolescent psychiatry, health economics, mental health, organisation of health services |
| 539 | 2020 | BMC Health Services Research | Rocks, S., Glogowska, M., Stepney, M., Tsiachristas, A., Fazel, M. | National Institute for Health Research, Clinical Commissioning Groups in Oxfordshire and Buckinghamshire | Government, Industry | mental health services -- administration, adolescent health services -- administration, mental disorders -- therapy, health services accessibility, child health services -- administration, primary health care, child, male, health services research, qualitative studies, adolescence, female, referral and consultation -- statistics and numerical data, england |
| 517 | 2020 | BMJ Open Quality | Stafford, J., Aurelio, M., Shah, A. | not reported | Not reported | pdsa, continuous quality improvement, outpatients, quality improvement, waiting lists |
| 542 | 2020 | JMIR Mental Health | Too, L. S., Leach, L., Butterworth, P. | National Health and Medical Research Council Early Career Fellowship, Australian Research Council Future Fellowship, University of Melbourne Faculty of Medicine, Dentistry and Health Sciences Research Fellowship | Government, Government, University | affordability, mobile phone, internet access, mental health |
| 520 | 2020 | Journal of Pain Research | Voon, P., Wang, L., Nosova, E., Hayashi, K., Milloy, M. J., Wood, E., Kerr, T. | National Institute on Drug Abuse, Canadian Institutes of Health Research, The Pierre Elliott Trudeau Foundation, Michael Smith Foundation, St Paul’s Foundation | Government, Government, Foundation/Association, Foundation/Association, Foundation/Association | addiction, health services, methadone, opioid agonist treatment, pain, substance use |
| 521 | 2020 | Journal of Clinical Sport Psychology | Way, W. C., Coker-Cranney, A. M., Watson, J. C. | not reported | Not reported | counseling, outreach, screening, attitudes, barriers, psychology |
| 523 | 2020 | Health Services Research | Williams, N. D., Fish, J. N. | not reported | Not reported | mental health services/statistics & numerical data, sexual and gender minorities/statistics & numerical data, substance-related disorders/therapy, cultural competency, faith-based organizations/statistics & numerical data, female, health services accessibility/statistics & numerical data, health services research, humans, insurance, health, reimbursement/statistics & numerical data, logistic models, male, ownership/statistics & numerical data, patient acceptance of health care/statistics & numerical data, united states, mental health, sexual and gender minorities, substance abuse, treatment |
| 296 | 2019 | American Journal of Public Health | Andrews, C. M., Abraham, A. J., Grogan, C. M., Westlake, M. A., Pollack, H. A., Friedmann, P. D. | National Institute on Drug Abuse | Government | substance use rehabilitation programs, buprenorphine, medicaid, drugs, prescription, insurance coverage, human, surveys, odds ratio, confidence intervals |
| 404 | 2019 | Psychiatric Services | Brown, J. D. | not reported | Not reported | none listed |
| 368 | 2019 | Pediatrics | Coker, T. R., Porras-Javier, L., Zhang, L., Soares, N., Park, C., Patel, A., Tang, L., Chung, P., Zima, B. T. | Patient Centered Outcomes Research Institute, California Community Foundation | Government, Foundation/Association | california, care coordinator, checklist, child, community mental health, conference abstract, controlled study, depression, female, follow up, health center, household, human, major clinical study, male, medical record review, mental capacity, mental health care, outcome assessment, patient referral, primary medical care, quality of life, randomized controlled trial, satisfaction, telehealth, unemployment, videoconferencing |
| 384 | 2019 | American Journal of Public Health | Dedania, R., Gonzales, G. | not reported | Not reported | mental health services, healthcare disparities, mental health -- in adulthood -- united states, health status -- in adulthood -- united states, immigrants, human, comparative studies, united states, adolescence, young adult, adult, middle age, interview guides, prevalence, multiple logistic regression, health services accessibility, severity of disability, stress, psychological, emergency service, office visits, medical care |
| 251 | 2019 | Digestive Diseases & Sciences | Dunn, S. H., Rogal, S. S., Maier, M. M., Chartier, M., Morgan, T. R., Beste, L. A. | Veterans Administration | Government | quality assurance, health care, liver cirrhosis, gastroenterology, united states department of veterans affairs, veterans health, surveys and questionnaires |
| 197 | 2019 | Psychological Services | Gulliver, S. B., Pennington, M. L., Torres, V. A., Steffen, L. E., Mardikar, A., Leto, F., Ostiguy, W., Zimering, R. T., Kimbrel, N. A. | FEMA, Veterans Administration | Government, Government | social stigma, culturally competent care/statistics & numerical data, firefighters/statistics & numerical data, health services accessibility/statistics & numerical data, mental disorders/therapy, mental health services/statistics & numerical data, patient acceptance of health care/statistics & numerical data, adult, female, humans, male, middle aged, patient preference |
| 7 | 2019 | Drug & Alcohol Dependence | Huhn, A. S., Hobelmann, J. G., Ramirez, A., Strain, E. C., Oyler, G. A. | National Institute on Drug Abuse | Government | acamprosate, alcohol use disorder, mental health, naltrexone, older adults, treatment |
| 378 | 2019 | Behaviour Research and Therapy | Johns, L., Jolley, S., Garety, P., Khondoker, M., Fornells-Ambrojo, M., Onwumere, J., Peters, E., Milosh, C., Brabban, A., Byrne, M. | National Institute for Health Research | Government | none listed |
| 212 | 2019 | Psychological Services | Parnes, M. F., Bagrodia, R., Wightman, K., Singh-Sawhney, R., Satterthwaite, M. L., Knuckey, S., Bryant, R. A., Brown, A. D. | not reported | Not reported | human rights advocates, posstraumatic stress disorder, treatment access, cognitive appraisals, social support |
| 323 | 2019 | Military Medicine | Pyne, J. M., Kelly, P. A., Fischer, E. P., Miller, C. J., Wright, P., Zamora, K., Koenig, C. J., Stanley, R., Seal, K.H., Fortney, J. C. | Veterans Administration | Government | access, community healthcare, mental health, veterans |
| 346 | 2019 | BMC Health Services Research | Seo, V., Baggett, T. P., Thorndike, A. N., Hull, P., Hsu, J., Newhouse, J. P., Fung, V. | Agency for Healthcare Research and Quality | Government | community health centers/statistics & numerical data, health services accessibility/statistics & numerical data, medicaid/statistics & numerical data, medically uninsured/statistics & numerical data, patient protection and affordable care act/statistics & numerical data, adult, ethnic groups, female, health services research, health status disparities, humans, insurance coverage/statistics & numerical data, male, middle aged, prescription drugs, united states, young adult, access to care, community health centers, medicaid, safety-net, uninsured |
| 236 | 2019 | Health Affairs | Seshadri, R., Strane, D., Matone, M., Ruedisueli, K., Rubin, D. M. | not reported | Not reported | military personnel -- psychosocial factors, government programs -- evaluation, insurance coverage -- evaluation, pediatric care, quality of health care, health services accessibility, human, male, female, infant, newborn, infant, child, preschool, child, adolescence, cross sectional studies, health services research, surveys, logistic regression, odds ratio, confidence intervals, post hoc analysis, data analysis software, probability, behavioral and mental disorders, insurance carriers, healthcare disparities, health services needs and demand |
| 47 | 2019 | Drug & Alcohol Dependence | Shover, C. L., Humphreys, K. | National Institute on Drug Abuse, Veterans Administration, Wu Tsai Neurosciences Institute | Government, Government, University | buprenorphine, health services, moud, naltrexone, opioid use disorder, treatment |
| 420 | 2019 | Psychiatric Services | Spivak, S., Cullen, B. A., Green, C., Firth, T., Sater, H., Mojtabai, R. | not reported | Not reported | none listed |
| 314 | 2019 | The Australian and New Zealand Journal of Psychiatry | van Spijker, B. A., Salinas-Perez, J. A., Mendoza, J., Bell, T., Bagheri, N., Furst, M. A., Reynolds, J., Rock, D., Harvey, A., Rosen, A., Salvador-Carulla, L. | ConNetica Consultying, Brain and Mind Centre University of Sydney, Western NSW Primary Health Network, Western Australia Primary Health Alliance, Western Australia Mental Health Commission | Industry, University, Government, Government, Government | desde-ltc, rural mental health, remote mental health, service mapping, service provision |
| 154 | 2018 | Journal of Correctional Health Care | Baćak, V., Ridgeway, G. | none | No funding | correctional health services -- evaluation, health services accessibility, human, male, female, self report, mental health services, substance use rehabilitation programs, questionnaires, hiv infections -- drug therapy, regression |
| 341 | 2018 | Journal of the Canadian Academy of Child and Adolescent Psychiatry | Clark, S., Emberly, D., Pajer, K., Delong, E., McWilliam, S., Bagnell, A., Abidi, S., Casey, B., Gardner, W. | none | No funding | child and adolescent mental health, access, services, wait times, patient engagement |
| 224 | 2018 | Journal of Drug Issues | Haley, S. J., Moscou, S., Murray, S., Rieckmann, T., Wells, K. | United Hospital Fund, City of University of New York | Foundation/Association, University | community health centers, substance abuse, adolescents, alcohol, tobacco or other drugs, new york state |
| 370 | 2018 | Psychiatric Services | Hodgkin, D., Horgan, C. M., Stewart, M. T., Quinn, A. E., Creedon, T. B., Reif, S., Garnick, D. W. | Association for Behavioral Health and Wellness | Foundation/Association | federal parity, behavioral health care access, private health plans, health care psychology, health insurance, mental health parity, treatment planning, health care access, private sector |
| 248 | 2018 | Journal of Substance Abuse Treatment | Jones, C. W., Christman, Z., Smith, C. M., Safferman, M. R., Salzman, M., Baston, K., Haroz, R. | none | No funding | substance use disorders -- rehabilitation, overdose -- prevention and control, buprenorphine -- administration and dosage, cross sectional studies, overdose -- epidemiology, buprenorphine -- supply and distribution, physicians -- statistics and numerical data, substance use disorders -- complications, narcotic antagonists -- supply and distribution, overdose -- mortality, united states, narcotic antagonists -- administration and dosage, health services accessibility, scales |
| 5 | 2018 | Journal of Affective Disorders | König, D., Fellinger, M., Pruckner, N., Hinterbuchinger, B., Dorffner, G., Gleiss, A., Vyssoki, S., Vyssoki, B. | none | No funding | health services accessibility -- statistics and numerical data, suicide, mental health services -- statistics and numerical data, mental disorders -- therapy, mortality -- trends, adolescence, europe, adult, female, male |
| 268 | 2018 | Graduate Thesis | Kugelmass, H. | Fahs-Beck Fund, Princeton Center for the Study of Social Organization, Princeton Center for Health and Wellbeing, Princeton Department of Sociology | Foundation/Association, University, University, University | racial disparities, mental health care, health care access, health care utilization, mental health services, racial and ethnic differences |
| 217 | 2018 | Children and Youth Services Review | Maguire-Jack, K., Cao, Y., Yoon, S. | not reported | Not reported | racism, child abuse, social work service, blacks, whites, human, poverty, mental health, substance abuse |
| 462 | 2018 | Women's Health Issues | Moore, J. E., Mompe, A., Moy, E. | none | No funding | sex factors, quality of health care, health status, healthcare disparities -- trends, human, female, male, adolescence, adult, quality improvement, health services accessibility, patient safety, kidney failure, chronic, acquired immunodeficiency syndrome, mental health services, descriptive statistics |
| 265 | 2018 | Revista Panamericana de Salud Publica | Nickels, S. V., Campos Tomasino, M., Flamenco Arvaiza, N. A., Hunter, C. A. | not reported | Not reported | health services accessibility -- el salvador, mental health services -- el salvador, decentralization -- el salvador, psychiatric service -- el salvador, human, el salvador, outpatient service, family, caregivers, surveys, hospitals, psychiatric, health care costs, descriptive statistics, regression, government, referral and consultation, world health organization, community mental health services |
| 321 | 2018 | Administration and Policy in Mental Health and Mental Health Services Research | Novak, P., Anderson, A. C., Chen, J. | National Institute of Mental Health | Government | patient protection and affordable care act, health services accessibility/legislation & jurisprudence, insurance coverage/legislation & jurisprudence, insurance, health/legislation & jurisprudence, stress, psychological/therapy, adolescent, adult, female, health services accessibility/statistics & numerical data, humans, insurance coverage/statistics & numerical data, insurance, health/statistics & numerical data, male, middle aged, stress, psychological/epidemiology, surveys and questionnaires, united states/epidemiology, young adult, access to care, affordable care act, mental health, serious psychological distress |
| 413 | 2018 | BMC Health Services Research | Ohl, M. E., Carrell, M., Thurman, A., Weg, M. V., Pharm, T. H., Mengeling, M., Vaughan-Sarrazin, M. | Veterans Administration | Government | veterans, health manpower -- statistics and numerical data, rural health services -- manpower, health services accessibility, human, united states department of veterans affairs, health status, united states, income, decision making, validation studies, comparative studies, evaluation research, multicenter studies, questionnaires |
| 220 | 2018 | Medical Care | Ortega, A. N., McKenna, R. M., Kemmick Pintor, J., Langellier, B. A., Roby, D. H., Pourat, N., Vargas Bustamante, A., Wallace, S. P. | not reported | Not reported | mental health, hispanics -- statistics and numerical data, health status, health services accessibility -- statistics and numerical data, obesity -- ethnology, middle age, adult, hypertension -- ethnology, stress, psychological -- ethnology, immigrants -- statistics and numerical data, surveys, california, female, asthma -- ethnology, male, adolescence, young adult, socioeconomic factors, patient attitudes -- ethnology, clinical assessment tools |
| 441 | 2018 | BMJ Open Quality | Shah, A., Chitewe, A., Binley, E., Alom, F., Innes, J. | none | No funding | continuous quality improvement, control charts/run charts, mental health, pdsa |
| 187 | 2018 | International Journal of Mental Health Systems | Tirintica, A. R., Andjelkovic, I., Sota, O., Pirlog, M. C., Stoyanova, M., Mihai, A., Wallace, N. T. | University of California Berkeley | University | access, europe, factors, mental health services |
| 103 | 2017 | Addictive Behaviors | Abraham, A. J., Bagwell-Adams, G., Jayawardhana, J. | not reported | Not reported | state government, health services accessibility/statistics & numerical data, public policy/legislation & jurisprudence, substance abuse treatment centers/statistics & numerical data, tobacco industry/legislation & jurisprudence, tobacco use cessation/statistics & numerical data, health services accessibility/legislation & jurisprudence, humans, taxes/legislation & jurisprudence, taxes/statistics & numerical data, united states, counseling, pharmacotherapy, screening, state tobacco control policy, substance use disorder treatment programs, tobacco cessation services |
| 379 | 2017 | Psychiatric Services | Abraham, A. J., Rieckmann, T., Andrews, C. M., Jayawardhana, J. | not reported | Not reported | central nervous system agents -- therapeutic use, insurance, health -- statistics and numerical data, health services accessibility -- statistics and numerical data, medicaid -- statistics and numerical data, substance use disorders -- drug therapy, health benefit plans, employee -- statistics and numerical data, substance use disorders -- epidemiology, united states, human |
| 432 | 2017 | Graduate Thesis | Chen, C. | not reported | Not reported | cultural competence, school psychologists, school psychology services, cultural sensitivity, school psychology |
| 439 | 2017 | JAMA Psychiatry | Cummings, J. R., Allen, L., Clennon, J., Ji, X., Druss, B. G. | National Institute of Mental Health | Government | health services accessibility/statistics & numerical data, income/statistics & numerical data, mental health services/statistics & numerical data, poverty/statistics & numerical data, community mental health services/statistics & numerical data, humans, united states |
| 406 | 2017 | Health & Social Care in the Community | Gallego, G., Dew, A., Lincoln, M., Bundy, A., Chedid, R. J., Bulkeley, K., Brentnall, J., Veitch, C. | National Health and Medical Research Council, NSW Family and Community Services, Ageing, Disability, and Home Care Western Region, Faculty of Health Sciences University of Sydney | Government, Government, University | health services accessibility -- new south wales, disabled -- new south wales, rural health services -- new south wales, human, rural areas, cross sectional studies, exploratory research, new south wales, surveys, physical therapy, occupational therapy, speech-language pathology, mental health services, infant, child, preschool, child, adolescence, adult, middle age, aged, female, male, waiting lists, travel, caregivers, prospective studies, questionnaires, data analysis software, fisher's exact test, thematic analysis, coding, health care costs, funding source, chi square test |
| 226 | 2017 | Psychiatric Services | Gallo, K. P., Olin, S. S., Storfer-Isser, A., O'Connor, B. C., Whitmyre, E. D., Hoagwood, K. E., Horwitz, S. M. | National Institute of Mental Health, Children's Health Insurance Program Reauthorization Act | Government, Government | adolescents/adolescence, community mental health services, depression, mental health systems/hospitals, psychiatry/general |
| 325 | 2017 | Journal of Rural Health | Hirchak, K. A., Murphy, S. M. | not reported | Not reported | substance use disorders -- drug therapy, native americans, rural areas, urban areas, health services accessibility, human, washington, variable, regression, p-value |
| 424 | 2017 | Hospital Topics | Holstein, R. M., Paul Iii, D. P. | not reported | Not reported | mental health services -- new jersey, health services accessibility -- new jersey, waiting lists, surveys, telephone, human, female, male, descriptive statistics, data analysis software, p-value, chi square test, kruskal-wallis test, new jersey, medicaid, insurance carriers, psychiatrists, psychologists |
| 106 | 2017 | BMC Psychiatry | Kvig, E. I., Brinchmann, B., Moe, C., Nilssen, S., Larsen, T. K., Sorgaard, K. | Northern Norway Regional Health Authority | Government | dup, treatment delay, pathways, accessibility, psychosis |
| 403 | 2017 | Journal of Social Work Practice in the Addictions | Manuel, J. I. | National Institute on Drug Abuse | Government | healthcare disparities, sex factors, race factors, health services accessibility, health services needs and demand, alcohol abuse, patient protection and affordable care act, mental health services, human, blacks, male, female, asians, hispanics, quality improvement, health policy, surveys, stratified random sample, substance use disorders, variable, self report, dsm, adolescence, young adult, adult, middle age, educational status, health status, data analysis software, chi square test, pretest-posttest design, coding, p-value |
| 331 | 2017 | Journal of Social Work in Disability & Rehabilitation | McDonnall, M. C., Crudden, A., LeJeune, B. J., Steverson, A. C. | The Hellen Keller National Center for Deaf-Blind Youths and Adults, The Helmsley Charitable Trust | Mix, Foundation/Association | deaf-blind disorders -- rehabilitation, deafness -- psychosocial factors, community mental health services -- utilization, health services accessibility, human, health services for persons with disabilities, cultural competence, communication methods, total, surveys, open-ended questionnaires, mental health personnel, interviews, attitude of health personnel -- evaluation, audiorecording, thematic analysis, descriptive statistics, professional knowledge, organizational policies, health care delivery -- administration, health services needs and demand, staff development, funding source |
| 201 | 2017 | Substance Abuse: Research and Treatment | Parran, T. V., Muller, J. Z., Chernyak, E., Adelman, C., Delos Reyes, C. M., Rowland, D., Kolganov, M. | not reported | Not reported | buprenorphine, addiction, article, cross-sectional study, health care access, health care delivery, health care facility, health economics, health insurance, human, office based therapy, ohio, opiate addiction, physician attitude, prescription, urban area |
| 4 | 2017 | Australian Psychologist | Stargatt, J., Bhar, S. S., Davison, T. E., Pachana, N. A., Mitchell, L., Koder, D., Hunter, C., Doyle, C., Wells, Y., Helmes, E. | not reported | Not reported | aged care, geropsychology, mental health, older adults, residential care, psychological treatment, aged (attitudes toward), anxiety, residential care institutions, psychological distance |
| 427 | 2017 | Australasian Psychiatry | Thomas, N., Alfred, M., Foley, F., Lindblom, K., Lee, S. | State Government of Victoria Mental Illness Research Fund | Government | adult, article, australia, health care delivery, health survey, human, male, medical information, mental disease, middle aged, psychometry, psychosis, questionnaire, retrospective study, schizophrenia, social network, young adult |
| 122 | 2017 | Oncologist | Zimmermann-Schlegel, V., Hartmann, M., Sklenarova, H., Herzog, W., Haun, M. W. | German Federal Ministry of Health | Government | neoplasms, clinical oncology, health psychology, general practice, health services research |
| 165 | 2016 | Addictive Disorders & Their Treatment | Antoine, D., Heffernan, S., Chaudhry, A., King, V., Strain, E. C. | National Institute on Drug Abuse | Government | substance use disorders -- therapy, telerehabilitation, patient attitudes, health services accessibility, human, male, female, internet connections, cellular phone, surveys |
| 415 | 2016 | Journal of Addiction Medicine | Barry, D. T., Fazzino, T., Necrason, E., Ginn, J., Fiellin, L. E., Fiellin, D. A., Moore, B. A. | National Institute on Drug Abuse | Government | analgesics, opioid, substance abuse -- therapy, buprenorphine -- therapeutic use, multidisciplinary care team -- utilization, counseling -- utilization, human, descriptive statistics, research, surveys, physicians, physician's role, prescribing patterns, physician attitudes |
| 155 | 2016 | Health Affairs | Bishop, T. F., Seirup, J. K., Pincus, H. A., Ross, J. S. | National Institute on Aging, Weill Cornell Medical College, Commonwealth Fund, Yale University, Medtronic Inc., Johnson & Johnson, BlueCross BlueShield Association, Centers for Medicare and Medicaid Services, Food and Drug Administration | Government, University, Foundation/Association, University, Industry, Industry, Foundation/Association, Government, Government | psychiatrists, access to care, primary care, mental health, hospital referral, regions, mental disorders, populations, physcians, primary care providers, health care providers |
| 137 | 2016 | Health Affairs | Creedon, T. B., Lê Cook, B. | Agency for Healthcare Research and Quality | Government | health services accessibility, mental health services, substance abuse -- therapy -- united states, healthcare disparities, insurance coverage, mental disorders -- therapy -- united states, patient protection and affordable care act, medicaid, race factors, ethnic groups, descriptive statistics, p-value, united states, regression, adolescence, adult, middle age, scales, human, whites, blacks, hispanics, funding source |
| 366 | 2016 | Journal of Child Health Care | Henning-Smith, C., Alang, S. | National Institute of Child Health and Human Development | Government | affective symptoms, child behavior disorders, health services accessibility, child, chronic disease, health care surveys, humans, insurance coverage, mental health services, united states, access to care, children, emotional/behavioral difficulties, insurance |
| 444 | 2016 | Journal of Health and Social Behavior | Kugelmass, H. | Princeton Center for Health and Wellbeing | University | bias, field experiment, mental health care, race, social class |
| 352 | 2016 | American Journal of Physical Medicine & Rehabilitation | Moore, M., Jimenez, N., Rowhani-Rahbar, A., Willis, M., Baron, K., Giordano, J., Crawley, D., Rivara, F. P., Jaffe, K. M., Ebel, B. E. | National Center for Advancing Translational Sciences, National Institute of Child Health and Human Development | Government, Government | brain injuries -- rehabilitation -- in infancy and childhood, outpatient service, healthcare disparities, human, ecological research, cross sectional studies, washington, outpatients, communication skills, insurance, health, mental health services, health services accessibility, multilingualism, medicaid, male, female, infant, newborn, infant, child, preschool, child, adolescence |
| 50 | 2016 | Journal of the American Academy of Child & Adolescent Psychiatry | Olin, S. S., O'Connor, B. C., Storfer-Isser, A., Clark, L. J., Perkins, M., Hudson Scholle, S., Whitmyre, E. D., Hoagwood, K. E., Horwitz, S. M. | National Institute of Mental Health, Agency for Healthcare Research and Quality, Centers for Medicare and Medicaid Services | Government, Government, Government | adolescent health services -- standards, health services accessibility -- standards, ambulatory care -- standards, mental health services -- standards, mental health services -- statistics and numerical data, adolescence, ambulatory care -- statistics and numerical data, patient simulation, adolescent health services -- statistics and numerical data, health services accessibility -- statistics and numerical data, funding source, human |
| 464 | 2016 | Psychiatric Services | West, J. C., Clarke, D. E., Duffy, F. F., Barber, K. D., Mojtabai, R., Mościcki, E. K., Kroeger Ptakowski, K., Levin, S. | American Psychiatric Association Foundation | Foundation/Association | health care reform/statistics & numerical data, health services accessibility/statistics & numerical data, mental health services/statistics & numerical data, physicians/statistics & numerical data, psychiatry/statistics & numerical data, cross-sectional studies, humans |
| 425 | 2015 | Academic Pediatrics | Clemans-Cope, L., Kenney, G., Waidmann, T., Huntress, M., Anderson, N. | Department of Health and Human Services | Government | children's health insurance program, cost sharing, health expenditures, health services accessibility, medically uninsured, child health services/economics, adolescent, child, child health services/statistics & numerical data, child, preschool, female, humans, infant, insurance coverage, male, mental health services/economics, mental health services/statistics & numerical data, united states, chip, access and use of health care, affordability, comparison of health insurance coverage types, emergency department visit, health insurance adequacy, mental health visit, prescribed medicine, public health insurance, specialist care, unmet health care needs |
| 15 | 2015 | Australasian Psychiatry | Johnston, N. E. | not reported | Not reported | electroconvulsive therapy, rural mental health, service delivery, health disparities, electroconvulsive shock therapy, psychiatric hospitals, rural environments, mental health services |
| 158 | 2015 | Psychiatric Services | Knudsen, H. K., Roman, P. M., Ducharme, L. J. | National Institute on Alcohol Abuse and Alcoholism | Government | smoking cessation, insurance carriers -- statistics and numerical data, insurance coverage, medicaid, tobacco use cessation products, multivariate analysis, logistic regression, united states, substance use disorders -- therapy, counseling -- economics, bupropion -- therapeutic use, impact of events scale, funding source, human |
| 114 | 2015 | Journal of Child & Adolescent Substance Abuse | Mericle, A. A., Arria, A. M., Meyers, K., Cacciola, J., Winters, K. C., Kirby, K. | National Institute on Drug Abuse | Government | substance use disorders -- trends -- in adolescence, substance use disorders -- trends -- united states, substance use disorders -- therapy -- in adolescence, human, united states, survey research, child, adolescence, interviews, random sample, questionnaires, private sector, data analysis software, chi square test, mental health services, epidemiological research, statistical significance, substance use rehabilitation programs, confidence intervals, alcohol abuse, street drugs, funding source |
| 396 | 2015 | Environment and Planning B-Planning & Design | Morrissey, K., Clarke, G., Williamson, P., Daly, A., O'Donoghue, C. | not reported | Not reported | spatial microsimulation, propensity score matching, access to psychiatric hospitals, depression |
| 254 | 2015 | Journal of Adolescent Health | Muilenburg, J. L., Laschober, T. C., Eby, L. T. | National Institute on Drug Abuse | Government | substance use disorders -- psychosocial factors, psychotherapist attitudes, reports, smoking cessation programs, program implementation, health knowledge, human, adolescence, health and welfare planning, counselors, scales |
| 324 | 2015 | LGBT Health | Nemoto, T., Cruz, T. M., Iwamoto, M., Sakata, M. | not reported | Not reported | cities, african americans/statistics & numerical data, health services accessibility/statistics & numerical data, transgender persons/statistics & numerical data, adolescent, adult, aged, female, health status, humans, male, mental health services/statistics & numerical data, middle aged, needs assessment, sex workers/statistics & numerical data, social support, socioeconomic factors, substance-related disorders/epidemiology, united states, young adult, access to care, mental health needs, transgender |
| 68 | 2015 | Journal of Health Care for the Poor & Underserved | VanderWielen, L. M., Gilchrist, E. C., Nowels, M. A., Petterson, S. M., Rust, G., Miller, B. F. | not reported | Not reported | health services accessibility, mental health services, primary health care, ethnic groups, human, race factors, logistic regression, outpatients, rural areas, blacks, hispanics, geographic factors, health care delivery, integrated, surveys, minority groups, odds ratio, confidence intervals, urban areas, healthcare disparities, united states |
| 218 | 2015 | Health Services Research | Wen, H., Druss, B. G., Cummings, J. R. | not reported | Not reported | medicaid, access of services, demand of services, utilization of services, mental health, substance abuse |
| 435 | 2014 | Mayo Clinic Proceedings | Bobo, W. V., Wollan, P., Lewis, G., Bertram, S., Kurland, M. J., Vore, K., Yawn, B. P. | Agency for Healthcare Research and Quality, National Institute of Mental Health | Government, Government | depression, postpartum -- epidemiology, health services accessibility -- statistics and numerical data, insurance, health -- statistics and numerical data, adult, depression, postpartum -- diagnosis, depression, postpartum -- psychosocial factors, depression, postpartum -- therapy, female, health services needs and demand -- statistics and numerical data, human, insurance coverage -- statistics and numerical data, medically uninsured -- statistics and numerical data, pregnancy, questionnaires, socioeconomic factors, treatment outcomes, edinburgh postnatal depression scale |
| 354 | 2014 | Canadian Psychology | Bradley, S., Drapeau, M. | Graham Boeckh Foundation, Institut Nationald’Excellence en Santé et en Services Sociaux du Québec | Foundation/Association, Government | health services accessibility, mental health services, psychotherapy, financing, government, human, questionnaires, canada, male, female, surveys, summated rating scaling, dsm, professional role, psychotherapists, patient satisfaction, outcomes (health care) |
| 238 | 2014 | JAMA Psychiatry | Cummings, J. R., Wen, H., Ko, M., Druss, B. G. | National Institute of Mental Health | Government | population, ethnic groups -- statistics and numerical data, health services accessibility -- statistics and numerical data, medicaid -- statistics and numerical data, substance use rehabilitation programs -- statistics and numerical data, substance use disorders -- therapy, human, socioeconomic factors, united states |
| 362 | 2014 | Graduate Thesis | Davis, K. D. | not reported | Not reported | correlation coefficients, treatment services, national problem, psychiatric sequelae, research findings, nonparametric correlation coefficients, posttraumatic stress disorder, challenges veterans face, federal government agencies, study population, biopsychosocial model, cross-sectional study, healthcare providers, rank nonparametric correlation, traumatic event, theoretical foundation, medical facilities, positive social change, seeking treatment, social variables, biological symptoms, facilities, symptoms, treatment, sequelae |
| 170 | 2014 | Military Medicine | Erbes, C. R., Stinson, R., Kuhn, E., Polusny, M., Urban, J., Hoffman, J., Ruzek, J. I., Stepnowsky, C., Thorp, S. R. | Veterans Administration, National Center for PTSD | Government, Government | veterans, health services accessibility, attitude to health, stress disorders, post-traumatic -- therapy, telemedicine -- utilization, ambulatory care, age factors, mobile applications -- utilization, young adult, psychotherapy -- methods, mental health services, aged, female, adult, middle age, aged, 80 and over, anxiety -- therapy, united states, male |
| 146 | 2014 | Health & Place | Gibson, B. A., Ghosh, D., Morano, J. P., Altice, F. L. | National Institute on Drug Abuse, National Institutes of Allergy and Infectious Diseases, Substance Abuse and Mental Health Services Agency, Gilead Sciences Foundation, Liberty Community Services, The Hunger and Homelessness Project at Yale School of Medicine, Yale-New Haven Hospital Medical Staff Fund | Government, Government, Unknown, Foundation/Association, Industry, University, University | health services accessibility/statistics & numerical data, mobile health units/statistics & numerical data, substance-related disorders/psychology, vulnerable populations/psychology, vulnerable populations/statistics & numerical data, connecticut/epidemiology, electronic health records, female, geographic information systems, health behavior, humans, male, regression analysis, substance abuse treatment centers/statistics & numerical data, substance-related disorders/epidemiology, substance-related disorders/therapy, accessibility, geographic information system, mobile medical clinic, substance use disorders, vulnerable populations |
| 423 | 2014 | Psychiatric Rehabilitation Journal | Hilton, N. Z., Turan, C. | not reported | Not reported | parenting, mental disorders -- rehabilitation, recovery, health services accessibility -- evaluation, support, psychosocial, human, survey research, ontario, attitude of health personnel, referral and consultation, community mental health services, benchmarking |
| 78 | 2014 | Journal of Health Care for the Poor & Underserved | Jones, E., Lebrun-Harris, L. A., Sripipatana, A., Ngo-Metzger, Q. | Health Resources and Services Administration | Government | health services accessibility, mental health services -- utilization, community health centers -- united states, health services needs and demand, human, united states, survey research -- united states, health care costs, multivariate analysis, secondary analysis, odds ratio, confidence intervals, health status disparities, descriptive statistics, mental status -- classification, descriptive research, mental disorders, chronic |
| 340 | 2014 | The International Journal on Drug Policy | Kao, D., Torres, L. R., Guerrero, E. G., Mauldin, R. L., Bordnick, P. S. | National Institute on Drug Abuse | Government | heroin dependence/epidemiology, mexican americans/statistics & numerical data, substance abuse treatment centers/supply & distribution, substance abuse, intravenous/epidemiology, aged, ambulatory care facilities/supply & distribution, geographic information systems, health services accessibility, heroin dependence/psychology, humans, internal-external control, logistic models, male, middle aged, residence characteristics/statistics & numerical data, spatial analysis, substance abuse, intravenous/psychology, texas/epidemiology, geography of drug treatment programs, injection heroin users, mexican americans, spatial accessibility, treatment utilization |
| 320 | 2014 | Journal of Urban Health | Kertesz, S. G., McNeil, W., Cash, J. J., Desmond, R., McGwin, G., Jr., Kelly, J., Baggett, T. P. | University of Alabama at Birmingham School of Medicine | University | delivery of health care/statistics & numerical data, health services accessibility/statistics & numerical data, health services needs and demand/statistics & numerical data, homeless persons/statistics & numerical data, needs assessment/statistics & numerical data, primary health care/statistics & numerical data, adult, cross-sectional studies, female, health care surveys, humans, male, united states |
| 380 | 2014 | Maternal & Child Health Journal | Miller, K. | not reported | Not reported | medicaid, child, disabled, health services accessibility, child health services, child, medically fragile, mental health services, specialties, medical, human, interviews, quasi-experimental studies, logistic regression, michigan, male, female, infant, newborn, child, preschool, child, adolescence |
| 244 | 2014 | International Journal of Social Psychiatry | Mulder, C. L., Ruud, T., Bahler, M., Kroon, H., Priebe, S. | none | No funding | mental disorders, chronic, quality of health care, mental health services, human, europe, structured questionnaires, descriptive statistics, exploratory research, vignettes, questionnaires, spearman's rank correlation coefficient, outpatients |
| 127 | 2014 | Substance Use & Misuse | Phillips, M., DeBeck, K., Desjarlais, T., Morrison, T., Feng, C., Kerr, T., Wood, E. | National Institutes of Health, Canadian Institutes of Health Research | Government, Government | homeless persons -- psychosocial factors -- british columbia, street drugs -- therapeutic use, substance use rehabilitation programs -- utilization, health services accessibility, human, male, female, adolescence, adult, prospective studies, british columbia, questionnaires, logistic regression, descriptive statistics, indigenous peoples -- psychosocial factors -- canada, canada, substance dependence -- therapy, funding source |
| 257 | 2014 | Behaviour Research and Therapy | Prina, A. M., Marioni, R. E., Hammond, G. C., Jones, P. B., Brayne, C., Dening, T. | Medical Research Council | Government | accessibility, anxiety, cbt, depression old age psychiatry, general practice, psychological therapies, waiting times, adolescent, adult, age factors, aged, aging, depression, england, female, health services accessibility, humans, male, mental health services, middle aged, outcome and process assessment (health care), psychotherapy, referral and consultation, time factors, young adult, health care delivery, age differences, cognitive behavior therapy, major depression |
| 183 | 2014 | Journal of Psychiatric Practice | West, J. C., Wilk, J. E., Duffy, F. F., Kuramoto-Crawford, J., Rae, D. S., Mościcki, E. K., Hoge, C. W. | Walter Reed Army Institute of Research | Government | health services accessibility -- statistics and numerical data, mental health services -- statistics and numerical data, military personnel -- statistics and numerical data, quality of health care -- statistics and numerical data, adolescence, adult, female, human, male, young adult |
| 329 | 2013 | Journal of Studies on Alcohol and Drugs | Abraham, A. J., Knudsen, H. K., Rieckmann, T., Roman, P. M. | National Institute on Drug Abuse, The Robert Wood Johnson Foundation | Government, Foundation/Association | health services accessibility/economics, healthcare disparities/economics, substance abuse treatment centers/organization & administration, substance-related disorders/rehabilitation, evidence-based practice/economics, financing, government/economics, health care surveys, humans, physicians/economics, physicians/supply & distribution, private sector/economics, regression analysis, substance abuse treatment centers/economics, substance abuse treatment centers/standards, substance-related disorders/economics, united states |
| 433 | 2013 | Graduate Thesis | Andrews, C. M., Abraham, A. J., Grogan, C. M., Westlake, M. A., Pollack, H. A., Friedmann, P. D. | not reported | Not reported | state medicaid, outpatient, substance abuse, treatment services, health reform, drug abuse, medicaid, mental health services, outpatients, trends, uninsured (health insurance) |
| 91 | 2013 | Social Science & Medicine | Archibald, M. E., Putnam Rankin, C. | not reported | Not reported | health services accessibility -- evaluation -- united states, race factors, socioeconomic factors, substance abuse -- therapy, human, united states, post hoc analysis, descriptive statistics |
| 386 | 2013 | Graduate Thesis | Ghosheh, M. R. | not reported | Not reported | counseling center directors, counseling centers, ethnic minorities, institution size, institution type, online survey, organizational cultural competence, organizational cultural competency, outreach services, traditional mental health services, colleges, competence, counseling, mental health services, mental health, minority groups |
| 377 | 2013 | Children and Youth Services Review | Morton, C. M. | National Institute on Drug Abuse | Government | child maltreatment, neighborhood effects, substance abuse, prevention, alcohol outlets |
| 463 | 2013 | The CBHSQ Report | Smith, K., Kuramoto-Crawford, J., Lynch, S. | not reported | Not reported | short report, mental health facility, treatment data, 2010, mature adults as audience, policymakers, program planners administrators and project managers, public health professionals, public officials, researchers, mental illness, access to care |
| 317 | 2013 | Drugs: Education, Prevention & Policy | Welbel, M., Matanov, A., Moskalewicz, J., Barros, H., Canavan, R., Gabor, E., Gaddini, A., Greacen, T., Kluge, U., Lorant, V., Esteban Peña, M., Schene, A. H., Soares, J. J. F., Straßmayr, C., Vondráčkov, P., Priebe, S., | DG-Sanco | Government | healthcare disparities, substance use disorders -- therapy, behavior, addictive -- therapy, health services accessibility, mental health services, human, urban areas, european union, questionnaires, special populations, multicenter studies, interviews, descriptive statistics, data analysis software, geographic factors, funding source |
| 227 | 2013 | JAMA Psychiatry | Wen, H., Cummings, J. R., Hockenberry, J. M., Gaydos, L. M., Druss, B. G. | National Institute of Mental Health | Government | insurance, health -- legislation and jurisprudence, mental health -- legislation and jurisprudence, substance use rehabilitation programs -- legislation and jurisprudence, substance use disorders -- therapy, surveys, human, insurance, health -- statistics and numerical data, mental health -- statistics and numerical data, patient protection and affordable care act -- legislation and jurisprudence, patient protection and affordable care act -- statistics and numerical data, substance use rehabilitation programs -- statistics and numerical data, united states, substance abuse and mental health services administration -- legislation and jurisprudence, substance abuse and mental health services administration -- statistics and numerical data, substance abuse and mental health services administration -- standards |
| 96 | 2012 | Rural & Remote Health | Black, G., Roberts, R. M., Li-Leng, T. | not reported | Not reported | depression -- diagnosis -- in adolescence, rural areas, sex factors, health services accessibility, mental health services, depression -- therapy -- in adolescence, human, male, female, adolescence, south australia, descriptive statistics, questionnaires, scales, socioeconomic factors, data analysis software, chi square test, mann-whitney u test, unpaired t-tests |
| 333 | 2012 | Children and Youth Services Review | Cheng, T. C., Lo, C. C. | not reported | Not reported | racial disparities, child welfare, access to services, worker-client engagement |
| 453 | 2012 | Health Services Research | Fields, D., Roman, P. M., Blum, T. C. | National Institute on Drug Abuse | Government | outcome and process assessment (health care), patient-centered care/organization & administration, substance abuse treatment centers/organization & administration, substance-related disorders/rehabilitation, total quality management/organization & administration, cross-sectional studies, factor analysis, statistical, health care surveys, health resources, humans, models, theoretical, quality indicators, health care, united states |
| 318 | 2012 | BMC Medical Informatics And Decision Making | Kenicer, D., McClay, C. A., Williams, C. | University of Glasgow | University | health policy, professional-patient relations, therapy, computer-assisted, administrative personnel/psychology, cognitive behavioral therapy/instrumentation, access to information, computers/statistics & numerical data, health promotion, health surveys, humans, information dissemination/methods, information systems/standards, national health programs, scotland, software design, surveys and questionnaires, workforce |
| 184 | 2012 | Bulletin of the World Health Organization | Lora, A., Kohn, R., Levav, I., McBain, R., Morris, J., Saxena, S. | not reported | Not reported | developing countries/economics, health services accessibility/statistics & numerical data, health services needs and demand/statistics & numerical data, mental health services/statistics & numerical data, schizophrenia/therapy, developing countries/statistics & numerical data, health care surveys, humans, income, mental health services/economics, multivariate analysis, prevalence, schizophrenia/drug therapy, schizophrenia/economics, schizophrenia/epidemiology, statistics, nonparametric, united states/epidemiology, world health organization |
| 359 | 2012 | Plos Medicine | McBain, R., Norton, D. J., Morris, J., Yasamy, M. T., Betancourt, T. S. | not reported | Not reported | mental health services, psychotropic drugs, world health organization, health services accessibility/economics, architectural accessibility/economics, cross-sectional studies, developing countries/economics, health services accessibility/organization & administration, human rights/economics, humans, mental disorders/diagnosis, mental disorders/therapy |
| 10 | 2012 | Health & Place | Metraux, S., Brusilovskiy, E., Prvu-Bettger, J. A., Irene Wong, Y. L., Salzer, M. S. | National Institute on Disability and Rehabilitation Research | Government | health services accessibility, community networks/supply & distribution, mental disorders/diagnosis, adult, female, geography, humans, male, mental disorders/epidemiology, mental health services/supply & distribution, middle aged, philadelphia/epidemiology, severity of illness index |
| 92 | 2012 | Spatial and Spatio-Temporal Epidemiology | Ngamini Ngui, A., Vanasse, A. | not reported | Not reported | geographic information systems, spatial analysis, health services accessibility/statistics & numerical data, mental health services/supply & distribution, urban health services/supply & distribution, catchment area (health), healthcare disparities/statistics & numerical data, humans, quebec |
| 348 | 2012 | Japanese Journal Of Clinical Oncology | Ogawa, A., Nouno, J., Shirai, Y., Shibayama, O., Kondo, K., Yokoo, M., Takei, H., Koga, H., Fujisawa, D., Shimizu, K., Uchitomi, Y. | Cancer Foundation, Japanese Ministry of Health, Labour and Welfare | Foundation/Association, Government | cancer care facilities, palliative care, patient care team, psychiatry, referral and consultation, neoplasms/psychology, data collection, humans, japan, neoplasms/therapy, psychosomatic medicine |
| 182 | 2012 | The Journal of Behavioral Health Services & Research | Steinman, K. J., Kelleher, K., Dembe, A. E., Wickizer, T. M., Hemming, T. | not reported | Not reported | appointments and schedules, health services accessibility, mental health services/statistics & numerical data, adolescent, adult, child, consumer behavior, female, health care surveys, health services research, humans, insurance, health, male, medicaid, ohio, program evaluation/methods, telephone, united states, waiting lists |
| 112 | 2011 | Military Medicine | Avery, G. H., Wadsworth, S. M. | not reported | Not reported | article, health care delivery, health insurance, human, insurance, mental health service, pilot study, soldier, united states |
| 38 | 2011 | The Journal of Behavioral Health Services & Research | Chuang, E., Wells, R., Alexander, J. A. | National Institute on Drug Abuse | Government | health services accessibility/economics, managed care programs/economics, substance abuse treatment centers/economics, substance-related disorders/economics, health care surveys, health services research, humans, longitudinal studies, medicaid/economics, outpatients, substance-related disorders/therapy, united states |
| 280 | 2011 | Canadian Journal of Psychiatry | Goldner, E. M., Jones, W., Fang, M. L. | Michael Smith Foundation | Foundation/Association | health services accessibility -- statistics and numerical data, mental health services -- statistics and numerical data, urban population, waiting lists, adult, british columbia, cognitive therapy -- statistics and numerical data, female, surveys, male, psychiatry -- statistics and numerical data, referral and consultation -- statistics and numerical data, time factors |
| 266 | 2011 | Substance Abuse Treatment, Prevention, And Policy | Guerrero, E. G., Pan, K. B., Curtis, A., Lizano, E. L. | not reported | Not reported | geographic information systems/statistics & numerical data, health services accessibility/statistics & numerical data, hispanic americans/statistics & numerical data, substance abuse treatment centers/statistics & numerical data, california, databases, factual, humans, language, los angeles, maps as topic, substance abuse treatment centers/organization & administration |
| 316 | 2011 | Drug & Alcohol Dependence | Knudsen, H. K., Studts, J. L. | National Institute on Drug Abuse | Government | smoking cessation, nicotine replacement therapy, substance use disorder treatment |
| 364 | 2010 | Family Practice | Bjertnaes, O. A., Garratt, A., Ruud, T., Hunskaar, S. | Norwegian Knowledge Centre for the Health Services | Government | community mental health centers/statistics & numerical data, general practitioners/statistics & numerical data, health services accessibility/statistics & numerical data, adult, attitude of health personnel, community mental health centers/standards, cross-sectional studies, factor analysis, statistical, general practitioners/psychology, health care surveys/standards, health care surveys/statistics & numerical data, humans, norway, psychometrics, regression analysis, reproducibility of results, surveys and questionnaires |
| 242 | 2010 | Graduate Thesis | Hu, A. D. S. | not reported | Not reported | substance abuse, mental health treatment, moloka'i, transformation suggestions, drug abuse, mental disorders, mental health, mental health services |
| 87 | 2010 | Bulletin of the World Health Organization | Kruk, M. E., Rockers, P. C., Williams, E. H., Varpilah, S. T., Macauley, R., Saydee, G., Galea, S. | McNerney Grant | University | health services accessibility -- evaluation -- liberia, health services -- evaluation -- liberia, adult, child, counseling, descriptive statistics, educational status, emergency care, female, focus groups, funding source, hiv infections -- diagnosis, human, interviews, liberia, male, obstetric care, pediatric care, pregnancy, surveys |
| 108 | 2010 | Psychiatric Services | McCarthy, J. F., Valenstein, M., Zivin, K., Zeber, J. E., Kilbourne, A. M. | Veterans Administration | Government | bipolar disorder -- therapy, community mental health services -- utilization, health services accessibility, veterans -- psychosocial factors, adult, aged, female, human, male, middle age, questionnaires, regression, united states, young adult |
| 360 | 2010 | The Journal of Clinical Psychiatry | Mościcki, E. K., West, J. C., Rae, D. S., Rubio-Stipec, M., Wilk, J. E., Regier, D. A. | American Psychiatric Association Foundation | Foundation/Association | medicaid, medicare, suicidal ideation, health services accessibility/statistics & numerical data, medication adherence/statistics & numerical data, mental disorders/drug therapy, prescription drugs/administration & dosage, adult, aged, aged, 80 and over, cross-sectional studies, eligibility determination/methods, female, humans, insurance, psychiatric, male, medicare part d/statistics & numerical data, mental disorders/psychology, middle aged, prescription drugs/therapeutic use, risk factors, self administration/statistics & numerical data, united states |
| 449 | 2010 | Drugs: Education, Prevention & Policy | Myers, B., Parry, C. D. H. | South African Department of Social Development | Government | substance abuse -- complications, hiv infections -- prevention and control, south africa, hiv infections -- risk factors, male, health services accessibility -- evaluation, hiv infections -- therapy, human, hiv infections -- epidemiology, cross sectional studies, data collection, questionnaires, audit -- methods, data analysis -- methods, data analysis software, chi square test |
| 152 | 2010 | Journal for Healthcare Quality | Watkins, K. E., Keyser, D. J., Smith, B., Mannle, T. E., Kivlahan, D. R., Paddock, S. M., Mattox, T., Horvitz-Lennon, M., Pincus, H. A. | Veterans Administration, Irving Institute for Clinical and Translational Research at Columbia University, National Center for Research Resources, Mental Health Therapeutics CERT at Rutgers, Agency for Healthcare Research and Quality | Government, University, Government, University, Government | mental health services -- evaluation, united states department of veterans affairs, quality improvement, health services accessibility, quality of health care -- evaluation, outcomes (health care), human, united states, models, theoretical, conceptual framework, surveys, record review, veterans, professional practice, evidence-based, funding source, education, continuing (credit) |
| 313 | 2009 | Psychiatric Services | Huskamp, H. A., West, J. C., Rae, D. S., Rubio-Stipec, M., Regier, D. A., Frank, R. G. | National Institute of Mental Health | Government | mentally ill patients, access to medication, use of intensive mental health services, medicaid, medicare, adult, aged, critical care, eligibility determination, female, health care surveys, health services accessibility, humans, male, medicare part d, mental disorders, middle aged, united states, drug therapy, mental health services, patients |
| 241 | 2009 | Graduate Thesis | Johnson, A. O. | not reported | Not reported | geographic availability, substance abuse treatment facilities, rural veterans, armed forces, drug abuse, drug therapy, geography, treatment facilities, substance use treatment, army personnel, health care psychology |
| 390 | 2009 | Journal of Substance Abuse Treatment | Knudsen, H. K., Studts, J. L. | The Robert Wood Johnson Foundation | Foundation/Association | adolescent, article, child health care, drug dependence treatment, funding, health care quality, health program, human, manager, medical research, outpatient care, priority journal |
| 369 | 2009 | The Journal of Behavioral Health Services & Research | Merrick, E. L., Horgan, C. M., Garnick, D. W., Reif, S., Stewart, M. T. | National Institute on Alcohol Abuse and Alcoholism | Government | article, behavior disorder, health service, health survey, human, medical information, private health insurance, standardization, substance abuse |
| 334 | 2009 | Journal of Nervous and Mental Disease | Perron, B. E., Jarman, C. N., Kilbourne, A. M. | Veterans Administration, The Curtis Center of the University of Michigan | Government, University | complementary and alternative medicine, bipolar disorder, service access, barriers to treatment, health care delivery |
| 25 | 2009 | Annals of Emergency Medicine | Rhodes, K. V., Vieth, T. L., Kushner, H., Levy, H., Asplin, B. R. | National Institute of Mental Health, Agency for Healthcare Research and Quality | Government, Government | appointments and schedules, depression -- therapy, health services accessibility, insurance coverage, mental health services -- utilization, referral and consultation, telephone, continuity of patient care -- economics, depression -- epidemiology, emergency service, health services research, medicaid, mental health services -- economics, time factors, united states, human |
| 247 | 2009 | Community Mental Health Journal | Scheyett, A., Vaughn, J., Taylor, M. F. | North Carolina Governor’s Advocacy Council for Persons with Disabilities | Government | forensic psychiatry/methods, forensic psychiatry/statistics & numerical data, health services accessibility/statistics & numerical data, mass screening/methods, mental disorders/diagnosis, mental disorders/therapy, mental health services/statistics & numerical data, prisons/statistics & numerical data, adult, female, humans, male, mental disorders/epidemiology, north carolina/epidemiology, severity of illness index, young adult |
| 260 | 2009 | Alcoholism Treatment Quarterly | West, S. L., Graham, C. W., Cifu, D. X. | not reported | Not reported | alcoholism -- therapy, architectural accessibility, disabled, health facilities, health services accessibility, substance abuse -- therapy, chi square test, descriptive statistics, exploratory research, great britain, pilot studies, self report, sign language, stratified random sample, surveys, human |
| 375 | 2009 | Alcoholism Treatment Quarterly | West, S. L., Graham, C. W., Cifu, D. X., , , , , , , , , , , , , , | not reported | Not reported | alcoholism -- therapy, architectural accessibility, disabled, health facilities, health services accessibility, substance abuse -- therapy, analysis of variance, brain injuries, chi square test, descriptive statistics, post hoc analysis, random sample, spinal cord injuries, surveys, treatment refusal, type i error, united states, human |
| 414 | 2009 | American Journal of Public Health | Zeber, J. E., Copeland, L. A., McCarthy, J. F., Bauer, M. S., Kilbourne, A. M. | Veterans Administration | Government | bipolar disorder -- rehabilitation, health services accessibility, mental health services -- utilization, veterans, adult, age factors, correlational studies, cross sectional studies, diagnosis, dual (psychiatry), female, funding source, health care costs, health services needs and demand, homeless persons, interviews, logistic regression, male, odds ratio, pennsylvania, primary health care, professional-patient relations, self report, specialization, human |
| 398 | 2008 | Psychiatric Services | Barry, C. L., Venkatesh, M., Busch, S. H. | Connecticut Office of the Healthare Advocate | Government | health services accessibility, mental health services, connecticut, health maintenance organizations, humans, interviews as topic, logistic models |
| 221 | 2008 | General Hospital Psychiatry | Pomerantz, A., Cole, B. H., Watts, B. V., Weeks, W. B. | not reported | Not reported | health care delivery, integrated -- administration, health and welfare planning -- administration, health services accessibility -- administration, mental health services -- administration, primary health care -- administration, veterans, organizational efficiency, surveys, human, patient satisfaction, quality assurance -- administration, referral and consultation -- administration, rural population, united states, waiting lists |
| 83 | 2008 | Psychiatric Services | Salvador-Carulla, L., Saldivia, S., Martinez-Leal, R., Vicente, B., Garcia-Alonso, C., Grandon, P., Haro, J. M. | Andalusian Research Plan, Network of Primary Care Research in Spain, Spanish International Cooperation Agency, Proyecto FONDECYT, Proyecto Universidad del Desarrollo in Chile | Government, Industry, Government, Government, University | catchment health areas, mental health service availability, chile, spain, meso-level comparison, health care utilization, urban areas, ambulances, catchment area (health), humans, mental disorders, mental health services, prospective studies, psychology, rural population, socioeconomic factors, surveys and questionnaires, urban population, urban environments, countries |
| 14 | 2008 | Social Work | Ting, L., Jacobson, J. M., Sanders, S. | not reported | Not reported | coping, social work, psychiatric -- psychosocial factors, social workers -- psychosocial factors, suicide -- psychosocial factors, support, psychosocial, adult, chi square test, coefficient alpha, correlation coefficient, empirical research, female, male, middle age, psychological tests, qualitative studies, sex factors, social networks, surveys, t-tests, human |
| 277 | 2007 | Substance Use & Misuse | Cochran, B. N., Peavy, K. M., Robohm, J. S. | not reported | Not reported | program development, homosexuality/statistics & numerical data, mental health services/supply & distribution, patient acceptance of health care/statistics & numerical data, substance-related disorders/rehabilitation, female, health services needs and demand, hospitalization, humans, male, program evaluation, puerto rico/epidemiology, substance-related disorders/prevention & control, treatment outcome, united states/epidemiology |
| 109 | 2007 | The Journal of Mental Health Policy and Economics | Fang, H., Rizzo, J. A. | not reported | Not reported | psychiatry, continuity of patient care/organization & administration, health services/statistics & numerical data, health services accessibility/organization & administration, demography, humans, insurance coverage/statistics & numerical data, insurance, health/statistics & numerical data, medicine, mental health services/statistics & numerical data, referral and consultation/organization & administration, sex factors, specialization |
| 105 | 2007 | Journal of Rural Health | Hartley, D., Ziller, E. C., Loux, S. L., Gale, J. A., Lambert, D., Yousefian, A. E. | Office of Rural Health Policy | Government | community mental health services -- utilization, emergency service -- utilization, health services accessibility, hospitals, rural -- utilization, mental disorders -- epidemiology, adolescence, adult, affective disorders, aged, anxiety disorders, chi square test, descriptive statistics, exploratory research, female, funding source, health services research, male, mental disorders -- classification, middle age, needs assessment, p-value, pilot studies, prospective studies, psychotic disorders, substance use disorders, survey research, united states, human |
| 33 | 2007 | Psychiatric Services | Knudsen, H. K., Ducharme, L. J., Roman, P. M. | National Institute on Drug Abuse | Government | racial and ethnic disparities, selective serotonin reuptake inhibitors, substance abuse treatment, african americans, ambulatory care facilities, drug therapy, european continental ancestry group, hispanic americans, humans, mental health services, prejudice, serotonin uptake inhibitors, substance-related disorders, surveys and questionnaires, united states, racial and ethnic differences, serotonin reuptake inhibitors, substance use treatment, drug abuse |
| 355 | 2007 | Administration and Policy in Mental Health and Mental Health Services Research | Masland, M. C., Snowden, L. R., Wallace, N. T. | not reported | Not reported | health services accessibility, mental health services, eligibility determination/organization & administration, managed care programs/organization & administration, medicaid/organization & administration, california, health care surveys, humans |
| 365 | 2007 | Health Services Research | McCarthy, J. F., Blow, F. C., Valenstein, M., Fischer, E. P., Owen, R. R., Barry, K. L., Hudson, T. J., Ignacio, R. V. | Veterans Administration | Government | health services accessibility/statistics & numerical data, hospitals, veterans/statistics & numerical data, mental disorders/therapy, mental health services/statistics & numerical data, patient acceptance of health care/psychology, veterans/psychology, bipolar disorder/therapy, continuity of patient care, female, geography, humans, male, mental disorders/epidemiology, middle aged, proportional hazards models, psychological tests, schizophrenia/therapy, sickness impact profile, united states/epidemiology, united states department of veterans affairs |
| 32 | 2007 | Australian Journal of Rural Health | Morley, B., Pirkis, J., Naccarella, L., Kohn, F., Blashki, G., Burgess, P. | Australian Government Department of Health and Aging | Government | health services accessibility -- australia, mental health services -- australia, outcomes (health care), allied health personnel, australia, case studies, chi square test, female, funding source, male, minimum data set, physicians, family, post hoc analysis, repeated measures, rural areas, surveys, two-way analysis of variance, human |
| 264 | 2007 | Learning Disability Practice | Palmer, H. | not reported | Not reported | learning disorders -- complications, mental disorders -- therapy, psychotherapy -- utilization, adult, crime, england, health resource utilization, health services accessibility, mental disorders -- epidemiology, professional competence, professional-client relations, psychotherapists, specialization, survey research, thematic analysis, human |
| 434 | 2007 | The American Journal of Drug and Alcohol Abuse | Sorensen, J. L., Guydish, J., Zilavy, P., Davis, T. B., Gleghorn, A., Jacoby, M., Sears, C. | National Institute on Drug Abuse, Center for Substance Abuse Treatment | Government, Government | health policy, health services accessibility, waiting lists, substance abuse treatment centers/organization & administration, substance-related disorders/rehabilitation, humans, regression analysis, san francisco, substance abuse treatment centers/economics, substance abuse treatment centers/statistics & numerical data |
| 410 | 2006 | The Journal of Behavioral Health Services & Research | Campbell, C. I., Alexander, J. A. | National Institute on Drug Abuse | Government | ambulatory care, substance abuse treatment centers/trends, female, health services research, humans, reproductive medicine, social work, united states |
| 356 | 2006 | Suicide & Life-Threatening Behavior | Cooper, S. L., Lezotte, D., Jacobellis, J., Diguiseppi, C. | Centers for Disease Control and Prevention | Government | mental health services/supply & distribution, suicide, attempted/psychology, adolescent, adult, aged, colorado, crisis intervention, female, humans, male, middle aged, suicide/prevention & control |
| 419 | 2006 | Mental Health Practice | Crowley, J., Davis, D. A., Steadman, P. | Research and Development Office of Oxleas NHS Trust | Government | community mental health services, information systems -- utilization, attitude of health personnel, cross sectional studies, questionnaires, random sample, survey research, wireless communications -- utilization, human |
| 455 | 2006 | Psychiatric Services | Davis, M., Geller, J. L., Hunt, B. | Substance Abuse and Mental Health Services Administration | Government | within state availability of transition services, adulthood services, child mental health systems, adult mental health systems, adolescent, adolescent health services, adult, child, child health services, community mental health services, continuity of patient care, diagnosis, dual (psychiatry), humans, mental disorders, severity of illness index, social support, substance-related disorders, surveys and questionnaires, united states, vocational guidance, mental health, mental health services, adolescent psychiatry |
| 363 | 2006 | Community Mental Health Journal | Ducharme, L. J., Knudsen, H. K., Roman, P. M. | National Institute on Drug Abuse | Government | comorbidity, health care delivery, integrated, health services accessibility, mental disorders, substance use disorders, adult, drug rehabilitation programs, female, funding source, male, surveys, human |
| 239 | 2006 | Journal of Forensic Psychiatry & Psychology | Durand, M. A., Lelliott, P., Coyle, N. | Department of Health’s National Research Programme on Forensic Mental Health R&D | Government | treatment availability, substance misuse, psychiatric care, medium secure facilities, comorbidity, alcohol problems, correctional institutions, drug abuse, mental disorders, treatment, alcohol abuse, substance use treatment |
| 175 | 2006 | Drug & Alcohol Dependence | Koch, A. L., Arfken, C. L., Schuster, C. R. | Reckitt Benckiser Pharmaceutical, Inc | Industry | buprenorphine -- therapeutic use, substance dependence -- drug therapy, substance use rehabilitation programs, confidence intervals, data analysis software, funding source, logistic regression, naltrexone -- therapeutic use, odds ratio, survey research, united states, human |
| 222 | 2006 | International Journal of Nursing Practice | Morita, T., Yamazaki, H. | not reported | Not reported | community mental health services -- japan, health services accessibility -- evaluation -- japan, psychiatric patients -- japan, support, psychosocial, community mental health nursing, convenience sample, data analysis software, descriptive statistics, factor analysis, japan, mail, multivariate analysis, pearson's correlation coefficient, questionnaires, registered nurses, rural areas, urban areas, human |
| 315 | 2006 | Annals of Family Medicine | Solberg, L. I., Crain, A. L., Sperl-Hillen, J. A. M., Hroscikoski, M. C., Engebretson, K. I., O'Connor, P. J. | The Robert Wood Johnson Foundation | Foundation/Association | improved primary care access, quality of depression care, continuum of care, adult, antidepressive agents, continuity of patient care, depressive disorder, female, health services accessibility, humans, logistic models, male, middle aged, primary health care, quality of health care, major depression, quality of care |
| 347 | 2005 | International Journal of Law and Psychiatry | Beecham, J. | not reported | Not reported | none listed |
| 418 | 2005 | Archives of Internal Medicine | Brooks, R. G., Menachemi, N., Clawson, A., Beitsch, L. | Center for Rural Health Research and Policy of the Florida State University College of Medicine | University | insurance, liability, health services accessibility/trends, physicians/supply & distribution, florida, health surveys, humans |
| 391 | 2005 | Pediatrics | Kempe, A., Beaty, B. L., Crane, L. A., Stokstad, J., Barrow, J., Belman, S., Steiner, J. F. | Rose Community Foundation, Colorado Child Health Foundation | Foundation/Association, Foundation/Association | child health services -- utilization, health services accessibility, quality of health care, state health plans -- utilization -- in infancy and childhood, bivariate statistics, child, colorado, confidence intervals, data analysis software, emergency service -- utilization -- in infancy and childhood, funding source, health services needs and demand, insurance, health -- utilization -- in infancy and childhood, interviews, logistic regression, mcnemar's test, multivariate analysis, outcomes (health care), paired t-tests, poisson distribution, program evaluation, prospective studies, random sample, summated rating scaling, human |
| 301 | 2005 | Psychiatric Services | Kimerling, R., Baumrind, N. | not reported | Not reported | health services accessibility, mental health services, special populations, women, california, chi square test, confidence intervals, cross sectional studies, ethnic groups, female, interviews, multivariate analysis, odds ratio, p-value, probability sample, questionnaires, human |
| 382 | 2005 | South African Psychiatry Review | Myers, B., Parry, C. D. H. | not reported | Not reported | substance abuse treatment, blacks, health service accessibility, treatment outcomes, treatment retention, client characteristics, service delivery, treatment facilities, health care utilization, substance use treatment, drug abuse, treatment compliance, alcohol treatment |
| 75 | 2005 | Health Affairs | Reschovsky, J. D., Staiti, A. B. | Center for Studying Health System Change | Industry | health services accessibility, quality of health care, rural health, clinical competence, cluster sample, female, health care costs, health services needs and demand, health services -- utilization, insurance, health, interviews, male, physicians -- manpower, urban health, funding source, human |
| 196 | 2005 | Scandinavian Journal of Urology and Nephrology | Valdimarsdóttir, U., Helgason, A. R., Fürst, C. J., Adolfsson, J., Steineck, G. | Swedish Cancer Society, Stockholm Cancer Foundation | Foundation/Association, Foundation/Association | adult, aged, article, bereavement, bladder cancer, economic aspect, education, female, follow up, health care access, health care quality, human, major clinical study, mental health, priority journal, prostate cancer, questionnaire, religion, support group, sweden, urinary tract cancer, widow |
| 206 | 2005 | Psychiatric Services | Wilk, J. E., West, J. C., Narrow, W. E., Rae, D. S., Regier, D. A. | American Psychiatric Association Foundation, Center for Substance Abuse Treatment, Center for Mental Health Services, John D. and Catherine T. MacArthur Foundation | Foundation/Association, Government, Government, Foundation/Association | health services accessibility, managed care programs, mental disorders -- therapy, mental health services -- utilization, psychiatry -- methods, public health administration, confidence intervals, descriptive statistics, female, funding source, male, middle age, random sample, surveys, united states, human |
| 282 | 2004 | Archives of Internal Medicine | Brooks, R. G., Menachemi, N., Hughes, C., Clawson, A. | Florida Department of Health | Government | needs assessment, health services accessibility/trends, insurance, liability/economics, liability, legal/economics, rural health services/trends, adult, aged, aged, 80 and over, female, florida, health care reform, health care surveys, humans, insurance, liability/standards, male, medicine, middle aged, rural health services/supply & distribution, specialization |
| 392 | 2004 | Archives of Pediatrics & Adolescent Medicine | Johnson, K. C., Klesges, L. M., Somes, G. W., Coday, M. C., DeBon, M. | Parnership for Women's and Children's Health | Mix | nonprescription drugs, smoking cessation/methods, commerce/statistics & numerical data, adolescent, female, humans, male, tennessee |
| 45 | 2004 | Psychiatric Services | Knudsen, H. K., Roman, P. M., Ducharme, L. J. | National Institute on Drug Abuse, National Institute on Alcohol Abuse and Alcoholism | Government, Government | psychiatric programs, private substance abuse treatment centers, mental disorders, integrated service delivery, quality-of-care, health facilities, proprietary, health services research, humans, privacy, psychiatric nursing, substance abuse treatment centers, united states, comorbidity, drug abuse, health care services, psychiatric units, drug abuse prevention, interdisciplinary treatment approach, quality of care |
| 442 | 2004 | The Australian and New Zealand Journal of Psychiatry | Murray, G., Judd, F., Jackson, H., Fraser, C., Komiti, A., Hodgins, G., Pattison, P., Humphreys, J., Robins, G. | beyondblue: The National Depression Initiative | Industry | health services accessibility/statistics & numerical data, mental disorders/epidemiology, mental health services/supply & distribution, rural health services/supply & distribution, rural population/statistics & numerical data, australia/epidemiology, female, humans, male, middle aged, surveys and questionnaires |
| 72 | 2003 | Urban Affairs Review | Allard, S. W., Rosen, D., Tolman, R. M. | HHS Office of the Assistant Secretary for Planning and Evaluation, National Institute of Mental Health | Government, Government | welfare, social services, spatial access |
| 337 | 2003 | Health Services Research | Friedmann, P. D., Lemon, S. C., Stein, M. D., D'Aunno, T. A. | National Institute on Drug Abuse | Government | methadone, article, attitude, drug dependence treatment, health care access, health center, health insurance, health service, health survey, hospital admission, managed care, methadone treatment, organization, outpatient care, poverty, substance abuse, united states |
| 443 | 2003 | American Annals of the Deaf | Munro-Ludders, B., Simpatico, T., Zvetina, D. | not reported | Not reported | consumer, data analysis, follow up, health care access, health service, health survey, hearing impairment, human, mental health, practice guideline, psychiatry, public health, review, standard, telephone, united states |
| 6 | 2003 | Psychology of Addictive Behaviors | Rosenberg, H., Phillips, K. T. | not reported | Not reported | attitude of health personnel, harm reduction, health services accessibility, substance abuse treatment centers, substance-related disorders/therapy, data collection, humans, substance-related disorders/psychology, united states |
| 261 | 2003 | The American Journal on Addictions | Rosenheck, R., Leslie, D., Woody, G. | not reported | Not reported | health services accessibility, veterans, heroin dependence/economics, heroin dependence/rehabilitation, methadone/economics, methadone/therapeutic use, narcotics/economics, narcotics/therapeutic use, united states department of veterans affairs/economics, financial management, financing, government, health care costs/statistics & numerical data, health services/statistics & numerical data, hospitals, veterans/economics, humans, medical audit, mental health services/economics, mental health services/statistics & numerical data, united states, united states department of veterans affairs/statistics & numerical data |
| 58 | 2003 | Psychiatric Bulletin | Simpson, C., de Silva, P. | not reported | Not reported | old age, psychiatry services, team referral, mental health services, quality, accesibility, models, psychiatry, psychological assessment, quality of care, teams, gerontology, interdisciplinary treatment approach, mental disorders |
| 21 | 2003 | The Journal of Behavioral Health Services & Research | Slade, E. P. | National Institute of Mental Health | Government | adolescent health services/supply & distribution, health services accessibility/statistics & numerical data, mental health services/supply & distribution, school health services/organization & administration, adolescent, adolescent health services/statistics & numerical data, counseling/statistics & numerical data, counseling/supply & distribution, health care surveys, humans, longitudinal studies, medicaid, mental health services/statistics & numerical data, multivariate analysis, probability, school health services/statistics & numerical data, substance-related disorders/therapy, united states |
| 310 | 2003 | General Hospital Psychiatry | Van Voorhees, B. W., Wang, N., Ford, D. E. | National Research Service Award | Government | health services accessibility -- administration, managed care programs -- administration, mental health services, physicians, primary health care, attitude, chi square test, confidence intervals, correlation coefficient, cross sectional studies, descriptive statistics, female, male, middle age, multiple regression, p-value, patient satisfaction, pearson's correlation coefficient, physician-patient relations, quality of health care, questionnaires, referral and consultation, funding source, united states, human |
| 208 | 2002 | Supportive Care in Cancer | Curry, C., Cossich, T., Matthews, J. P., Beresford, J., McLachlan, S. A. | Commonwealth of Australia, State Government of Victoria | Government, Government | outpatient clinics, hospital, mental health services/statistics & numerical data, neoplasms/psychology, referral and consultation/statistics & numerical data, aged, australia, female, health services accessibility, humans, outpatients/psychology |
| 159 | 2002 | Journal of Applied Gerontology | Fortney, J. C., Chumbler, N., Cody, M., Beck, C. | Alzheimer's Association, Veterans Administration | Foundation/Association, Government | health services accessibility -- in old age, health resource utilization -- in old age, home health care -- utilization -- in old age, cross sectional studies, arkansas, interviews, self report, descriptive statistics, functional status -- in old age, geriatric functional assessment, pearson's correlation coefficient, logistic regression, odds ratio, p-value, aged, aged, 80 and over, male, female, funding source, human |
| 243 | 2002 | Journal Of General Internal Medicine | Grembowski, D. E., Martin, D., Patrick, D. L., Diehr, P., Katon, W., Williams, B., Engelberg, R., Novak, L., Dickstein, D., Deyo, R., Goldberg, H. I. | Agency for Healthcare Research and Quality | Government | outcome assessment (health care), depressive disorder/therapy, health services accessibility/organization & administration, managed care programs/standards, mental health services/statistics & numerical data, primary health care/organization & administration, referral and consultation/standards, adolescent, adult, aged, cohort studies, depressive disorder/diagnosis, female, humans, interprofessional relations, male, managed care programs/trends, middle aged, program evaluation, prospective studies, referral and consultation/trends, washington |
| 262 | 2002 | Addiction | Rosenberg, H., Melville, J., McLean, P. C. | not reported | Not reported | attitude of health personnel, health services accessibility, substance abuse treatment centers, substance-related disorders/drug therapy, humans, state medicine, surveys and questionnaires, united kingdom |
| 115 | 2002 | Topics in Spinal Cord Injury Rehabilitation | Voss, C. P., Cesar, K. W., Tymus, T., Fiedler, I. G. | National nstitute on Disability and Rehabilitation Research | Government | spinal cord injuries -- rehabilitation, disabled, architectural accessibility, substance use rehabilitation programs, rehabilitation centers, funding source, americans with disabilities act -- standards, pilot studies, surveys, interviews, health facility administrators, wheelchairs, health facility environment, organizational compliance, data analysis, statistical, midwestern united states, human |
| 256 | 2002 | Graduate Thesis | Waliski, A. D. | Baum Charitable Foundation, Northwest Arkansas Rape Crisis. Inc | Foundation/Association, Industry | sexual assault agencies, reporting, male victims, human males, mental health services, sexual abuse |
| 252 | 2002 | Military Medicine | Wooten, A. F. | not reported | Not reported | mental health care |
| 145 | 2001 | Mental Health Aspects of Developmental Disabilities | Kim, S. H., Cooker, P. G. | not reported | Not reported | community mental health services, developmental disabilities, psychiatric disorders, pharmacotherapy, mental retardation, hospitalization, intellectual disability, psychotropic medication, drug therapy, mental disorders, psychopharmacology, quality of services |
| 295 | 1999 | Archives of Pediatrics & Adolescent Medicine | Kaplan, D. W., Brindis, C. D., Phibbs, S. L., Melinkovich, P., Naylor, K., Ahlstrand, K. | The Carnegie Corporation | Foundation/Association | health services accessibility, school health services -- utilization, mental health services -- utilization, funding source, health status, medically uninsured, patient satisfaction, school health services -- administration, socioeconomic factors, schools, elementary, retrospective design, comparative studies, multiple regression, multiple logistic regression, kruskal-wallis test, chi square test, child, preschool, child, adolescence, odds ratio, confidence intervals, questionnaires, human |
| 234 | 1999 | Graduate Thesis | McClelland, D. E. | not reported | Not reported | availability & utilization of psychosocial & emotional support services for burn survivors & their families at major burn centers, directors of burn centers, burns, health care utilization, mental health services, support groups |
| 411 | 1999 | Substance Use & Misuse | Rivers, J. E., Komaroff, E., Kibort, A. C. | National Institute on Drug Abuse | Government | community health services/statistics & numerical data, health services accessibility/statistics & numerical data, managed care programs/trends, social work/statistics & numerical data, substance abuse treatment centers/supply & distribution, substance-related disorders/therapy, attitude of health personnel, chronic disease, community health services/economics, financial support, florida, health care surveys, health services accessibility/economics, humans, rehabilitation, vocational/statistics & numerical data, rural health services/economics, rural health services/statistics & numerical data, social work/organization & administration, substance abuse treatment centers/economics, substance-related disorders/economics, urban health services/economics, urban health services/statistics & numerical data |
| 93 | 1998 | Nonprofit and Voluntary Sector Quarterly | Wolff, N., Schlesinger, M. | not reported | Not reported | none listed |
| 451 | 1997 | Journal of School Health | Heneghan, A. M., Malakoff, M. E. | not reported | Not reported | school health services, health services accessibility, health services needs and demand, preventive health care, schools, elementary, surveys, questionnaires, conceptual framework, data analysis software, chi square test, analysis of variance, p-value, rural areas, urban areas, child, united states, human |
| 216 | 1997 | Psychiatric Services | Morrissey, J., Calloway, M., Johnsen, M., Ullman, M. | not reported | Not reported | community networks -- administration, homeless persons, health care delivery, mental disorders -- complications, mental disorders -- rehabilitation, mental health services -- administration, program evaluation, interviews, descriptive statistics, community networks -- standards, health care delivery -- standards, surveys, health services accessibility, interinstitutional relations, mental health services -- standards, social welfare, united states, summated rating scaling, pearson's correlation coefficient, research instruments, coefficient alpha, internal consistency, p-value, adult, human |
| 405 | 1997 | Graduate Thesis | Russo, P. A. | not reported | Not reported | mental illness & comorbidity, access to & utilization of health care services, non-institutionalized medicare beneficiaries, comorbidity, health care utilization, health care seeking behavior, mental disorders, mental health programs, medicare |
| 440 | 1997 | Medical Care | Schlesinger, M., Dorwart, R., Hoover, C., Epstein, S. | National Institute of Mental Health | Government | competition, access, nonprofit ownership, psychiatric hospitals, innovative services |
| 293 | 1997 | Wisconsin Medical Journal | Taman, M. S., Menz, F. E. | not reported | Not reported | health services accessibility, quality of health care, mental health services/standards, rural health services/standards, adolescent, adult, aged, attitude of health personnel, child, humans, mental health services/organization & administration, minority groups, rural health services/organization & administration, wisconsin |
| 128 | 1996 | Graduate Thesis | Green, S. M. | not reported | Not reported | comparison of available mental health services & their implementation & effectiveness & perceived need for comprehensive school-based mental health care for students, school district officials, health service needs, mental health, mental health services, school counseling |
| 457 | 1994 | American Journal of Public Health | Breitbart, V., Chavkin, W., Wise, P. H. | Aaron Diamon Foundation, Ford Foundation, The Robert Wood Johnson Foundation | Foundation/Association, Foundation/Association, Foundation/Association | health services accessibility/statistics & numerical data, prenatal care/economics, substance abuse treatment centers/statistics & numerical data, child care, female, health services accessibility/trends, humans, infant, medicaid/statistics & numerical data, pregnancy, substance abuse treatment centers/organization & administration, surveys and questionnaires, united states, urban population |
| 294 | 1993 | Journal of Alcohol and Drug Education | Lottman, T. J. | not reported | Not reported | access to generic substance abuse services through chemical dependency treatment agencies, mentally retarded, drug abuse, intellectual development disorder, substance use treatment |
| 289 | 1993 | American Annals of the Deaf | McEntee, M. K. | not reported | Not reported | crisis intervention, health services accessibility, deafness/psychology, mental disorders/therapy, mental health services/statistics & numerical data, communication aids for disabled, communication disorders/rehabilitation, data collection, deafness/diagnosis, female, humans, male, mental health services/legislation & jurisprudence, psychotherapy, sign language, workforce |
| 8 | 1992 | Children's Health Care | Kaufman, K. L., Harbeck, C., Olson, R., Nitschke, R. | not reported | Not reported | family health, cancer care facilities/statistics & numerical data, health services accessibility/statistics & numerical data, mental health services/supply & distribution, neoplasms/psychology, social work/statistics & numerical data, child, cost-benefit analysis/statistics & numerical data, humans, medical oncology/statistics & numerical data, mental health services/standards, neoplasms/complications, pain/etiology, pain/prevention & control, pediatrics/statistics & numerical data, self-help groups/statistics & numerical data, surveys and questionnaires, united states |
| 192 | 1990 | Canadian Journal Of Public Health | Svenson, L. W. | not reported | Not reported | health services accessibility/standards, mental health services/standards, adolescent, adult, alberta/epidemiology, female, humans, male, mental disorders/epidemiology, mental disorders/therapy, mental health services/statistics & numerical data, program evaluation, surveys and questionnaires, waiting lists |
| 446 | 1985 | Journal of Medical Education | Borenstein, D. B. | not reported | Not reported | academic medical centers, internship and residency, mental health services, humans, mental disorders/therapy, students, medical/psychology |
| 131 | 1984 | Journal of Prison & Jail Health | Anno, B. J. | The Thrasher Research Fund, The Robert Wood Johnson Foundation | Foundation/Association, Foundation/Association | availability of health services in detention & correctional facilities, juvenile delinquents, correctional institutions, health care services, juvenile delinquency |
| 456 | 1969 | Public Health Reports | Johnson, Pe. J., Tucker, E. B., Bradbury, B. A., Spencer, F. J. | not reported | Not reported | counseling, student health services, suicide/prevention & control, adolescent, adult, humans, virginia |

# **References of extracted articles**

Abikoye, Gboyega, Elizabeth Okonkwo, and Isidore Obot. 2021. “Drug-Use Dynamics, Treatment Need and Availability of Treatment Facility: Evidence from Selected Bunks in Uyo Metropolis in Nigeria.” *Alkoholizm I Narkomania-Alcoholism and Drug Addiction* 34(2):119–30. doi: [10.5114/ain.2021.109543](https://doi.org/10.5114/ain.2021.109543).

Abraham, Amanda J, Grace Bagwell-Adams, and Jayani Jayawardhana. 2017. “Availability of Tobacco Cessation Services in Substance Use Disorder Treatment Programs: Impact of State Tobacco Control Policy.” *Addictive Behaviors* 71:12–17. doi: [10.1016/j.addbeh.2017.02.007](https://doi.org/10.1016/j.addbeh.2017.02.007).

Abraham, Amanda J., Hannah K. Knudsen, Traci Rieckmann, and Paul M. Roman. 2013. “Disparities in Access to Physicians and Medications for the Treatment of Substance Use Disorders between Publicly and Privately Funded Treatment Programs in the United States.” *Journal of Studies on Alcohol and Drugs* 74(2):258–65. doi: [10.15288/jsad.2013.74.258](https://doi.org/10.15288/jsad.2013.74.258).

Abraham, Amanda J., Traci Rieckmann, Christina M. Andrews, and Jayani Jayawardhana. 2017. “Health Insurance Enrollment and Availability of Medications for Substance Use Disorders.” *Psychiatric Services* 68(1):41–47. doi: [10.1176/appi.ps.201500470](https://doi.org/10.1176/appi.ps.201500470).

Abraham, Amanda J., and Courtney R. Yarbrough. 2021. “Availability of Medications for the Treatment of Alcohol Use Disorder in U.S. Counties, 2016-2019.” *Journal of Studies on Alcohol and Drugs* 82(6):689–99.

Abraham, Amanda J., Courtney R. Yarbrough, Samantha J. Harris, Grace Bagwell Adams, and Christina M. Andrews. 2021. “Medicaid Expansion and Availability of Opioid Medications in the Specialty Substance Use Disorder Treatment System.” *Psychiatric Services* 72(2):148–55. doi: [10.1176/appi.ps.202000049](https://doi.org/10.1176/appi.ps.202000049).

Alenezi, Ahmad F., Ahmed Aljowder, Mohamed J. Almarzooqi, Marya Alsayed, Rashed Aldoseri, Omar Alhaj, Sally Souraya, Graham Thornicroft, and Haitham Jahrami. 2021. “Translation and Validation of the Arabic Version of the Barrier to Access to Care Evaluation (BACE) Scale.” *Mental Health and Social Inclusion* 25(4):352–65. doi: [10.1108/MHSI-05-2021-0022](https://doi.org/10.1108/MHSI-05-2021-0022).

Alinsky, Rachel H., Scott E. Hadland, Pamela A. Matson, Magdalena Cerda, and Brendan Saloner. 2020. “Adolescent-Serving Addiction Treatment Facilities in the United States and the Availability of Medications for Opioid Use Disorder.” *Journal of Adolescent Health* 67(4):542–49. doi: [10.1016/j.jadohealth.2020.03.005](https://doi.org/10.1016/j.jadohealth.2020.03.005).

Allard, S. W., D. Rosen, and R. M. Tolman. 2003. “Access to Mental Health and Substance Abuse Services among Women Receiving Welfare in Detroit.” *Urban Affairs Review* 38(6):787–807. doi: [10.1177/1078087403038006002](https://doi.org/10.1177/1078087403038006002).

Alleaume, Caroline, Pierre Verger, and Patrick Peretti-Watel. 2021. “Psychological Support in General Population during the COVID-19 Lockdown in France: Needs and Access.” *Plos One* 16(5):e0251707. doi: [10.1371/journal.pone.0251707](https://doi.org/10.1371/journal.pone.0251707).

Andrews, Christina. 2013. “State Medicaid Coverage and the Availability of Medicaid-Covered Outpatient Substance Abuse Treatment Services: Current Trends and Implications for Expansion under Health Reform.” ProQuest Information & Learning.

Andrews, Christina M., Amanda J. Abraham, Colleen M. Grogan, Melissa A. Westlake, Harold A. Pollack, and Peter D. Friedmann. 2019. “Impact of Medicaid Restrictions on Availability of Buprenorphine in Addiction Treatment Programs.” *American Journal of Public Health* 109(3):434–36. doi: [10.2105/AJPH.2018.304856](https://doi.org/10.2105/AJPH.2018.304856).

Anno, B. Jaye. 1984. “The Availability of Health Services for Juvenile Offenders: Preliminary Results of a National Survey.” *Journal of Prison & Jail Health* 4(2):77–90.

Antoine, Denis, Sean Heffernan, Amina Chaudhry, Van King, and Eric C. Strain. 2016. “Age and Gender Considerations for Technology-Assisted Delivery of Therapy for Substance Use Disorder Treatment: A Patient Survey of Access to Electronic Devices.” *Addictive Disorders & Their Treatment* 15(4):149–56. doi: [10.1097/ADT.0000000000000088](https://doi.org/10.1097/ADT.0000000000000088).

Archibald, Matthew E., and Caddie Putnam Rankin. 2013. “A Spatial Analysis of Community Disadvantage and Access to Healthcare Services in the U.S.” *Social Science & Medicine* 90:11–23. doi: [10.1016/j.socscimed.2013.04.023](https://doi.org/10.1016/j.socscimed.2013.04.023).

Arega, Melaku A., Edward Christopher Dee, Vinayak Muralidhar, Paul L. Nguyen, Idalid Franco, Brandon A. Mahal, and Nina N. Sanford. 2021. “Psychological Distress and Access to Mental Health Services Among Cancer Survivors: A National Health Interview Survey Analysis.” *Journal of General Internal Medicine* 36(10):3243–45. doi: [10.1007/s11606-020-06204-3](https://doi.org/10.1007/s11606-020-06204-3).

Argento, Elena, Shira Goldenberg, Melissa Braschel, Sylvia Machat, Steffanie A. Strathdee, and Kate Shannon. 2020. “The Impact of End-Demand Legislation on Sex Workers’ Access to Health and Sex Worker-Led Services: A Community-Based Prospective Cohort Study in Canada.” *PloS One* 15(4):e0225783. doi: [10.1371/journal.pone.0225783](https://doi.org/10.1371/journal.pone.0225783).

Avery, George H., and Shelley M. MacDermid Wadsworth. 2011. “Access to Mental Health Services for Active Duty and National Guard TRICARE Enrollees in Indiana.” *Military Medicine* 176(3):261–64. doi: [10.7205/MILMED-D-10-00219](https://doi.org/10.7205/MILMED-D-10-00219).

Baćak, Valerio, and Greg Ridgeway. 2018. “Availability of Health-Related Programs in Private and Public Prisons.” *Journal of Correctional Health Care* 24(1):62–70. doi: [10.1177/1078345817728078](https://doi.org/10.1177/1078345817728078).

Barry, Colleen L., Mohini Venkatesh, and Susan H. Busch. 2008. “Assessing New Patient Access to Mental Health Providers in HMO Networks.” *Psychiatric Services (Washington, D.C.)* 59(12):1413–18. doi: [10.1176/appi.ps.59.12.1413](https://doi.org/10.1176/appi.ps.59.12.1413).

Barry, Declan T., Tera Fazzino, Emily Necrason, Joel Ginn, Lynn E. Fiellin, David A. Fiellin, and Brent A. Moore. 2016. “The Availability of Ancillary Counseling in the Practices of Physicians Prescribing Buprenorphine.” *Journal of Addiction Medicine* 10(5):352–56. doi: [10.1097/ADM.0000000000000247](https://doi.org/10.1097/ADM.0000000000000247).

Beecham, J. 2005. “Access to Mental Health Supports in England: Crisis Resolution Teams and Day Services.” *International Journal of Law and Psychiatry* 28(5):574–87. doi: [10.1016/j.ijlp.2005.08.009](https://doi.org/10.1016/j.ijlp.2005.08.009).

Benjamen, Joseph, Vincent Girard, Shabana Jamani, Olivia Magwood, Tim Holland, Nazia Sharfuddin, and Kevin Pottie. 2021. “Access to Refugee and Migrant Mental Health Care Services during the First Six Months of the COVID-19 Pandemic: A Canadian Refugee Clinician Survey.” *International Journal of Environmental Research and Public Health* 18(10):5266. doi: [10.3390/ijerph18105266](https://doi.org/10.3390/ijerph18105266).

Bensley, Kara M. K., Katherine J. Karriker-Jaffe, Cheryl Cherpitel, Libo Li, Lynn S. Wallisch, and Sarah E. Zemore. 2021. “Limited Treatment Accessibility: Implications for Alcohol Treatment Disparities among Mexican Americans Living in the US-Mexico Border Region.” *Journal of Substance Abuse Treatment* 121:108162. doi: [10.1016/j.jsat.2020.108162](https://doi.org/10.1016/j.jsat.2020.108162).

Bettencourt, Amie F., Rebecca A. Ferro, Jami-Lin L. Williams, Kainat N. Khan, Rheanna E. Platt, Sarah Sweeney, and Kelly Coble. 2021. “Pediatric Primary Care Provider Comfort with Mental Health Practices: A Needs Assessment of Regions with Shortages of Treatment Access.” *Academic Psychiatry : The Journal of the American Association of Directors of Psychiatric Residency Training and the Association for Academic Psychiatry* 45(4):429–34. doi: [10.1007/s40596-021-01434-x](https://doi.org/10.1007/s40596-021-01434-x).

Bhavsar, Vishal, Sohail Jannesari, Philip McGuire, James H. MacCabe, Jayati Das-Munshi, Dinesh Bhugra, Sarah Dorrington, June S. L. Brown, Matthew H. Hotopf, and Stephani L. Hatch. 2021. “The Association of Migration and Ethnicity with Use of the Improving Access to Psychological Treatment (IAPT) Programme: A General Population Cohort Study.” *Social Psychiatry and Psychiatric Epidemiology* 56(11):1943–56. doi: [10.1007/s00127-021-02035-7](https://doi.org/10.1007/s00127-021-02035-7).

Bilardi, Jade E., Gemma Sharp, Samantha Payne, and Meredith J. Temple-Smith. 2021. “The Need for Improved Emotional Support: A Pilot Online Survey of Australian Women’s Access to Healthcare Services and Support at the Time of Miscarriage.” *Women and Birth* 34(4):362–69. doi: [10.1016/j.wombi.2020.06.011](https://doi.org/10.1016/j.wombi.2020.06.011).

Bishop, Tara F., Joanna K. Seirup, Harold Alan Pincus, and Joseph S. Ross. 2016. “Population Of US Practicing Psychiatrists Declined, 2003-13, Which May Help Explain Poor Access To Mental Health Care.” *Health Affairs* 35(7):1271–77. doi: [10.1377/hlthaff.2015.1643](https://doi.org/10.1377/hlthaff.2015.1643).

Bjertnaes, Oyvind A., Andrew Garratt, Torleif Ruud, and Steinar Hunskaar. 2010. “The General Practitioner Experiences Questionnaire (GPEQ): Validity and Reliability Following the Inclusion of New Accessibility Items.” *Family Practice* 27(5):513–19. doi: [10.1093/fampra/cmq042](https://doi.org/10.1093/fampra/cmq042).

Black, G., R. M. Roberts, and T. Li-Leng. 2012. “Depression in Rural Adolescents: Relationships with Gender and Availability of Mental Health Services.” *Rural & Remote Health* 12(3):1–11.

Blunt, Elson Oshman, Johanna Catherine Maclean, Ioana Popovici, and Steven C. Marcus. 2020. “Public Insurance Expansions and Mental Health Care Availability.” *Health Services Research* 55(4):615–25. doi: [10.1111/1475-6773.13311](https://doi.org/10.1111/1475-6773.13311).

Bobo, William V., Peter Wollan, Greg Lewis, Susan Bertram, Margary J. Kurland, Kimberle Vore, and Barbara P. Yawn. 2014. “Depressive Symptoms and Access to Mental Health Care in Women Screened for Postpartum Depression Who Lose Health Insurance Coverage After Delivery: Findings From the Translating Research Into Practice for Postpartum Depression (TRIPPD) Effectiveness Study.” *Mayo Clinic Proceedings* 89(9):1220–28. doi: [10.1016/j.mayocp.2014.05.011](https://doi.org/10.1016/j.mayocp.2014.05.011).

Borenstein, D. B. 1985. “Availability of Mental Health Resources for Residents in Academic Medical Centers.” *Journal of Medical Education* 60(7):517–23. doi: [10.1097/00001888-198507000-00002](https://doi.org/10.1097/00001888-198507000-00002).

Boukherroub, Tasseda, Lysane Ouellet, Guillaume Lemay, Nathalie Bibeau, Diane Thiffault, and Nicole McNeil. 2022. “Improving Access to Frontline Psychosocial Services for Youths in Difficulty by Using LSS: An Action Research Case Study.” *International Journal of Lean Six Sigma* 13(4):937–58. doi: [10.1108/IJLSS-08-2020-0134](https://doi.org/10.1108/IJLSS-08-2020-0134).

Bradley, Stacy, and Martin Drapeau. 2014. “Increasing Access to Mental Health Care Through Government-Funded Psychotherapy: The Perspectives of Clinicians.” *Canadian Psychology* 55(2):80–89. doi: [10.1037/a0036453](https://doi.org/10.1037/a0036453).

Breitbart, V., W. Chavkin, and P. H. Wise. 1994. “The Accessibility of Drug Treatment for Pregnant Women: A Survey of Programs in Five Cities.” *American Journal of Public Health* 84(10):1658–61. doi: [10.2105/ajph.84.10.1658](https://doi.org/10.2105/ajph.84.10.1658).

Brice, Samuel, Jacqui Rodgers, Barry Ingham, David Mason, Colin Wilson, Mark Freeston, Ann Le Couteur, and Jeremy R. Parr. 2021. “The Importance and Availability of Adjustments to Improve Access for Autistic Adults Who Need Mental and Physical Healthcare: Findings from UK Surveys.” *BMJ Open* 11(3):e043336. doi: [10.1136/bmjopen-2020-043336](https://doi.org/10.1136/bmjopen-2020-043336).

Britto, Raquel Rodrigues, Marta Supervia, Karam Turk-Adawi, Gabriela Suellen da Silva Chaves, Ella Pesah, Francisco Lopez-Jimenez, Danielle Aparecida Gomes Pereira, Artur H. Herdy, and Sherry L. Grace. 2020. “Cardiac Rehabilitation Availability and Delivery in Brazil: A Comparison to Other Upper Middle-Income Countries.” *Brazilian Journal of Physical Therapy* 24(2):167–76. doi: [10.1016/j.bjpt.2019.02.011](https://doi.org/10.1016/j.bjpt.2019.02.011).

Brooks, Robert G., Nir Menachemi, Art Clawson, and Les Beitsch. 2005. “Availability of Physician Services in Florida, Revisited: The Effect of the Professional Liability Insurance Market on Access to Health Care.” *Archives of Internal Medicine* 165(18):2136–41. doi: [10.1001/archinte.165.18.2136](https://doi.org/10.1001/archinte.165.18.2136).

Brooks, Robert G., Nir Menachemi, Cathy Hughes, and Art Clawson. 2004. “Impact of the Medical Professional Liability Insurance Crisis on Access to Care in Florida.” *Archives of Internal Medicine* 164(20):2217–22. doi: [10.1001/archinte.164.20.2217](https://doi.org/10.1001/archinte.164.20.2217).

Brown, Jonathan D. 2019. “Availability of Integrated Primary Care Services in Community Mental Health Care Settings.” *Psychiatric Services* 70(6):499–502. doi: [10.1176/appi.ps.201800448](https://doi.org/10.1176/appi.ps.201800448).

Campbell, Cynthia I., and Jeffrey A. Alexander. 2006. “Availability of Services for Women in Outpatient Substance Abuse Treatment: 1995-2000.” *The Journal of Behavioral Health Services & Research* 33(1):1–19. doi: [10.1007/s11414-005-9002-2](https://doi.org/10.1007/s11414-005-9002-2).

Cantor, Jonathan H., Ryan K. McBain, Aaron Kofner, Bradley D. Stein, and Hao Yu. 2021. “Availability of Outpatient Telemental Health Services in the United States at the Outset of the COVID-19 Pandemic.” *Medical Care* 59(4):319. doi: [10.1097/MLR.0000000000001512](https://doi.org/10.1097/MLR.0000000000001512).

Cantor, Jonathan, David Powell, Aaron Kofner, and Bradley D. Stein. 2021. “Population-Based Estimates of Geographic Accessibility of Medication for Opioid Use Disorder by Substance Use Disorder Treatment Facilities from 2014 to 2020.” *Drug and Alcohol Dependence* 229(Pt A):109107. doi: [10.1016/j.drugalcdep.2021.109107](https://doi.org/10.1016/j.drugalcdep.2021.109107).

Chang, Wen-Pin, Teresa Chen, Heather Stuart, and Shu-Ping Chen. 2021. “Environmental Scan of Mental Wellness Resources Available on Canadian Post-Secondary Campuses.” *Higher Education* 81(5):1007–21. doi: [10.1007/s10734-020-00594-3](https://doi.org/10.1007/s10734-020-00594-3).

Chen, Christina. 2017. “Cultural Competence of School Psychologists Working with Asian American Youth: Enhancing Accessibility of School Psychology Services.” ProQuest Information & Learning.

Cheng, Tyrone C., and Celia C. Lo. 2012. “Racial Disparities in Access to Needed Child Welfare Services and Worker-Client Engagement.” *Children and Youth Services Review* 34(9):1624–32. doi: [10.1016/j.childyouth.2012.04.021](https://doi.org/10.1016/j.childyouth.2012.04.021).

Chuang, Emmeline, Rebecca Wells, and Jeffrey A. Alexander. 2011. “Public Managed Care and Service Access in Outpatient Substance Abuse Treatment Units.” *The Journal of Behavioral Health Services & Research* 38(4):444–63. doi: [10.1007/s11414-010-9230-y](https://doi.org/10.1007/s11414-010-9230-y).

Clark, Sharon, Debbie Emberly, Kathleen Pajer, Emily Delong, Susan McWilliam, Alexa Bagnell, Sabina Abidi, Barbara Casey, and William Gardner. 2018. “Improving Access to Child and Adolescent Mental Health Care: The Choice and Partnership Approach.” *Journal of the Canadian Academy of Child and Adolescent Psychiatry* 27(1):5–14.

Clemans-Cope, Lisa, Genevieve Kenney, Timothy Waidmann, Michael Huntress, and Nathaniel Anderson. 2015. “How Well Is CHIP Addressing Health Care Access and Affordability for Children?” *Academic Pediatrics* 15(3 Suppl):S71–77. doi: [10.1016/j.acap.2015.02.007](https://doi.org/10.1016/j.acap.2015.02.007).

Cochran, Bryan N., K. Michelle Peavy, and Jennifer S. Robohm. 2007. “Do Specialized Services Exist for LGBT Individuals Seeking Treatment for Substance Misuse? A Study of Available Treatment Programs.” *Substance Use & Misuse* 42(1):161–76. doi: [10.1080/10826080601094207](https://doi.org/10.1080/10826080601094207).

Coker, Tumaini R., Lorena Porras-Javier, Lily Zhang, Neelkamal Soares, Christine Park, Alpa Patel, Lingqi Tang, Paul J. Chung, and Bonnie T. Zima. 2019. “A Telehealth-Enhanced Referral Process in Pediatric Primary Care: A Cluster Randomized Trial.” *Pediatrics* 143(3):e20182738. doi: [10.1542/peds.2018-2738](https://doi.org/10.1542/peds.2018-2738).

Cooper, Sara L., Dennis Lezotte, Jillian Jacobellis, and Carolyn Diguiseppi. 2006. “Does Availability of Mental Health Resources Prevent Recurrent Suicidal Behavior? An Ecological Analysis.” *Suicide & Life-Threatening Behavior* 36(4):409–17. doi: [10.1521/suli.2006.36.4.409](https://doi.org/10.1521/suli.2006.36.4.409).

Creedon, Timothy B., and Benjamin Lê Cook. 2016. “Access To Mental Health Care Increased But Not For Substance Use, While Disparities Remain.” *Health Affairs* 35(6):1017–21. doi: [10.1377/hlthaff.2016.0098](https://doi.org/10.1377/hlthaff.2016.0098).

Cronin, Timothy J., Christopher A. Pepping, W. Kim Halford, and Anthony Lyons. 2021a. “Mental Health Help-Seeking and Barriers to Service Access among Lesbian, Gay, and Bisexual Australians.” *Australian Psychologist* 56(1):46–60. doi: [10.1080/00050067.2021.1890981](https://doi.org/10.1080/00050067.2021.1890981).

Cronin, Timothy J., Christopher A. Pepping, W. Kim Halford, and Anthony Lyons. 2021b. “Minority Stress and Psychological Outcomes in Sexual Minorities: The Role of Barriers to Accessing Services.” *Journal of Homosexuality* 68(14):2417–29. doi: [10.1080/00918369.2020.1804264](https://doi.org/10.1080/00918369.2020.1804264).

Crowley J, Davis DA, and Steadman P. 2006. “Access to and Use of Information Communication Technology.” *Mental Health Practice* 9(6):36–38. doi: [10.7748/mhp2006.03.9.6.36.c1904](https://doi.org/10.7748/mhp2006.03.9.6.36.c1904).

Cummings, Janet R., Lindsay Allen, Julie Clennon, Xu Ji, and Benjamin G. Druss. 2017. “Geographic Access to Specialty Mental Health Care Across High- and Low-Income US Communities.” *JAMA Psychiatry* 74(5):476–84. doi: [10.1001/jamapsychiatry.2017.0303](https://doi.org/10.1001/jamapsychiatry.2017.0303).

Cummings, Janet R., Joseph L. Smith, Sara W. Cullen, and Steven C. Marcus. 2021. “The Changing Landscape of Community Mental Health Care: Availability of Treatment Services in National Data, 2010–2017.” *Psychiatric Services* 72(2):204–8. doi: [10.1176/appi.ps.201900546](https://doi.org/10.1176/appi.ps.201900546).

Cummings, Janet R., Hefei Wen, Michelle Ko, and Benjamin G. Druss. 2014. “Race/Ethnicity and Geographic Access to Medicaid Substance Use Disorder Treatment Facilities in the United States.” *JAMA Psychiatry* 71(2):190–96. doi: [10.1001/jamapsychiatry.2013.3575](https://doi.org/10.1001/jamapsychiatry.2013.3575).

Curry, C., T. Cossich, J. P. Matthews, J. Beresford, and S. A. McLachlan. 2002. “Uptake of Psychosocial Referrals in an Outpatient Cancer Setting: Improving Service Accessibility via the Referral Process.” *Supportive Care in Cancer : Official Journal of the Multinational Association of Supportive Care in Cancer* 10(7):549–55. doi: [10.1007/s00520-002-0371-2](https://doi.org/10.1007/s00520-002-0371-2).

Dalstrom, Matthew, Laurence G. Weinzimmer, Roopa Foulger, and Colleen J. Klein. 2021. “Medicaid Expansion and Accessibility to Healthcare: The Illinois Experience.” *Public Health Nursing* 38(5):720–29. doi: [10.1111/phn.12899](https://doi.org/10.1111/phn.12899).

Davis, Kimberly Diane. 2014. “Availability of Treatment Services for Veterans State by State: A National Problem.” ProQuest Information & Learning.

Davis, Maryann, Jeffrey L. Geller, and Bethany Hunt. 2006. “Within-State Availability of Transition-to-Adulthood Services for Youths with Serious Mental Health Conditions.” *Psychiatric Services* 57(11):1594–99. doi: [10.1176/appi.ps.57.11.1594](https://doi.org/10.1176/appi.ps.57.11.1594).

Dedania, Reema, and Gilbert Gonzales. 2019. “Disparities in Access to Health Care Among US-Born and Foreign-Born US Adults by Mental Health Status, 2013–2016.” *American Journal of Public Health* 109:S221–27. doi: [10.2105/AJPH.2019.305149](https://doi.org/10.2105/AJPH.2019.305149).

DiGiulio, Anne, Zach Jump, Stephen Babb, Anna Schecter, Kisha-Ann S. Williams, Debbie Yembra, and Brian S. Armour. 2020. “State Medicaid Coverage for Tobacco Cessation Treatments and Barriers to Accessing Treatments - United States, 2008-2018.” *MMWR: Morbidity & Mortality Weekly Report* 69(6):155–60. doi: [10.15585/mmwr.mm6906a2](https://doi.org/10.15585/mmwr.mm6906a2).

Dora-Laskey, Aaron, Andrew King, and Richard Sadler. 2022. “Identifying Barriers to Emergency Department-Initiated Buprenorphine: A Spatial Analysis of Treatment Facility Access in Michigan.” *American Journal of Emergency Medicine* 51:393–96. doi: [10.1016/j.ajem.2021.11.014](https://doi.org/10.1016/j.ajem.2021.11.014).

Drake, C., J. M. Donohue, D. Nagy, C. Mair, K. L. Kraemer, and D. J. Wallace. 2020. “Geographic Access to Buprenorphine Prescribers for Patients Who Use Public Transit.” *Journal of Substance Abuse Treatment* 117:108093. doi: [10.1016/j.jsat.2020.108093](https://doi.org/10.1016/j.jsat.2020.108093).

Ducharme LJ, Knudsen HK, and Roman PM. 2006. “Availability of Integrated Care for Co-Occurring Substance Abuse and Psychiatric Conditions.” *Community Mental Health Journal* 42(4):363–75. doi: [10.1007/s10597-005-9030-7](https://doi.org/10.1007/s10597-005-9030-7).

Dunn, S. Hunter, Shari S. Rogal, Marissa M. Maier, Maggie Chartier, Timothy R. Morgan, and Lauren A. Beste. 2019. “Access to Comprehensive Services for Advanced Liver Disease in the Veterans Health Administration.” *Digestive Diseases & Sciences* 64(12):3471–79. doi: [10.1007/s10620-019-05785-2](https://doi.org/10.1007/s10620-019-05785-2).

Durand, Mary Alison, Paul Lelliott, and Nicholas Coyle. 2006. “Availability of Treatment for Substance Misuse in Medium Secure Psychiatric Care in England: A National Survey.” *Journal of Forensic Psychiatry & Psychology* 17(4):611–25. doi: [10.1080/14789940600911577](https://doi.org/10.1080/14789940600911577).

Elton, Eva, and Gilbert Gonzales. 2022. “Health Insurance Coverage and Access to Care by Sexual Orientation and Marital/Cohabitation Status: New Evidence from the 2015-2018 National Health Interview Survey.” *Population Research and Policy Review* 41(2):479–93. doi: [10.1007/s11113-021-09670-7](https://doi.org/10.1007/s11113-021-09670-7).

Erbes, Christopher R., Rebecca Stinson, Eric Kuhn, Melissa Polusny, Jessica Urban, Julia Hoffman, Josef I. Ruzek, Carl Stepnowsky, and Steven R. Thorp. 2014. “Access, Utilization, and Interest in MHealth Applications among Veterans Receiving Outpatient Care for PTSD.” *Military Medicine* 179(11):1218–22. doi: [10.7205/MILMED-D-14-00014](https://doi.org/10.7205/MILMED-D-14-00014).

Fang, Hai, and John A. Rizzo. 2007. “Do Psychiatrists Have Less Access to Medical Services for Their Patients?” *The Journal of Mental Health Policy and Economics* 10(2):63–71.

Fields, Dail, Paul M. Roman, and Terry C. Blum. 2012. “Management Systems, Patient Quality Improvement, Resource Availability, and Substance Abuse Treatment Quality.” *Health Services Research* 47(3 Pt 1):1068–90. doi: [10.1111/j.1475-6773.2011.01352.x](https://doi.org/10.1111/j.1475-6773.2011.01352.x).

Fortney J, Chumbler N, Cody M, and Beck C. 2002. “Geographic Access and Service Use in a Community-Based Sample of Cognitively Impaired Elders.” *Journal of Applied Gerontology* 21(3):352–67. doi: [10.1177/073346480202100305](https://doi.org/10.1177/073346480202100305).

Fortney, John C., Jeff M. Pyne, Matt Hawrilenko, Jared M. Bechtel, Dana Moore, John P. Nolan, Paul Pfeiffer, Stephanie Shushan, Jay H. Shore, and Deb Bowen. 2021. “Psychometric Properties of the Assessment of Perceived Access to Care (APAC) Instrument.” *Journal of Ambulatory Care Management* 44(1):31–45. doi: [10.1097/JAC.0000000000000358](https://doi.org/10.1097/JAC.0000000000000358).

Friedmann, Peter D., Stephenie C. Lemon, Michael D. Stein, and Thomas A. D’Aunno. 2003. “Accessibility of Addiction Treatment: Results from a National Survey of Outpatient Substance Abuse Treatment Organizations.” *Health Services Research* 38(3):887–903. doi: [10.1111/1475-6773.00151](https://doi.org/10.1111/1475-6773.00151).

Gallego, Gisselle, Angela Dew, Michelle Lincoln, Anita Bundy, Rebecca Jean Chedid, Kim Bulkeley, Jennie Brentnall, and Craig Veitch. 2017. “Access to Therapy Services for People with Disability in Rural Australia: A Carers’ Perspective.” *Health & Social Care in the Community* 25(3):1000–1010. doi: [10.1111/hsc.12399](https://doi.org/10.1111/hsc.12399).

Gallo, Kaitlin P., S. Serene Olin, Amy Storfer-Isser, Briannon C. O’Connor, Emma D. Whitmyre, Kimberly E. Hoagwood, and Sarah McCue Horwitz. 2017. “Parent Burden in Accessing Outpatient Psychiatric Services for Adolescent Depression in a Large State System.” *Psychiatric Services* 68(4):411–14. doi: [10.1176/appi.ps.201600111](https://doi.org/10.1176/appi.ps.201600111).

Ghorbanzadeh, Mahyar, Kyusik Kim, Eren Erman Ozguven, and Mark W. Horner. 2020. “A Comparative Analysis of Transportation-Based Accessibility to Mental Health Services.” *Transportation Research Part D-Transport and Environment* 81:102278. doi: [10.1016/j.trd.2020.102278](https://doi.org/10.1016/j.trd.2020.102278).

Ghosheh, Mona Riyad. 2013. “Organizational Cultural Competence and the Availability of Outreach Services for Racial and Ethnic Minorities in University Counseling Centers.” ProQuest Information & Learning.

Gibson, Britton A., Debarchana Ghosh, Jamie P. Morano, and Frederick L. Altice. 2014. “Accessibility and Utilization Patterns of a Mobile Medical Clinic among Vulnerable Populations.” *Health & Place* 28:153–66. doi: [10.1016/j.healthplace.2014.04.008](https://doi.org/10.1016/j.healthplace.2014.04.008).

Goldner EM, Jones W, Fang ML, Elliot M. Goldner, Wayne Jones, and Mei Lan Fang. 2011. “Access to and Waiting Time for Psychiatrist Services in a Canadian Urban Area: A Study in Real Time.” *Canadian Journal of Psychiatry* 56(8):474–80.

Gonzalez, Rebecca Lynn. 2020. “How Perceived Access to Mental Health Care and Perceived Social Stigma Relate to Help-Seeking Attitudes in Urban, Rural, and Frontier Areas.” ProQuest Information & Learning.

Graaf, Genevieve, and Lonnie Snowden. 2020. “Public Health Coverage and Access to Mental Health Care for Youth with Complex Behavioral Healthcare Needs.” *Administration and Policy in Mental Health* 47(3):395–409. doi: [10.1007/s10488-019-00995-2](https://doi.org/10.1007/s10488-019-00995-2).

Graves, Janessa M., Demetrius A. Abshire, Jessica L. Mackelprang, Solmaz Amiri, and Ashley Beck. 2020. “Association of Rurality With Availability of Youth Mental Health Facilities With Suicide Prevention Services in the US.” *JAMA Network Open* 3(10):e2021471–e2021471. doi: [10.1001/jamanetworkopen.2020.21471](https://doi.org/10.1001/jamanetworkopen.2020.21471).

Green, Schelle Mcmakin. 1996. “A Comparative Study of Mental Health Services Available to Students in South Carolina’s Ninety-One School Districts.” ProQuest Information & Learning.

Grembowski, David E., Diane Martin, Donald L. Patrick, Paula Diehr, Wayne Katon, Barbara Williams, Ruth Engelberg, Louise Novak, Deborah Dickstein, Richard Deyo, and Harold I. Goldberg. 2002. “Managed Care, Access to Mental Health Specialists, and Outcomes among Primary Care Patients with Depressive Symptoms.” *Journal of General Internal Medicine* 17(4):258–69. doi: [10.1046/j.1525-1497.2002.10321.x](https://doi.org/10.1046/j.1525-1497.2002.10321.x).

Guerrero, Erick G., Karen B. Pan, Andrew Curtis, and Erica L. Lizano. 2011. “Availability of Substance Abuse Treatment Services in Spanish: A GIS Analysis of Latino Communities in Los Angeles County, California.” *Substance Abuse Treatment, Prevention, and Policy* 6:21. doi: [10.1186/1747-597X-6-21](https://doi.org/10.1186/1747-597X-6-21).

Gulliver, Suzy B., Michelle L. Pennington, Victoria A. Torres, Laurie E. Steffen, Amruta Mardikar, Frank Leto, William Ostiguy, Rose T. Zimering, and Nathan A. Kimbrel. 2019. “Behavioral Health Programs in Fire Service: Surveying Access and Preferences.” *Psychological Services* 16(2):340–45. doi: [10.1037/ser0000222](https://doi.org/10.1037/ser0000222).

Haley, Sean J., Susan Moscou, Sharifa Murray, Traci Rieckmann, and Kameron Wells. 2018. “The Availability of Alcohol, Tobacco, and Other Drug Services for Adolescents in New York State Community Health Centers.” *Journal of Drug Issues* 48(1):78–89. doi: [10.1177/0022042617731132](https://doi.org/10.1177/0022042617731132).

Harris, Samantha J., Amanda J. Abraham, Christina M. Andrews, and Courtney R. Yarbrough. 2020. “Gaps In Access To Opioid Use Disorder Treatment For Medicare Beneficiaries.” *Health Affairs* 39(2):233–37. doi: [10.1377/hlthaff.2019.00309](https://doi.org/10.1377/hlthaff.2019.00309).

Hartley D, Ziller EC, Loux SL, Gale JA, Lambert D, and Yousefian AE. 2007. “Use of Critical Access Hospital Emergency Rooms by Patients with Mental Health Symptoms.” *Journal of Rural Health* 23(2):108–15. doi: [10.1111/j.1748-0361.2007.00077.x](https://doi.org/10.1111/j.1748-0361.2007.00077.x).

Heneghan AM and Malakoff ME. 1997. “Availability of School Health Services for Young Children.” *Journal of School Health* 67(8):327–32. doi: [10.1111/j.1746-1561.1997.tb03466.x](https://doi.org/10.1111/j.1746-1561.1997.tb03466.x).

Henning-Smith, Carrie, and Sirry Alang. 2016. “Access to Care for Children with Emotional/Behavioral Difficulties.” *Journal of Child Health Care : For Professionals Working with Children in the Hospital and Community* 20(2):185–94. doi: [10.1177/1367493514563855](https://doi.org/10.1177/1367493514563855).

Hilton, N. Zoe, and Chelsea Turan. 2014. “Availability of Services for Parents Living with Mental Disorders: A Province-Wide Survey.” *Psychiatric Rehabilitation Journal* 37(3):194–200. doi: [10.1037/prj0000055](https://doi.org/10.1037/prj0000055).

Hirchak, Katherine A., and Sean M. Murphy. 2017. “Assessing Differences in the Availability of Opioid Addiction Therapy Options: Rural Versus Urban and American Indian Reservation Versus Nonreservation.” *Journal of Rural Health* 33(1):102–9. doi: [10.1111/jrh.12178](https://doi.org/10.1111/jrh.12178).

Hodgkin, Dominic, Constance M. Horgan, Maureen T. Stewart, Amity E. Quinn, Timothy B. Creedon, Sharon Reif, and Deborah W. Garnick. 2018. “Federal Parity and Access to Behavioral Health Care in Private Health Plans.” *Psychiatric Services* 69(4):396–402. doi: [10.1176/appi.ps.201700203](https://doi.org/10.1176/appi.ps.201700203).

Hohman, Jessica A., Kathryn A. Martinez, Amit Anand, Mark Rood, Trejeeve Martyn, Susannah Rose, and Michael B. Rothberg. 2022. “Use of Direct-to-Consumer Telemedicine to Access Mental Health Services.” *Journal of General Internal Medicine* 37(11):2759–67. doi: [10.1007/s11606-021-07326-y](https://doi.org/10.1007/s11606-021-07326-y).

Holstein, Russell M., and David P. Paul III. 2017. “Access to Behavioral Health Care Services in New Jersey.” *Hospital Topics* 95(3):51–56. doi: [10.1080/00185868.2017.1300481](https://doi.org/10.1080/00185868.2017.1300481).

Hu, Allison D. S. 2010. “Substance Abuse and Mental Health Treatment Accessibility and Availability in Moloka’i.” ProQuest Information & Learning.

Huhn, Andrew S., J. Gregory Hobelmann, Aaron Ramirez, Eric C. Strain, and George A. Oyler. 2019. “Trends in First-Time Treatment Admissions for Older Adults with Alcohol Use Disorder: Availability of Medical and Specialty Clinical Services in Hospital, Residential, and Outpatient Facilities.” *Drug and Alcohol Dependence* 205:107694. doi: [10.1016/j.drugalcdep.2019.107694](https://doi.org/10.1016/j.drugalcdep.2019.107694).

Huhn, Andrew S., J. Gregory Hobelmann, Justin C. Strickland, George A. Oyler, Cecilia L. Bergeria, Annie Umbricht, and Kelly E. Dunn. 2020. “Differences in Availability and Use of Medications for Opioid Use Disorder in Residential Treatment Settings in the United States.” *JAMA Network Open* 3(2):e1920843. doi: [10.1001/jamanetworkopen.2019.20843](https://doi.org/10.1001/jamanetworkopen.2019.20843).

Hung, Peiyin, Susan H. Busch, Yi-Wen Shih, Alecia J. McGregor, and Shiyi Wang. 2020. “Changes in Community Mental Health Services Availability and Suicide Mortality in the US: A Retrospective Study.” *BMC Psychiatry* 20(1):188. doi: [10.1186/s12888-020-02607-y](https://doi.org/10.1186/s12888-020-02607-y).

Huskamp, Haiden A., Joyce C. West, Donald S. Rae, Maritza Rubio-Stipec, Darrel A. Regier, and Richard G. Frank. 2009. “Part D and Dually Eligible Patients with Mental Illness: Medication Access Problems and Use of Intensive Services.” *Psychiatric Services* 60(9):1169–74. doi: [10.1176/appi.ps.60.9.1169](https://doi.org/10.1176/appi.ps.60.9.1169).

Johns, Louise, Suzanne Jolley, Philippa Garety, Mizanur Khondoker, Miriam Fornells-Ambrojo, Juliana Onwumere, Emmanuelle Peters, Craig Milosh, Alison Brabban, and Majella Byrne. 2019. “Improving Access to Psychological Therapies for People with Severe Mental Illness (IAPT-SMI): Lessons from the South London and Maudsley Psychosis Demonstration Site.” *Behaviour Research and Therapy* 116:104–10. doi: [10.1016/j.brat.2019.03.002](https://doi.org/10.1016/j.brat.2019.03.002).

Johnson, Andrew Osborne. 2009. “The Geographic Availability of Substance Abuse Treatment Facilities and Services to Rural Veterans of the United States Armed Forces.” ProQuest Information & Learning.

Johnson, Karen C., Lisa M. Klesges, Grant W. Somes, Mace C. Coday, and Margaret DeBon. 2004. “Access of Over-the-Counter Nicotine Replacement Therapy Products to Minors.” *Archives of Pediatrics & Adolescent Medicine* 158(3):212–16. doi: [10.1001/archpedi.158.3.212](https://doi.org/10.1001/archpedi.158.3.212).

Johnson, P. J., E. B. Tucker, B. A. Bradbury, and F. J. Spencer. 1969. “Survey of Suicide Counseling Available to Students in Metropolitan Richmond.” *Public Health Reports (Washington, D.C. : 1896)* 84(2):118–20.

Johnson, Pamela Jo, Judy Jou, and Dawn M. Upchurch. 2020. “Psychological Distress and Access to Care Among Midlife Women.” *Journal of Aging and Health* 32(5–6):317–27. doi: [10.1177/0898264318822367](https://doi.org/10.1177/0898264318822367).

Johnston, Natalie E. 2015. “Delays in Accessing Electroconvulsive Therapy: A Comparison between Two Urban and Two Rural Populations in Australia.” *Australasian Psychiatry* 23(5):566–70. doi: [10.1177/1039856215597540](https://doi.org/10.1177/1039856215597540).

Jones, Christopher W., Zachary Christman, Christopher M. Smith, Michelle R. Safferman, Matthew Salzman, Kaitlan Baston, and Rachel Haroz. 2018. “Comparison between Buprenorphine Provider Availability and Opioid Deaths among US Counties.” *Journal of Substance Abuse Treatment* 93:19–25. doi: [10.1016/j.jsat.2018.07.008](https://doi.org/10.1016/j.jsat.2018.07.008).

Jones, Emily, Lydie A. Lebrun-Harris, Alek Sripipatana, and Quyen Ngo-Metzger. 2014. “Access to Mental Health Services Among Patients at Health Centers and Factors Associated with Unmet Needs.” *Journal of Health Care for the Poor & Underserved* 25(1):425–36.

Kalb, Luther G., Calliope Holingue, Emma K. Stapp, Kathryn Van Eck, and Johannes Thrul. 2022. “Trends and Geographic Availability of Emergency Psychiatric Walk-In and Crisis Services in the United States.” *Psychiatric Services* 73(1):26–31. doi: [10.1176/appi.ps.202000612](https://doi.org/10.1176/appi.ps.202000612).

Kao, Dennis, Luis R. Torres, Erick G. Guerrero, Rebecca L. Mauldin, and Patrick S. Bordnick. 2014. “Spatial Accessibility of Drug Treatment Facilities and the Effects on Locus of Control, Drug Use, and Service Use among Heroin-Injecting Mexican American Men.” *The International Journal on Drug Policy* 25(3):598–607. doi: [10.1016/j.drugpo.2013.12.012](https://doi.org/10.1016/j.drugpo.2013.12.012).

Kaplan DW, Brindis CD, Phibbs SL, Melinkovich P, Naylor K, and Ahlstrand K. 1999. “A Comparison Study of an Elementary School-Based Health Center: Effects on Health Care Access and Use.” *Archives of Pediatrics & Adolescent Medicine* 153(3):235–43. doi: [10.1001/archpedi.153.3.235](https://doi.org/10.1001/archpedi.153.3.235).

Kaufman, K. L., C. Harbeck, R. Olson, and R. Nitschke. 1992. “The Availability of Psychosocial Interventions to Children with Cancer and Their Families.” *Children’s Health Care : Journal of the Association for the Care of Children’s Health* 21(1):21–25. doi: [10.1207/s15326888chc2101_3](https://doi.org/10.1207/s15326888chc2101_3).

Kempe A, Beaty BL, Crane LA, Stokstad J, Barrow J, Belman S, and Steiner JF. 2005. “Changes in Access, Utilization, and Quality of Care after Enrollment into a State Child Health Insurance Plan.” *Pediatrics* 115(2):364–71. doi: [10.1542/peds.2004-0475](https://doi.org/10.1542/peds.2004-0475).

Kenicer, David, Carrie-Anne McClay, and Christopher Williams. 2012. “A National Survey of Health Service Infrastructure and Policy Impacts on Access to Computerised CBT in Scotland.” *BMC Medical Informatics and Decision Making* 12:102. doi: [10.1186/1472-6947-12-102](https://doi.org/10.1186/1472-6947-12-102).

Kertesz, Stefan G., Whitney McNeil, Julie J. Cash, Renee Desmond, Gerald McGwin Jr, Jason Kelly, and Travis P. Baggett. 2014. “Unmet Need for Medical Care and Safety Net Accessibility among Birmingham’s Homeless.” *Journal of Urban Health : Bulletin of the New York Academy of Medicine* 91(1):33–45. doi: [10.1007/s11524-013-9801-3](https://doi.org/10.1007/s11524-013-9801-3).

Kim, Shannon H., and Philip G. Cooker. 2001. “Accessibility and Appropriateness of Community-Based Mental Health Services to Persons with Developmental Disabilities.” *Mental Health Aspects of Developmental Disabilities* 4(3):108–18.

Kimerling R and Baumrind N. 2005. “Access to Specialty Mental Health Services among Women in California.” *Psychiatric Services* 56(6):729–34. doi: [10.1176/appi.ps.56.6.729](https://doi.org/10.1176/appi.ps.56.6.729).

Kiselev, Nikolai, Naser Morina, Matthis Schick, Birgit Watzke, Ulrich Schnyder, and Monique C. Pfaltz. 2020. “Barriers to Access to Outpatient Mental Health Care for Refugees and Asylum Seekers in Switzerland: The Therapist’s View.” *BMC Psychiatry* 20(1):378. doi: [10.1186/s12888-020-02783-x](https://doi.org/10.1186/s12888-020-02783-x).

Knight, Bob G., and Sonya Winterbotham. 2020. “Rural and Urban Older Adults’ Perceptions of Mental Health Services Accessibility.” *Aging & Mental Health* 24(6):978–84. doi: [10.1080/13607863.2019.1576159](https://doi.org/10.1080/13607863.2019.1576159).

Knudsen, Hannah K. 2009. “Adolescent-Only Substance Abuse Treatment: Availability and Adoption of Components of Quality.” *Journal of Substance Abuse Treatment* 36(2):195–204. doi: [10.1016/j.jsat.2008.06.002](https://doi.org/10.1016/j.jsat.2008.06.002).

Knudsen, Hannah K., Lori J. Ducharme, and Paul M. Roman. 2007. “Racial and Ethnic Disparities in SSRI Availability in Substance Abuse Treatment.” *Psychiatric Services* 58(1):55–62. doi: [10.1176/appi.ps.58.1.55](https://doi.org/10.1176/appi.ps.58.1.55).

Knudsen, Hannah K., and Paul M. Roman. 2015. “Medicaid, Private Insurance, and the Availability of Smoking Cessation Interventions in Substance Use Disorder Treatment.” *Psychiatric Services* 66(11):1213–20. doi: [10.1176/appi.ps.201400451](https://doi.org/10.1176/appi.ps.201400451).

Knudsen, Hannah K., Paul M. Roman, and Lori J. Ducharme. 2004. “The Availability of Psychiatric Programs in Private Substance Abuse Treatment Centers, 1995 to 2001.” *Psychiatric Services* 55(3):270–73. doi: [10.1176/appi.ps.55.3.270](https://doi.org/10.1176/appi.ps.55.3.270).

Knudsen, Hannah K., and Jamie L. Studts. 2011. “Availability of Nicotine Replacement Therapy in Substance Use Disorder Treatment: Longitudinal Patterns of Adoption, Sustainability, and Discontinuation.” *Drug and Alcohol Dependence* 118(2–3):244–50. doi: [10.1016/j.drugalcdep.2011.03.028](https://doi.org/10.1016/j.drugalcdep.2011.03.028).

Koch AL, Arfken CL, and Schuster CR. 2006. “Characteristic of U.S. Substance Abuse Treatment of Facilities Adopting Buprenorphine in Its Initial Stage of Availability.” *Drug & Alcohol Dependence* 83(3):274–78. doi: [10.1016/j.drugalcdep.2005.12.005](https://doi.org/10.1016/j.drugalcdep.2005.12.005).

Kong, Yinfei, Jia Zhou, Zemin Zheng, Hortensia Amaro, and Erick G. Guerrero. 2022. “Using Machine Learning to Advance Disparities Research: Subgroup Analyses of Access to Opioid Treatment.” *Health Services Research* 57(2):411–21. doi: [10.1111/1475-6773.13896](https://doi.org/10.1111/1475-6773.13896).

König, Daniel, Matthäus Fellinger, Nathalie Pruckner, Barbara Hinterbuchinger, Georg Dorffner, Andreas Gleiss, Sandra Vyssoki, and Benjamin Vyssoki. 2018. “Availability and Use of Mental Health Services in European Countries: Influence on National Suicide Rates.” *Journal of Affective Disorders* 239:66–71. doi: [10.1016/j.jad.2018.06.042](https://doi.org/10.1016/j.jad.2018.06.042).

Krawczyk, Noa, Brady Garrett, N. Jia Ahmad, Esita Patel, Keisha Solomon, Elizabeth A. Stuart, and Brendan Saloner. 2021. “Medications for Opioid Use Disorder among American Indians and Alaska Natives: Availability and Use across a National Sample.” *Drug and Alcohol Dependence* 220:108512. doi: [10.1016/j.drugalcdep.2021.108512](https://doi.org/10.1016/j.drugalcdep.2021.108512).

Kreutzberg, Anika, and Rowena Jacobs. 2020. “Improving Access to Services for Psychotic Patients: Does Implementing a Waiting Time Target Make a Difference.” *European Journal of Health Economics* 21(5):703–16. doi: [10.1007/s10198-020-01165-0](https://doi.org/10.1007/s10198-020-01165-0).

Kruk ME, Rockers PC, Williams EH, Varpilah ST, Macauley R, Saydee G, and Galea S. 2010. “Availability of Essential Health Services in Post-Conflict Liberia.” *Bulletin of the World Health Organization* 88(7):527–34. doi: [10.2471/BLT.09.071068](https://doi.org/10.2471/BLT.09.071068).

Kugelmass, Heather. 2016. “‘Sorry, I’m Not Accepting New Patients’: An Audit Study of Access to Mental Health Care.” *Journal of Health and Social Behavior* 57(2):168–83. doi: [10.1177/0022146516647098](https://doi.org/10.1177/0022146516647098).

Kugelmass, Heather. 2018. “Racial Disparities in Access to Mental Health Care: A Field Experimental Approach.” ProQuest Information & Learning.

Kvig, Erling Inge, Beate Brinchmann, Cathrine Moe, Steinar Nilssen, Tor Ketil Larsen, and Knut Sorgaard. 2017. “Geographical Accessibility and Duration of Untreated Psychosis: Distance as a Determinant of Treatment Delay.” *Bmc Psychiatry* 17:176. doi: [10.1186/s12888-017-1345-8](https://doi.org/10.1186/s12888-017-1345-8).

Kyanko, Kelly A., Leslie A Curry, Danya E Keene, Ryan Sutherland, Krishna Naik, and Susan H. Busch. 2022. “Does Primary Care Fill the Gap in Access to Specialty Mental Health Care? A Mixed Methods Study.” *Journal of General Internal Medicine* 37(7):1641–47. doi: [10.1007/s11606-021-07260-z](https://doi.org/10.1007/s11606-021-07260-z).

Lee, Hyunjung, and Gopal K. Singh. 2021. “Monthly Trends in Access to Care and Mental Health Services by Household Income Level During the COVID-19 Pandemic, United States, April: December 2020.” *Health Equity* 5(1):770–79. doi: [10.1089/heq.2021.0036](https://doi.org/10.1089/heq.2021.0036).

Lewis, Valerie A., Steven Spivack, Genevra F. Murray, and Hector P. Rodriguez. 2021. “FQHC Designation and Safety Net Patient Revenue Associated with Primary Care Practice Capabilities for Access and Quality.” *JGIM: Journal of General Internal Medicine* 36(10):2922–28. doi: [10.1007/s11606-021-06746-0](https://doi.org/10.1007/s11606-021-06746-0).

Lora, A., F. Hanna, and D. Chisholm. 2017. “Mental Health Service Availability and Delivery at the Global Level: An Analysis by Countries’ Income Level from WHO’s Mental Health Atlas 2014.” *Epidemiology and Psychiatric Sciences* 1–12. doi: [10.1017/S2045796017000075](https://doi.org/10.1017/S2045796017000075).

Lora, Antonio, Robert Kohn, Itzhak Levav, Ryan McBain, Jodi Morris, and Shekhar Saxena. 2012. “Service Availability and Utilization and Treatment Gap for Schizophrenic Disorders: A Survey in 50 Low- and Middle-Income Countries.” *Bulletin of the World Health Organization* 90(1):47. doi: [10.2471/BLT.11.089284](https://doi.org/10.2471/BLT.11.089284).

Lottman, Thomas J. 1993. “Access to Generic Substance Abuse Services for Persons with Mental Retardation.” *Journal of Alcohol and Drug Education* 39(1):41–55.

Maguire-Jack, Kathryn, Yiwen Cao, and Susan Yoon. 2018. “Racial Disparities in Child Maltreatment: The Role of Social Service Availability.” *Children & Youth Services Review* 86:49–55. doi: [10.1016/j.childyouth.2018.01.014](https://doi.org/10.1016/j.childyouth.2018.01.014).

Manuel, Jennifer I. 2017. “The Grand Challenge of Reducing Gender and Racial/Ethnic Disparities in Service Access and Needs Among Adults with Alcohol Misuse.” *Journal of Social Work Practice in the Addictions* 17(1/2):10–35. doi: [10.1080/1533256X.2017.1302887](https://doi.org/10.1080/1533256X.2017.1302887).

Masland, Mary C., Lonnie R. Snowden, and Neal T. Wallace. 2007. “Assessment, Authorization and Access to Medicaid Managed Mental Health Care.” *Administration and Policy in Mental Health* 34(6):548–62. doi: [10.1007/s10488-007-0138-7](https://doi.org/10.1007/s10488-007-0138-7).

McBain, Ryan, Daniel J. Norton, Jodi Morris, M. Taghi Yasamy, and Theresa S. Betancourt. 2012. “The Role of Health Systems Factors in Facilitating Access to Psychotropic Medicines: A Cross-Sectional Analysis of the WHO-AIMS in 63 Low- and Middle-Income Countries.” *PLoS Medicine* 9(1):e1001166. doi: [10.1371/journal.pmed.1001166](https://doi.org/10.1371/journal.pmed.1001166).

McCarthy, John F., Frederic C. Blow, Marcia Valenstein, Ellen P. Fischer, Richard R. Owen, Kristen L. Barry, Teresa J. Hudson, and Rosalinda V. Ignacio. 2007. “Veterans Affairs Health System and Mental Health Treatment Retention among Patients with Serious Mental Illness: Evaluating Accessibility and Availability Barriers.” *Health Services Research* 42(3 Pt 1):1042–60. doi: [10.1111/j.1475-6773.2006.00642.x](https://doi.org/10.1111/j.1475-6773.2006.00642.x).

McCarthy, John F., Marcia Valenstein, Kara Zivin, John E. Zeber, and Amy M. Kilbourne. 2010. “Access-Related Measures and out-of-System Utilization among Veterans with Bipolar Disorder.” *Psychiatric Services* 61(10):1035–38. doi: [10.1176/ps.2010.61.10.1035](https://doi.org/10.1176/ps.2010.61.10.1035).

McClelland, Dawn Elizabeth. 2000. “Psychological and Emotional Support Services Available to Burn Survivors, Their Families and Medical and Professional Staff at Major Burn Centers in the United States and Canada.” ProQuest Information & Learning.

McDonnall, Michele C., Adele Crudden, B. J. LeJeune, and Anne Carter Steverson. 2017. “Availability of Mental Health Services for Individuals Who Are Deaf or Deaf-Blind.” *Journal of Social Work in Disability & Rehabilitation* 16(1):1–13. doi: [10.1080/1536710X.2017.1260515](https://doi.org/10.1080/1536710X.2017.1260515).

McEntee, M. K. 1993. “Accessibility of Mental Health Services and Crisis Intervention to the Deaf.” *American Annals of the Deaf* 138(1):26–30. doi: [10.1353/aad.2012.0569](https://doi.org/10.1353/aad.2012.0569).

McInnerney, Daisy, Bridget Candy, Patrick Stone, Nicola Atkin, Joana Johnson, Syd Hiskey, and Nuriye Kupeli. 2021. “Access to and Adequacy of Psychological Services for Adult Patients in UK Hospices: A National, Cross-Sectional Survey.” *BMC Palliative Care* 20(1):1–10. doi: [10.1186/s12904-021-00724-3](https://doi.org/10.1186/s12904-021-00724-3).

McLean, Karen, Harriet Hiscock, Dorothy Scott, and Sharon Goldfeld. 2021. “Foster and Kinship Carer Survey: Accessing Health Services for Children in out-of-Home Care.” *Journal of Paediatrics and Child Health* 57(1):132–39. doi: [10.1111/jpc.15157](https://doi.org/10.1111/jpc.15157).

Mericle, Amy A., Amelia M. Arria, Kathy Meyers, John Cacciola, Ken C. Winters, and Kim Kirby. 2015. “National Trends in Adolescent Substance Use Disorders and Treatment Availability: 2003–2010.” *Journal of Child & Adolescent Substance Abuse* 24(5):255–63. doi: [10.1080/1067828X.2013.829008](https://doi.org/10.1080/1067828X.2013.829008).

Merrick, Elizabeth L., Constance M. Horgan, Deborah W. Garnick, Sharon Reif, and Maureen T. Stewart. 2009. “Accessing Specialty Behavioral Health Treatment in Private Health Plans.” *The Journal of Behavioral Health Services & Research* 36(4):420–35. doi: [10.1007/s11414-008-9161-z](https://doi.org/10.1007/s11414-008-9161-z).

Metraux, Stephen, Eugene Brusilovskiy, Janet A. Prvu-Bettger, Yin-Ling Irene Wong, and Mark S. Salzer. 2012. “Geographic Access to and Availability of Community Resources for Persons Diagnosed with Severe Mental Illness in Philadelphia, USA.” *Health & Place* 18(3):621–29. doi: [10.1016/j.healthplace.2011.12.011](https://doi.org/10.1016/j.healthplace.2011.12.011).

Michaud, Pierre-André, Annemieke Visser, Johanna P. M. Vervoort, Paul Kocken, Sijmen A. Reijneveld, and Danielle E. M. C. Jansen. 2020. “Availability and Accessibility of Primary Mental Health Services for Adolescents: An Overview of National Recommendations and Services in EU.” *European Journal of Public Health* 30(6):1127–33. doi: [10.1093/eurpub/ckaa102](https://doi.org/10.1093/eurpub/ckaa102).

Miller, Kipyn. 2014. “Care Coordination Impacts on Access to Care for Children with Special Health Care Needs Enrolled in Medicaid and CHIP.” *Maternal & Child Health Journal* 18(4):864–72. doi: [10.1007/s10995-013-1312-z](https://doi.org/10.1007/s10995-013-1312-z).

Moore, Jennifer E., Atlang Mompe, and Ernest Moy. 2018. “Disparities by Sex Tracked in the 2015 National Healthcare Quality and Disparities Report: Trends across National Quality Strategy Priorities, Health Conditions, and Access Measures.” *Women’s Health Issues* 28(1):97–103. doi: [10.1016/j.whi.2017.08.006](https://doi.org/10.1016/j.whi.2017.08.006).

Moore, Megan, Nathalia Jimenez, Ali Rowhani-Rahbar, Margaret Willis, Kate Baron, Jessica Giordano, Deborah Crawley, Frederick P. Rivara, Kenneth M. Jaffe, and Beth E. Ebel. 2016. “Availability of Outpatient Rehabilitation Services for Children After Traumatic Brain Injury.” *American Journal of Physical Medicine & Rehabilitation* 95(3):204–13. doi: [10.1097/PHM.0000000000000362](https://doi.org/10.1097/PHM.0000000000000362).

Morita T and Yamazaki H. 2006. “Multidimensional Structures of Comprehensive Accessibility of Community Support for People with Mental Disorders in Japan: A Nationwide Investigation.” *International Journal of Nursing Practice (Wiley-Blackwell)* 12(1):14–20. doi: [10.1111/j.1440-172x.2006.00544.x](https://doi.org/10.1111/j.1440-172x.2006.00544.x).

Morley B, Pirkis J, Naccarella L, Kohn F, Blashki G, and Burgess P. 2007. “Improving Access to and Outcomes from Mental Health Care in Rural Australia.” *Australian Journal of Rural Health* 15(5):304–12. doi: [10.1111/j.1440-1584.2007.00905.x](https://doi.org/10.1111/j.1440-1584.2007.00905.x).

Morrissey J, Calloway M, Johnsen M, and Ullman M. 1997. “Service System Performance and Integration: A Baseline Profile of the ACCESS Demonstration Sites.” *Psychiatric Services* 48(3):374–80.

Morrissey, Karyn, Graham Clarke, Paul Williamson, Antoinette Daly, and Cathal O’Donoghue. 2015. “Mental Illness in Ireland: Simulating Its Geographical Prevalence and the Role of Access to Services.” *Environment and Planning B-Planning & Design* 42(2):338–53. doi: [10.1068/b130054p](https://doi.org/10.1068/b130054p).

Morton, Cory M. 2013. “The Moderating Effect of Substance Abuse Service Accessibility on the Relationship between Child Maltreatment and Neighborhood Alcohol Availability.” *Children and Youth Services Review* 35(12):1933–40. doi: [10.1016/j.childyouth.2013.09.019](https://doi.org/10.1016/j.childyouth.2013.09.019).

Mościcki, Eve K., Joyce C. West, Donald S. Rae, Maritza Rubio-Stipec, Joshua E. Wilk, and Darrel A. Regier. 2010. “Suicidality Is Associated with Medication Access Problems in Publicly Insured Psychiatric Patients.” *The Journal of Clinical Psychiatry* 71(12):1657–63. doi: [10.4088/JCP.10m06177gre](https://doi.org/10.4088/JCP.10m06177gre).

Muilenburg, Jessica L., Tanja C. Laschober, and Lillian T. Eby. 2015. “Substance Use Disorder Counselors’ Reports of Tobacco Cessation Services Availability, Implementation, and Tobacco-Related Knowledge.” *Journal of Adolescent Health* 57(3):327–33. doi: [10.1016/j.jadohealth.2015.06.001](https://doi.org/10.1016/j.jadohealth.2015.06.001).

Mulder, Cornelis L., Torleif Ruud, Michiel Bahler, Hans Kroon, and Stefan Priebe. 2014. “The Availability and Quality across Europe of Outpatient Care for Difficult-to-Engage Patients with Severe Mental Illness: A Survey among Experts.” *International Journal of Social Psychiatry* 60(3):304–10. doi: [10.1177/0020764013485941](https://doi.org/10.1177/0020764013485941).

Munro-Ludders, Bruce, Thomas Simpatico, and Daria Zvetina. 2004. “Making Public Mental-Health Services Accessible to Deaf Consumers: Illinois Deaf Services 2000.” *American Annals of the Deaf* 148(5):396–402. doi: [10.1353/aad.2004.0008](https://doi.org/10.1353/aad.2004.0008).

Murray, Greg, Fiona Judd, Henry Jackson, Caitlin Fraser, Angela Komiti, Gene Hodgins, Pip Pattison, John Humphreys, and Garry Robins. 2004. “Rurality and Mental Health: The Role of Accessibility.” *The Australian and New Zealand Journal of Psychiatry* 38(8):629–34. doi: [10.1080/j.1440-1614.2004.01426.x](https://doi.org/10.1080/j.1440-1614.2004.01426.x).

Myers B. 2010. “Limited Access to HIV Risk-Reduction Services in South African Substance Abuse Treatment Facilities.” *Drugs: Education, Prevention & Policy* 17(6):749–61. doi: [10.3109/09687630903300411](https://doi.org/10.3109/09687630903300411).

Myers, Bronwyn, and Charles D. H. Parry. 2005. “Access to Substance Abuse Treatment Services for Black South Africans: Findings from Audits of Specialist Treatment Facilities in Cape Town and Gauteng.” *South African Psychiatry Review* 8(1):15–19.

Nemoto, Tooru, Taylor M. Cruz, Mariko Iwamoto, and Maria Sakata. 2015. “A Tale of Two Cities: Access to Care and Services Among African-American Transgender Women in Oakland and San Francisco.” *LGBT Health* 2(3):235–42. doi: [10.1089/lgbt.2014.0046](https://doi.org/10.1089/lgbt.2014.0046).

Newman, Mark W., Matt Hawrilenko, Matthew Jakupcak, Shiyu Chen, and John C. Fortney. 2022. “Access and Attitudinal Barriers to Engagement in Integrated Primary Care Mental Health Treatment for Rural Populations.” *The Journal of Rural Health : Official Journal of the American Rural Health Association and the National Rural Health Care Association* 38(4):721–27. doi: [10.1111/jrh.12616](https://doi.org/10.1111/jrh.12616).

Ngamini Ngui, André, and Alain Vanasse. 2012. “Assessing Spatial Accessibility to Mental Health Facilities in an Urban Environment.” *Spatial and Spatio-Temporal Epidemiology* 3(3):195–203. doi: [10.1016/j.sste.2011.11.001](https://doi.org/10.1016/j.sste.2011.11.001).

Nickels, Samuel V., Mariely Campos Tomasino, Nelson A. Flamenco Arvaiza, and Cynthia A. Hunter. 2018. “Access to Mental Health Care in El Salvador: A Case Study of Progress toward Decentralization.” *Revista Panamericana de Salud Pública* 42:1–9. doi: [10.26633/RPSP.2018.172](https://doi.org/10.26633/RPSP.2018.172).

Novak, Priscilla, Andrew C. Anderson, and Jie Chen. 2018. “Changes in Health Insurance Coverage and Barriers to Health Care Access Among Individuals with Serious Psychological Distress Following the Affordable Care Act.” *Administration and Policy in Mental Health* 45(6):924–32. doi: [10.1007/s10488-018-0875-9](https://doi.org/10.1007/s10488-018-0875-9).

Oates, Lloyd Louis, and Nick Firth. 2020. “Deprivation, Access and Outcomes in Health Psychology Treatment.” *Mental Health Review Journal* 25(2):139–51. doi: [10.1108/MHRJ-02-2020-0010](https://doi.org/10.1108/MHRJ-02-2020-0010).

Ogawa, Asao, Junko Nouno, Yuki Shirai, Osamu Shibayama, Kyoko Kondo, Minori Yokoo, Hiroyuki Takei, Harumi Koga, Daisuke Fujisawa, Ken Shimizu, and Yosuke Uchitomi. 2012. “Availability of Psychiatric Consultation-Liaison Services as an Integral Component of Palliative Care Programs at Japanese Cancer Hospitals.” *Japanese Journal of Clinical Oncology* 42(1):42–52. doi: [10.1093/jjco/hyr174](https://doi.org/10.1093/jjco/hyr174).

Ogliari, Giulia, Zoë Turner, Javid Khalique, Adam L. Gordon, John R. F. Gladman, and Neil H. Chadborn. 2020. “Ethnic Disparity in Access to the Memory Assessment Service between South Asian and White British Older Adults in the United Kingdom: A Cohort Study.” *International Journal of Geriatric Psychiatry* 35(5):507–15. doi: [10.1002/gps.5263](https://doi.org/10.1002/gps.5263).

Ohl, Michael E., Margaret Carrell, Andrew Thurman, Mark Vander Weg, Teresa Hudson Pharm, Michelle Mengeling, Mary Vaughan-Sarrazin, and Teresa Hudson. 2018. “Availability of Healthcare Providers for Rural Veterans Eligible for Purchased Care under the Veterans Choice Act.” *BMC Health Services Research* 18(1):N.PAG-N.PAG. doi: [10.1186/s12913-018-3108-8](https://doi.org/10.1186/s12913-018-3108-8).

Ojeda, Victoria D., Michelle R. Munson, Nev Jones, Emily Berliant, and Todd P. Gilmer. 2021. “The Availability of Peer Support and Disparities in Outpatient Mental Health Service Use among Minority Youth with Serious Mental Illness.” *Administration and Policy in Mental Health and Mental Health Services Research* 48(2):290–98. doi: [10.1007/s10488-020-01073-8](https://doi.org/10.1007/s10488-020-01073-8).

Olin, Su-chin Serene, Briannon C. O’Connor, Amy Storfer-Isser, Lisa J. Clark, Matthew Perkins, Sarah Hudson Scholle, Emma D. Whitmyre, Kimberly Hoagwood, Sarah McCue Horwitz, and Briannon C. O’Connor. 2016. “Access to Care for Youth in a State Mental Health System: A Simulated Patient Approach.” *Journal of the American Academy of Child & Adolescent Psychiatry* 55(5):392–99. doi: [10.1016/j.jaac.2016.02.014](https://doi.org/10.1016/j.jaac.2016.02.014).

Ortega, Alexander N., Ryan M. McKenna, Jessie Kemmick Pintor, Brent A. Langellier, Dylan H. Roby, Nadereh Pourat, Arturo Vargas Bustamante, and Steven P. Wallace. 2018. “Health Care Access and Physical and Behavioral Health Among Undocumented Latinos in California.” *Medical Care* 56(11):919–26. doi: [10.1097/MLR.0000000000000985](https://doi.org/10.1097/MLR.0000000000000985).

Orth, Jessica, Yue Li, Adam Simning, and Helena Temkin-Greener. 2020. “Severe Behavioral Health Manifestations in Nursing Homes: Associations with Service Availability?” *Journal of the American Geriatrics Society* 68(11):2643–49. doi: [10.1111/jgs.16772](https://doi.org/10.1111/jgs.16772).

Palmer H. 2007. “Going Mainstream: Improving Access to Counselling Services.” *Learning Disability Practice* 10(7):28–32. doi: [10.7748/ldp2007.09.10.7.28.c4278](https://doi.org/10.7748/ldp2007.09.10.7.28.c4278).

Parcesepe, Angela M., Kathryn Lancaster, E. Jennifer Edelman, Raquel DeBoni, Jeremy Ross, Lukoye Atwoli, Mpho Tlali, Keri Althoff, Judicaël Tine, Stephany N. Duda, C. William Wester, and Denis Nash. 2020. “Substance Use Service Availability in HIV Treatment Programs: Data from the Global IeDEA Consortium, 2014-2015 and 2017.” *PloS One* 15(8):e0237772. doi: [10.1371/journal.pone.0237772](https://doi.org/10.1371/journal.pone.0237772).

Parnes, McKenna F., Rohini Bagrodia, Katie Wightman, Ria Singh-Sawhney, Margaret L. Satterthwaite, Sarah Knuckey, Richard A. Bryant, and Adam D. Brown. 2020. “Posttraumatic Stress Symptoms and Access to Services among Human Rights Advocates: The Mediating Roles of Organizational Encouragement of Support Seeking and Occupation-Related Appraisals.” *Psychological Services* 17(2):170–77. doi: [10.1037/ser0000359](https://doi.org/10.1037/ser0000359).

Parran, Theodore V., Joseph Z. Muller, Elina Chernyak, Chris Adelman, Christina M. Delos Reyes, Douglas Rowland, and Mykola Kolganov. 2017. “Access to and Payment for Office-Based Buprenorphine Treatment in Ohio.” *Substance Abuse: Research and Treatment* 11:1178221817699247. doi: [10.1177/1178221817699247](https://doi.org/10.1177/1178221817699247).

Perron, Brian E., Christopher N. Jarman, and Amy M. Kilbourne. 2009. “Access to Conventional Mental Health and Medical Care Among Users of Complementary and Alternative Medicine With Bipolar Disorder.” *Journal of Nervous and Mental Disease* 197(4):287–90. doi: [10.1097/NMD.0b013e31819dc16a](https://doi.org/10.1097/NMD.0b013e31819dc16a).

Phillips, Mark, Kora DeBeck, Timothy Desjarlais, Tracey Morrison, Cindy Feng, Thomas Kerr, and Evan Wood. 2014. “Inability to Access Addiction Treatment Among Street-Involved Youth in a Canadian Setting.” *Substance Use & Misuse* 49(10):1233–40. doi: [10.3109/10826084.2014.891618](https://doi.org/10.3109/10826084.2014.891618).

Pomerantz A, Cole BH, Watts BV, and Weeks WB. 2008. “Improving Efficiency and Access to Mental Health Care: Combining Integrated Care and Advanced Access.” *General Hospital Psychiatry* 30(6):546–51. doi: [10.1016/j.genhosppsych.2008.09.004](https://doi.org/10.1016/j.genhosppsych.2008.09.004).

Prina, A. Matthew, Riccardo E. Marioni, Geoffrey C. Hammond, Peter B. Jones, Carol Brayne, and Tom Dening. 2014. “Improving Access to Psychological Therapies and Older People: Findings from the Eastern Region.” *Behaviour Research and Therapy* 56:75–81. doi: [10.1016/j.brat.2014.03.008](https://doi.org/10.1016/j.brat.2014.03.008).

Pyne, Jeffrey M., P. Adam Kelly, Ellen P. Fischer, Christopher J. Miller, Samantha L. Connolly, Patricia Wright, Kara Zamora, Christopher J. Koenig, Karen H. Seal, and John C. Fortney. 2022. “Initial Concurrent and Convergent Validity of the Perceived Access Inventory (PAI) for Mental Health Services.” *Psychological Services* 19(1):118–24. doi: [10.1037/ser0000504](https://doi.org/10.1037/ser0000504).

Pyne, Jeffrey M., P. Adam Kelly, Ellen P. Fischer, Christopher J. Miller, Patricia Wright, Kara Zamora, Christopher J. Koenig, Regina Stanley, Karen Seal, and John C. Fortney. 2019. “Development of a Perceived Access Inventory for Community Care Mental Healthcare Services for Veterans.” *Military Medicine* 184(7/8):e301–8. doi: [10.1093/milmed/usy429](https://doi.org/10.1093/milmed/usy429).

Qeadan, Fares, Nana Akofua Mensah, Lily Y. Gu, William A. Barbeau, Erin Fanning Madden, Christina A. Porucznik, and Kevin English. 2021. “Factors Associated with the Availability of Tailored Programs for Lgbt Clients in Substance Use Disorder Treatment Facilities in the US From 2008 to 2018.” *Journal of Gay & Lesbian Social Services: The Quarterly Journal of Community & Clinical Practice*. doi: [10.1080/10538720.2021.1954125](https://doi.org/10.1080/10538720.2021.1954125).

Radusky, Pablo D., Nadir Cardozo, Mariana Duarte, Solange Fabian, Emilia Frontini, Omar Sued, and Ines Aristegui. n.d. “Mental Health, Substance Use, Experiences of Violence, and Access to Health Care among Transgender and Non-Binary People during the COVID-19 Lockdown in Argentina.” *International Journal of Transgender Health*. doi: [10.1080/26895269.2021.1943593](https://doi.org/10.1080/26895269.2021.1943593).

Reinhart, Crystal A., Maria Sae-Hau, Carol A. Lee, and Elisa S. Weiss. 2020. “Blood Cancer Survivorship in NCI-Designated Cancer Centers: A Study of Services, Gaps, and Access Barriers.” *Journal of Cancer Survivorship* 14(1):43–47. doi: [10.1007/s11764-019-00823-4](https://doi.org/10.1007/s11764-019-00823-4).

Reschovsky JD and Staiti AB. 2005. “Access and Quality: Does Rural America Lag behind? In 2000-01, the Overall Rural Supply of Providers Was Adequate to Provide Access to Care on Par with That in Urban Areas.” *Health Affairs* 24(4):1128–39. doi: [10.1377/hlthaff.24.4.1128](https://doi.org/10.1377/hlthaff.24.4.1128).

Rhodes KV, Vieth TL, Kushner H, Levy H, and Asplin BR. 2009. “Referral without Access: For Psychiatric Services, Wait for the Beep.” *Annals of Emergency Medicine* 54(2):272–78. doi: [10.1016/j.annemergmed.2008.08.023](https://doi.org/10.1016/j.annemergmed.2008.08.023).

Ridings, Leigh E., Hannah C. Espeleta, Christian J. Streck, Tatiana M. Davidson, Nicole Litvitskiy, Olivia Bravoco, Nancy Kassam-Adams, and Kenneth J. Ruggiero. 2022. “Assessing Service Quality and Access in Trauma Centers through Behavioral Health Screening, Education, and Treatment after Pediatric Injury.” *Journal of Pediatric Surgery* 57(11):632–36. doi: [10.1016/j.jpedsurg.2022.01.014](https://doi.org/10.1016/j.jpedsurg.2022.01.014).

Rivers, J. E., E. Komaroff, and A. C. Kibort. 1999. “Access to Health and Human Services for Drug Users: An Urban/Rural Community Systems Perspective.” *Substance Use & Misuse* 34(4–5):707–25. doi: [10.3109/10826089909037239](https://doi.org/10.3109/10826089909037239).

Rocks, Stephen, Mina Fazel, and Apostolos Tsiachristas. 2020. “Impact of Transforming Mental Health Services for Young People in England on Patient Access, Resource Use and Health: A Quasi-Experimental Study.” *BMJ Open* 10(1):e034067. doi: [10.1136/bmjopen-2019-034067](https://doi.org/10.1136/bmjopen-2019-034067).

Rocks, Stephen, Margaret Glogowska, Melissa Stepney, Apostolos Tsiachristas, and Mina Fazel. 2020. “Introducing a Single Point of Access (SPA) to Child and Adolescent Mental Health Services in England: A Mixed-Methods Observational Study.” *BMC Health Services Research* 20(1):1–11. doi: [10.1186/s12913-020-05463-4](https://doi.org/10.1186/s12913-020-05463-4).

Rosenberg, Harold, John Melville, and P. C. McLean. 2002. “Acceptability and Availability of Pharmacological Interventions for Substance Misuse by British NHS Treatment Services.” *Addiction (Abingdon, England)* 97(1):59–65. doi: [10.1046/j.1360-0443.2002.00059.x](https://doi.org/10.1046/j.1360-0443.2002.00059.x).

Rosenberg, Harold, and Kristina T. Phillips. 2003. “Acceptability and Availability of Harm-Reduction Interventions for Drug Abuse in American Substance Abuse Treatment Agencies.” *Psychology of Addictive Behaviors : Journal of the Society of Psychologists in Addictive Behaviors* 17(3):203–10. doi: [10.1037/0893-164X.17.3.203](https://doi.org/10.1037/0893-164X.17.3.203).

Rosenheck, Robert, Douglas Leslie, and George Woody. 2003. “Fiscal Strain and Access to Opiate Substitution Therapy at Department of Veterans Affairs Medical Centers.” *The American Journal on Addictions* 12(3):220–28.

Rush, Kathy L., Cherisse Seaton, Eric Li, Nelly D. Oelke, and Barbara Pesut. 2021. “Rural Use of Health Service and Telemedicine during COVID-19: The Role of Access and EHealth Literacy.” *Health Informatics Journal* 27(2):14604582211020064. doi: [10.1177/14604582211020064](https://doi.org/10.1177/14604582211020064).

Russo, Patricia Anne. 1997. “The Effect of Mental Morbidity and Comorbidity on Access to and Utilization of Health Care Services by Non-Institutionalized Medicare Recipients.” ProQuest Information & Learning.

Salvador-Carulla, Luis, Sandra Saldivia, Rafael Martinez-Leal, Benjamin Vicente, Carlos Garcia-Alonso, Pamela Grandon, and Josep Maria Haro. 2008. “Meso-Level Comparison of Mental Health Service Availability and Use in Chile and Spain.” *Psychiatric Services* 59(4):421–28. doi: [10.1176/appi.ps.59.4.421](https://doi.org/10.1176/appi.ps.59.4.421).

Scheyett, Anna, Jennie Vaughn, and Melissa Floyd Taylor. 2009. “Screening and Access to Services for Individuals with Serious Mental Illnesses in Jails.” *Community Mental Health Journal* 45(6):439–46. doi: [10.1007/s10597-009-9204-9](https://doi.org/10.1007/s10597-009-9204-9).

Schlesinger, M., R. Dorwart, C. Hoover, and S. Epstein. 1997. “Competition, Ownership, and Access to Hospital Services - Evidence from Psychiatric Hospitals.” *Medical Care* 35(9):974–92. doi: [10.1097/00005650-199709000-00009](https://doi.org/10.1097/00005650-199709000-00009).

Seo, Veri, Travis P. Baggett, Anne N. Thorndike, Peter Hull, John Hsu, Joseph P. Newhouse, and Vicki Fung. 2019. “Access to Care among Medicaid and Uninsured Patients in Community Health Centers after the Affordable Care Act.” *BMC Health Services Research* 19(1):291. doi: [10.1186/s12913-019-4124-z](https://doi.org/10.1186/s12913-019-4124-z).

Seshadri, Roopa, Douglas Strane, Meredith Matone, Karen Ruedisueli, and David M. Rubin. 2019. “Families With TRICARE Report Lower Health Care Quality And Access Compared To Other Insured And Uninsured Families.” *Health Affairs* 38(8):1377–85. doi: [10.1377/hlthaff.2019.00274](https://doi.org/10.1377/hlthaff.2019.00274).

Shah, Amar, Auzewell Chitewe, Emma Binley, Forid Alom, and James Innes. 2018. “Improving Access to Services through a Collaborative Learning System at East London NHS Foundation Trust.” *BMJ Open Quality* 7(3):e000337. doi: [10.1136/bmjoq-2018-000337](https://doi.org/10.1136/bmjoq-2018-000337).

Shover, Chelsea L. 2021. “Availability of Extended-Release Buprenorphine to Treat Opioid Use Disorders among Medicaid-Covered Patients.” *Psychiatric Services* 72(2):225–26. doi: [10.1176/appi.ps.202000165](https://doi.org/10.1176/appi.ps.202000165).

Shover, Chelsea L., and Keith Humphreys. 2019. “Predictors of Availability of Long-Acting Medication for Opioid Use Disorder.” *Drug and Alcohol Dependence* 204:107586. doi: [10.1016/j.drugalcdep.2019.107586](https://doi.org/10.1016/j.drugalcdep.2019.107586).

Silverman, Alexandra L., and Bethany A. Teachman. 2022. “The Relationship between Access to Mental Health Resources and Use of Preferred Effective Mental Health Treatment.” *Journal of Clinical Psychology* 78(6):1020–45. doi: [10.1002/jclp.23301](https://doi.org/10.1002/jclp.23301).

Simpson, Chris, and Prasanna de Silva. 2003. “Multi-Disciplinary Team Assessments: A Method of Improving the Quality and Accessibility of Old Age Psychiatry Services.” *Psychiatric Bulletin* 27(9):346–48. doi: [10.1192/pb.27.9.346](https://doi.org/10.1192/pb.27.9.346).

Slade, Eric P. 2003. “The Relationship between School Characteristics and the Availability of Mental Health and Related Health Services in Middle and High Schools in the United States.” *The Journal of Behavioral Health Services & Research* 30(4):382–92. doi: [10.1007/BF02287426](https://doi.org/10.1007/BF02287426).

Smith, Clifford A., Matthew Tyler Boden, and Jodie A. Trafton. 2023. “Outpatient Provider Staffing Ratios: Binary Recursive Models Associated with Quality, Access, and Satisfaction.” *Psychological Services* 20(1):137–43. doi: [10.1037/ser0000449](https://doi.org/10.1037/ser0000449).

Smith, Kelley, Janet Kuramoto-Crawford, and Sean Lynch. 2013. “Availability of Payment Assistance for Mental Health Services in U.S. Mental Health Treatment Facilities.” in *The CBHSQ Report*. Rockville (MD): Substance Abuse and Mental Health Services Administration (US).

Solberg, Leif I., A. Lauren Crain, Jo Ann M. Sperl-Hillen, Mary C. Hroscikoski, Karen I. Engebretson, and Patrick J. O’Connor. 2006. “Effect of Improved Primary Care Access on Quality of Depression Care.” *Annals of Family Medicine* 4(1):69–74. doi: [10.1370/afm.426](https://doi.org/10.1370/afm.426).

Solomon, Keisha T., Sachini Bandara, Ian S. Reynolds, Noa Krawczyk, Brendan Saloner, Elizabeth Stuart, and Elizabeth Connolly. 2022. “Association between Availability of Medications for Opioid Use Disorder in Specialty Treatment and Use of Medications among Patients: A State-Level Trends Analysis.” *Journal of Substance Abuse Treatment* 132:108424. doi: [10.1016/j.jsat.2021.108424](https://doi.org/10.1016/j.jsat.2021.108424).

Sorensen, James L., Joseph Guydish, Pamela Zilavy, Thomas B. Davis, Alice Gleghorn, Marvin Jacoby, and Clare Sears. 2007. “Access to Drug Abuse Treatment under Treatment on Demand Policy in San Francisco.” *The American Journal of Drug and Alcohol Abuse* 33(2):227–36. doi: [10.1080/00952990601174824](https://doi.org/10.1080/00952990601174824).

van Spijker, Bregje A., Jose A. Salinas-Perez, John Mendoza, Tanya Bell, Nasser Bagheri, Mary Anne Furst, Julia Reynolds, Daniel Rock, Andrew Harvey, Alan Rosen, and Luis Salvador-Carulla. 2019. “Service Availability and Capacity in Rural Mental Health in Australia: Analysing Gaps Using an Integrated Mental Health Atlas.” *The Australian and New Zealand Journal of Psychiatry* 53(10):1000–1012. doi: [10.1177/0004867419857809](https://doi.org/10.1177/0004867419857809).

Spivak, Stanislav, Bernadette A. Cullen, Charee Green, Tyler Firth, Holly Sater, and Ramin Mojtabai. 2019. “Availability of Assertive Community Treatment in the United States: 2010 to 2016.” *Psychiatric Services* 70(10):948–51. doi: [10.1176/appi.ps.201900032](https://doi.org/10.1176/appi.ps.201900032).

Stafford, Jamie, Marco Aurelio, and Amar Shah. 2020. “Improving Access and Flow within Child and Adolescent Mental Health Services: A Collaborative Learning System Approach.” *BMJ Open Quality* 9(4). doi: [10.1136/bmjoq-2019-000832](https://doi.org/10.1136/bmjoq-2019-000832).

Stargatt, Jennifer, Sunil S. Bhar, Tanya E. Davison, Nancy A. Pachana, Leander Mitchell, Deborah Koder, Carol Hunter, Colleen Doyle, Yvonne Wells, and Edward Helmes. 2017. “The Availability of Psychological Services for Aged Care Residents in Australia: A Survey of Facility Staff.” *Australian Psychologist* 52(6):406–13. doi: [10.1111/ap.12244](https://doi.org/10.1111/ap.12244).

Steinman, Kenneth J., Kelly Kelleher, Allard E. Dembe, Thomas M. Wickizer, and Traci Hemming. 2012. “The Use of a ‘Mystery Shopper’ Methodology to Evaluate Children’s Access to Psychiatric Services.” *The Journal of Behavioral Health Services & Research* 39(3):305–13. doi: [10.1007/s11414-012-9275-1](https://doi.org/10.1007/s11414-012-9275-1).

Svenson, L. W. 1990. “Mental Health Services in Edmonton: An Assessment of Service Availability.” *Canadian Journal of Public Health = Revue Canadienne de Sante Publique* 81(5):394–95.

Taman, M. S., and F. E. Menz. 1997. “Professional Perceptions of Availability and Quality of Mental Health Services in Wisconsin.” *Wisconsin Medical Journal* 96(1):40–45.

Taylor, Kira-Jai Jayne. 2022. “Role of Case Management in Access to Mental Health Care among African Americans with Medicaid.” ProQuest Information & Learning.

Teles, Soraia, Ana Ferreira, and Constança Paúl. 2021. “Access and Retention of Informal Dementia Caregivers in Psychosocial Interventions: A Cross-Sectional Study.” *Archives of Gerontology and Geriatrics* 93:104289. doi: [10.1016/j.archger.2020.104289](https://doi.org/10.1016/j.archger.2020.104289).

Thomas, Neil, Fiona Foley, Katrina Lindblom, and Stuart Lee. 2017. “Are People with Severe Mental Illness Ready for Online Interventions? Access and Use of the Internet in Australian Mental Health Service Users.” *Australasian Psychiatry: Bulletin of Royal Australian and New Zealand College of Psychiatrists* 25(3):257–61. doi: [10.1177/1039856217689913](https://doi.org/10.1177/1039856217689913).

Ting L, Jacobson JM, and Sanders S. 2008. “Available Supports and Coping Behaviors of Mental Health Social Workers Following Fatal and Nonfatal Client Suicidal Behavior.” *Social Work* 53(3):211–21. doi: [10.1093/sw/53.3.211](https://doi.org/10.1093/sw/53.3.211).

Tirintica, Andreea Raluca, Ivana Andjelkovic, Orela Sota, Mihail Cristian Pirlog, Maria Stoyanova, Adriana Mihai, and Neal Wallace. 2018. “Factors That Influence Access to Mental Health Services in South-Eastern Europe.” *International Journal of Mental Health Systems* 12:75. doi: [10.1186/s13033-018-0255-6](https://doi.org/10.1186/s13033-018-0255-6).

Too, Lay San, Liana Leach, and Peter Butterworth. 2020. “Mental Health Problems and Internet Access: Results From an Australian National Household Survey.” *JMIR Mental Health* 7(5):e14825. doi: [10.2196/14825](https://doi.org/10.2196/14825).

Valdimarsdóttir, Unnur, Ásgeir R. Helgason, Carl Johan Fürst, Jan Adolfsson, and Gunnar Steineck. 2005. “Need for and Access to Bereavement Support after Loss of a Husband to Urologic Cancers: A Nationwide Follow-up of Swedish Widows.” *Scandinavian Journal of Urology and Nephrology* 39(4):271–76. doi: [10.1080/00365590510031273](https://doi.org/10.1080/00365590510031273).

Van Voorhees BW, Wang N, and Ford DE. 2003. “Managed Care Organizational Complexity and Access to High-Quality Mental Health Services: Perspective of U.S. Primary Care Physicians.” *General Hospital Psychiatry* 25(3):149–57. doi: [10.1016/s0163-8343(03)00017-3](https://doi.org/10.1016/s0163-8343(03)00017-3).

VanderWielen, Lynn M., Emma C. Gilchrist, Molly A. Nowels, Stephen M. Petterson, George Rust, and Benjamin F. Miller. 2015. “Not Near Enough: Racial and Ethnic Disparities in Access to Nearby Behavioral Health Care and Primary Care.” *Journal of Health Care for the Poor & Underserved* 26(3):1032–47. doi: [10.1353/hpu.2015.0083](https://doi.org/10.1353/hpu.2015.0083).

Voon, Pauline, Linwei Wang, Ekaterina Nosova, Kanna Hayashi, Michael John Milloy, Evan Wood, and Thomas Kerr. 2020. “Greater Pain Severity Is Associated with Inability to Access Addiction Treatment Among a Cohort of People Who Use Drugs.” *Journal of Pain Research* 13:2443–49. doi: [10.2147/JPR.S255438](https://doi.org/10.2147/JPR.S255438).

Voss CP, Cesar KW, Tymus T, and Fiedler IG. 2002. “Perceived versus Actual Physical Accessibility of Substance Abuse Treatment Facilities.” *Topics in Spinal Cord Injury Rehabilitation* 7(3):47–55.

Waliski, Angela Dawn. 2002. “An Examination of Sexual Assault Agencies and the Services Available to Male Victims.” ProQuest Information & Learning.

Watkins, Katherine E., Donna J. Keyser, Brad Smith, Thomas E. Mannle, Daniel R. Kivlahan, Susan M. Paddock, Teryn Mattox, Marcela Horvitz-Lennon, and Harold Alan Pincus. 2010. “Transforming Mental Healthcare in the Veterans Health Administration: A Model for Measuring Performance to Improve Access, Quality, and Outcomes.” *Journal for Healthcare Quality: Promoting Excellence in Healthcare* 32(6):33–43. doi: [10.1111/j.1945-1474.2010.00109.x](https://doi.org/10.1111/j.1945-1474.2010.00109.x).

Way, William C., Ashley M. Coker-Cranney, and Jack C. Watson. 2020. “‘So Many Mental Health Issues Go Unsaid’: Implications for Best Practice Guidelines From Student-Athletes’ Perspectives About Service Availability.” *Journal of Clinical Sport Psychology* 14(3):305–24. doi: [10.1123/jcsp.2019-0051](https://doi.org/10.1123/jcsp.2019-0051).

Weinzimmer, Laurence G., Matthew D. Dalstrom, Colleen J. Klein, Roopa Foulger, and Sarah S. de Ramirez. 2021. “The Relationship between Access to Mental Health Counseling and Interest in Rural Telehealth.” *Journal of Rural Mental Health* 45(3):219–28. doi: [10.1037/rmh0000179](https://doi.org/10.1037/rmh0000179).

Welbel, Marta, Aleksandra Matanov, Jacek Moskalewicz, Henrique Barros, Reamonn Canavan, Edina Gabor, Andrea Gaddini, Tim Greacen, Ulrike Kluge, Vincent Lorant, Mercedes Esteban Peña, Aart H. Schene, Joaquim J. F. Soares, Christa Straßmayr, Petra Vondráčkov, and Stefan Priebe. 2013. “Addiction Treatment in Deprived Urban Areas in EU Countries: Accessibility of Care for People from Socially Marginalized Groups.” *Drugs: Education, Prevention & Policy* 20(1):74–83. doi: [10.3109/09687637.2012.706757](https://doi.org/10.3109/09687637.2012.706757).

Wen, Hefei, Janet R. Cummings, Jason M. Hockenberry, Laura M. Gaydos, and Benjamin G. Druss. 2013. “State Parity Laws and Access to Treatment for Substance Use Disorder in the United States: Implications for Federal Parity Legislation.” *JAMA Psychiatry* 70(12):1355–62. doi: [10.1001/jamapsychiatry.2013.2169](https://doi.org/10.1001/jamapsychiatry.2013.2169).

Wen, Hefei, Benjamin G. Druss, and Janet R. Cummings. 2015. “Effect of Medicaid Expansions on Health Insurance Coverage and Access to Care among Low-Income Adults with Behavioral Health Conditions.” *Health Services Research* 50(6):1787–1809. doi: [10.1111/1475-6773.12411](https://doi.org/10.1111/1475-6773.12411).

West, Joyce C., Diana E. Clarke, Farifteh Firoozmand Duffy, Keila D. Barber, Ramin Mojtabai, Eve K. Mościcki, Kristin Kroeger Ptakowski, and Saul Levin. 2016. “Availability of Mental Health Services Prior to Health Care Reform Insurance Expansions.” *Psychiatric Services (Washington, D.C.)* 67(9):983–89. doi: [10.1176/appi.ps.201500423](https://doi.org/10.1176/appi.ps.201500423).

West, Joyce C., Joshua E. Wilk, Farifteh F. Duffy, S. Janet Kuramoto, Donald S. Rae, Eve K. Moscicki, and Charles W. Hoge. 2014. “Mental Health Treatment Access and Quality in the Army: Survey of Mental Health Clinicians.” *Journal of Psychiatric Practice®* 20(6):448–59. doi: [10.1097/01.pra.0000456593.58739.61](https://doi.org/10.1097/01.pra.0000456593.58739.61).

West SL, Graham CW, and Cifu DX. 2009a. “Physical and Programmatic Accessibility of British Alcohol/Other Drug Treatment Centers.” *Alcoholism Treatment Quarterly* 27(3):294–304. doi: [10.1080/07347320903008174](https://doi.org/10.1080/07347320903008174).

West SL, Graham CW, and Cifu DX. 2009b. “Rates of Alcohol/Other Drug Treatment Denials to Persons with Physical Disabilities: Accessibility Concerns.” *Alcoholism Treatment Quarterly* 27(3):305–16. doi: [10.1080/07347320903008190](https://doi.org/10.1080/07347320903008190).

Wilk JE, West JC, Narrow WE, Rae DS, and Regier DA. 2005. “Economic Grand Rounds. Access to Psychiatrists in the Public Sector and in Managed Health Plans” edited by Sharfstein SS and Huskamp HA. *Psychiatric Services* 56(4):408–10. doi: [10.1176/appi.ps.56.4.408](https://doi.org/10.1176/appi.ps.56.4.408).

Williams, Natasha D., and Jessica N. Fish. 2020. “The Availability of LGBT-Specific Mental Health and Substance Abuse Treatment in the United States.” *Health Services Research* 55(6):932–43. doi: [10.1111/1475-6773.13559](https://doi.org/10.1111/1475-6773.13559).

Williams, Natasha D., Rodman E. Turpin, Ellesse-Roselee L. Akré, Bradley O. Boekeloo, and Jessica N. Fish. 2022. “Disparities in Mental Health Care Access Among Persons Differing in Sexual Identity: Nationally Representative Findings.” *Psychiatric Services (Washington, D.C.)* 73(4):456–59. doi: [10.1176/appi.ps.202100045](https://doi.org/10.1176/appi.ps.202100045).

Wolff, N., and M. Schlesinger. 1998. “Access, Hospital Ownership, and Competition between for-Profit and Nonprofit Institutions.” *Nonprofit and Voluntary Sector Quarterly* 27(2):203–36. doi: [10.1177/0899764098272006](https://doi.org/10.1177/0899764098272006).

Wooten, Adam F. 2002. “Access to Mental Health Services at Veterans Affairs Community-Based Outpatient Clinics.” *Military Medicine* 167(5):424–26.

Wothe, Jillian, Laura Bosacker, Harika Nalluri, Michael J. Cullen, and Melissa E. Brunsvold. 2022. “Improving Mental Health Support, Career Transitions and Access to Health Care for Surgical Residents.” *Journal of Surgical Education* 79(2):286–89. doi: [10.1016/j.jsurg.2021.09.014](https://doi.org/10.1016/j.jsurg.2021.09.014).

Zeber JE, Copeland LA, McCarthy JF, Bauer MS, and Kilbourne AM. 2009. “Perceived Access to General Medical and Psychiatric Care among Veterans with Bipolar Disorder.” *American Journal of Public Health* 99(4):720–27. doi: [10.2105/AJPH.2007.131318](https://doi.org/10.2105/AJPH.2007.131318).

Zimmermann-Schlegel, Verena, Mechthild Hartmann, Halina Sklenarova, Wolfgang Herzog, and Markus W. Haun. 2017. “Accessibility, Availability, and Potential Benefits of Psycho-Oncology Services: The Perspective of Community-Based Physicians Providing Cancer Survivorship Care.” *Oncologist* 22(6):719–27. doi: [10.1634/theoncologist.2016-0245](https://doi.org/10.1634/theoncologist.2016-0245).
